# Halogenation of arenes using alkali metal halides/Fe(NO_3_)_3_·9H_2_O at room temperature†

**DOI:** 10.1039/d5ra00837a

**Published:** 2025-03-19

**Authors:** Caicui Li, Yao Cheng, Fudan Pang, Xiushuo Yan, Zhengtao Huang, Xinmei Wang, Xiaodan Wang, Yiying Li, Jinhui Wang, Huanjun Xu

**Affiliations:** a Department of Medicinal Chemistry and Natural Medicine Chemistry, College of Pharmacy, Harbin Medical University Harbin China 15999290001@126.com; b School of Science, Qiongtai Normal University Haikou 571127 China 15798946232@163.com; c Key Laboratory of Tropical Translational Medicine of Ministry of Education, Key Laboratory of Brain Science Research Transformation in Tropical Environment of Hainan Province, School of Basic Medicine and Life Sciences, Hainan Medical University Haikou China liyiying_hy@163.com

## Abstract

A simple, efficient and environmentally friendly methodology for the halodecarboxylation of anisole analogues using Fe(NO_3_)_3_·9H_2_O/KBr or NaI at room temperature was developed. In this method, most substrates with an electron-donating group afforded corresponding products in good to excellent yields, whereas those with an electron-withdrawing group afforded low to moderate yields. More importantly, this protocol was also applicable for gram-scale synthesis. It is hoped that this methodology will be highly useful in organic synthesis.

## Abstract 2

A simple, efficient and environmentally friendly methodology for the halodecarboxylation of anisole analogues using Fe(NO_3_)_3_·9H_2_O/KBr or NaI at room temperature was developed.

## Graphical abstract

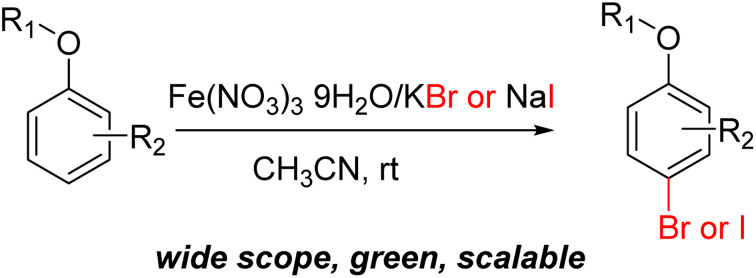

## Introduction

Aryl halides are very common structural units widely present in a large number of natural products, industrial chemicals and pharmaceuticals.^1^ Additionally, aryl halides are important synthetic blocks to construct various compounds.^2^ A series of cross-coupling reactions including the Heck reaction, Suzuki reaction, Ullmann reaction, Grignard reaction, Stille reaction, Sonogashira reaction, Negishi reaction, Kumada reaction, and Hiyama reaction can be conducted using aryl halides.

However, hazardous, toxic and corrosive molecular halogens are usually used as halogenating agents in the laboratory,^3^*e.g.*, in bromination, where bromine is used as a direct reagent for the synthesis of bromoaromatic compounds, which causes environmental pollution and some undesirable side reactions such as the production of toxic HBr gas, uncontrolled polybromination and lower yields. Thus, many reagents have been developed to replace molecular halogens as the source of electrophilic halide cations. The most common alternative to Br_2_ is NBS,^4^ which is used as a “Br” source *via* electrophilic substitution to obtain brominated compounds, which is an important way to produce bromides. Essentially, bromide anions were reacted with oxidants to generate Br^+^. Further, co-oxidants such as PhI(OAc)_2_,^5^ H_2_O_2_ (ref. 6) and metal oxidants^7^ were developed. Meanwhile, quaternary ammonium salts,^8^ pyridinium salts,^9^ 4-(dimethylamino)pyridine tribromide^10^ and *N*,*N*-dibromotosylamide (TsNBr_2_)^11^ were selected as bromination reagents. In recent years, Lewis or Brønsted acids and Lewis bases have been employed to boost the reactivity of bromination by different research groups.^12^ Nevertheless, most of these methods suffer from limitations such as the requirement for essential transition-metal catalysts, inevitable operational complexity, uncommon bromine sources, and a narrow substrate range. Therefore, efficient preparation of aryl halides under mild and green reaction conditions is a continuing goal for chemists in organic synthesis and several other research fields.

In recent years, considerable attention has been paid to the development of a new route for the construction of halogenated scaffolds by utilizing safe and readily available halide sources such as alkali metal halides (halide = I, Br, and Cl).^13^ A combination of oxidants and bromides such as oxone/NaBr,^14^ CAN/KBr,^15^ CAN/LiBr,^16^ Selectfluor®/KBr,^17^ NaBrO_3_–NaBr,^18^ and NaBr/NaNO_3_/TFA^19^ has been employed in these bromination reactions. We also noted that Fe(NO_3_)_3_·9H_2_O had relatively high Lewis acidity and great catalytic activity.^20^ Meanwhile, Fe(NO_3_)_3_·9H_2_O is a cheap, non-toxic and readily available inorganic oxidant and has been used as an efficient oxidant,^21^ nitro source^22^ and catalyst in cross-coupling reactions.^23^ We also found that I_2_ could be generated *in situ* from Fe(NO_3_)_3_·9H_2_O/NaI in DMSO to catalyze the oxidation process.^24^ Thus, we propose that Fe(NO_3_)_3_·9H_2_O competes with alkali metal halides, showing potential to induce halogenation of arenes. In this study, it was found that Fe(NO_3_)_3_·9H_2_O/KBr exhibits efficiency for the synthesis of *p*-bromoaromatic compounds at room temperature, while Fe(NO_3_)_3_·9H_2_O/KI is efficient for the synthesis of *p*-iodoaromatic compounds. Compared with the previous reports, a Fe(NO_3_)_3_·9H_2_O/alkali metal halide system could synthesize aryl halides at room temperature with high to excellent yields for substrates with an electron-donating group and low to moderate yields for substrates with an electron-withdrawing group. In addition, this protocol was readily scaled up to 15 mmol level without any loss of efficiency.^25^

## Results and discussion

The initial step of the study was the optimization of reaction with Fe(NO_3_)_3_·9H_2_O(0.625 mmol)/KBr(0.625 mmol), using anisole (0.5 mmol) as a model substrate in various solvents for 2 h at room temperature (Table 1, entries 1–11). To our delight, *para*-bromination of anisole proceeded smoothly in CH_3_CN, giving the desired product in a high yield up to 99%. When the reaction was carried out in hexane, CH_2_Cl_2_, and EA, the brominated product was obtained in high yields up to 67%, 98% and 89%, respectively. Then, other types of Br sources such as NaBr, LiBr, CuBr_2_ or FeBr_3_ could also be applied in this reaction to give excellent yields (Table 1, entries 12–15). This indicated that Fe(NO_3_)_3_·9H_2_O combined with other bromine salts also successfully afforded 4-bromoanisole. In order to find the best method for the bromination of anisole, the reaction time and the ratio of Fe(NO_3_)_3_·9H_2_O/KBr were determined, which indicated that the optimum conditions are Fe(NO_3_)_3_·9H_2_O (0.625 mmol)/KBr(0.625 mmol) and a reaction time of 2 h (Table 1, entries 16–24). In addition, the reaction did not occur without Fe(NO_3_)_3_·9H_2_O (Table 1, entry 25). We also tested other types of Fe salts and nitrates, among which the desired product was offered by Cu(NO_3_)_2_ with 29% yield (Table 1, entries 26–31).

**Table 1:** Optimization of reaction conditions^a^

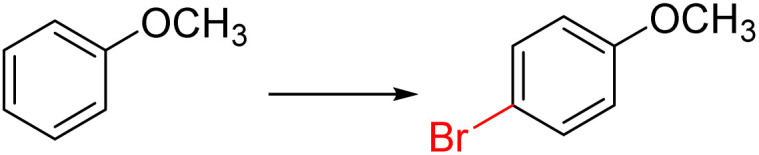					
Entry	Salt	Br-source	*T*/h	Solvent	Yield (%)
1	Fe(NO_3_)_3_·9H_2_O	KBr	2 h	H_2_O	7
2	Fe(NO_3_)_3_·9H_2_O	KBr	2 h	DMSO	9
3	Fe(NO_3_)_3_·9H_2_O	KBr	2 h	Acetone	<1
4	Fe(NO_3_)_3_·9H_2_O	KBr	2 h	DMF	3
5	Fe(NO_3_)_3_·9H_2_O	KBr	2 h	NMP	<1
6	Fe(NO_3_)_3_·9H_2_O	KBr	2 h	Hexane	67
7	Fe(NO_3_)_3_·9H_2_O	KBr	2 h	CH_2_Cl_2_	98
8	Fe(NO_3_)_3_·9H_2_O	KBr	2 h	EA	87
9	Fe(NO_3_)_3_·9H_2_O	KBr	2 h	Methanol	<1
10	Fe(NO_3_)_3_·9H_2_O	KBr	2 h	Ethanol	3
**11**	**Fe(NO**_**3**_ **)**_**3**_ **·9H**_**2**_ **O**	**KBr**	**2 h**	**CH**_**3**_ **CN**	**>99**
12	Fe(NO_3_)_3_·9H_2_O	NaBr	2 h	CH_3_CN	98
13	Fe(NO_3_)_3_·9H_2_O	LiBr	2 h	CH_3_CN	95
14	Fe(NO_3_)_3_·9H_2_O	CuBr_2_	2 h	CH_3_CN	96^b^
15	Fe(NO_3_)_3_·9H_2_O	FeBr_3_	2 h	CH_3_CN	95^c^
16	Fe(NO_3_)_3_·9H_2_O	KBr	1 h	CH_3_CN	57
17	Fe(NO_3_)_3_·9H_2_O	KBr	3 h	CH_3_CN	97
18	Fe(NO_3_)_3_·9H_2_O	KBr	4 h	CH_3_CN	96
19	Fe(NO_3_)_3_·9H_2_O	KBr	2 h	CH_3_CN	95^d^
20	Fe(NO_3_)_3_·9H_2_O	KBr	2 h	CH_3_CN	94^e^
21	Fe(NO_3_)_3_·9H_2_O	KBr	2 h	CH_3_CN	92^f^
22	Fe(NO_3_)_3_·9H_2_O	KBr	2 h	CH_3_CN	29^g^
23	Fe(NO_3_)_3_·9H_2_O	KBr	2 h	CH_3_CN	65^h^
24	Fe(NO_3_)_3_·9H_2_O	KBr	2 h	CH_3_CN	64^i^
25	—	KBr	2 h	CH_3_CN	<1
26	FeCl_3_	KBr	2 h	CH_3_CN	<1
27	FeBr_3_	KBr	2 h	CH_3_CN	<1
28	Fe_2_(SO_4_)_3_	KBr	2 h	CH_3_CN	<1
29	Cu(NO_3_)_2_	KBr	2 h	CH_3_CN	29
30	Co(NO_3_)_2_	KBr	2 h	CH_3_CN	<1
31	Ce(NO_3_)_3_·6H_2_O	KBr	2 h	CH_3_CN	<1

a Anisole (0.5 mmol), salt (0.625 mmol), Br-source (0.625 mmol), rt, GC yield.

b CuBr_2_ (0.313 mmol).

c FeBr_3_ (0.208 mmol).

d Fe(NO_3_)_3_·9H_2_O (0.5 mmol).

e Fe(NO_3_)_3_·9H_2_O (0.375 mmol).

f Fe(NO_3_)_3_·9H_2_O (0.25 mmol).

g Fe(NO_3_)_3_·9H_2_O (0.125 mmol).

h KBr (0.575 mmol).

i KBr (0.525 mmol).

The obtained results for the reaction of *para*-bromination of anisole promoted our procedure to various anisole analogues (Table 2). Gratifyingly, the bromination of arenes containing electron-donating substituents such as alkoxy (Table 2, entries 1–5) and benzyloxy (Table 2, entry 6) proceeded smoothly to give the corresponding *para*-brominated products in good to excellent yields. Naphthyl substrates (Table 2, entries 7 and 8) were found to work well, giving the corresponding product in 86% and 97% yields. It is noteworthy that when 2-methoxynaphthalene reacted with multiple substrates under standard conditions but with the decreasing amount of Fe(NO_3_)_3_·9H_2_O to 0.25 mmol, the yield of desired product was increased to 87%. In addition, phenyl-, methyl-, and ethyl-substituted substrates also gave the corresponding products in high yields with monobromination at the *para* or *ortho* position with respect to the methoxyl substituent (Table 2, entries 9–13). Meanwhile, for weak electron-withdrawing substituted substrates, it offered the corresponding products in a relatively low yield, but when under adjusted conditions, the yield could be improved to a satisfactory value (Table 2, entries 14–19). However, for strong electron-withdrawing substituted substrates such as –CF_3_ and –CN, the reaction did not occur (Table 2, entries 20 and 21).

**Table 2:** Bromination of various substrates^a^

Entry	Substrate	Product	Yield/%
1	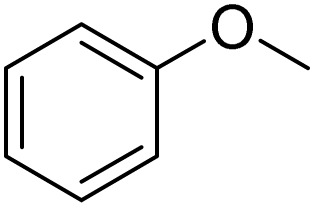	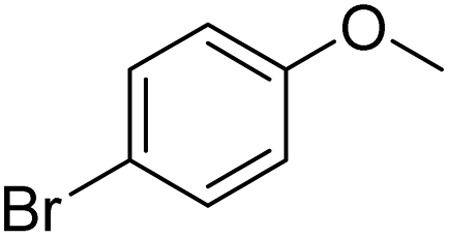	>99
2	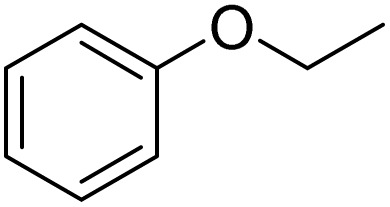	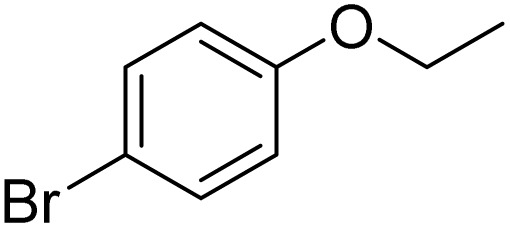	96
3	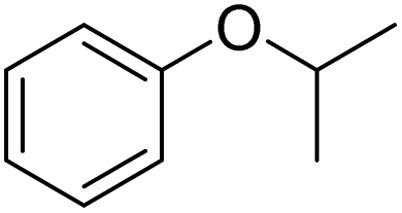	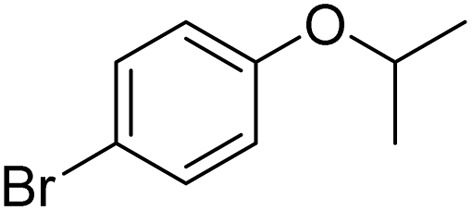	90
4	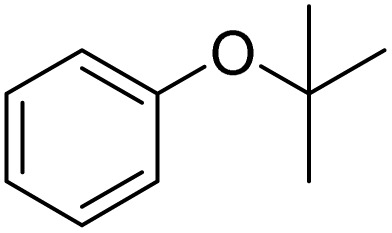	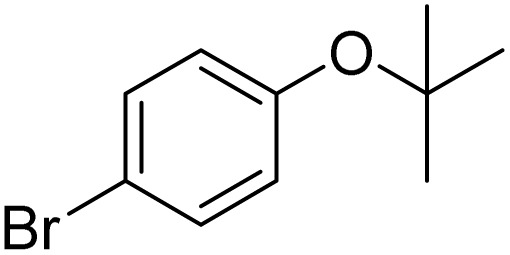	83
5	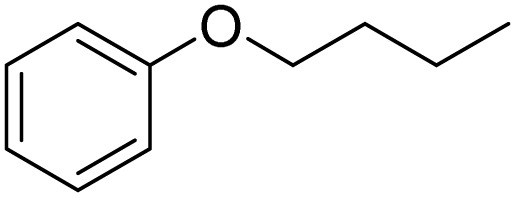	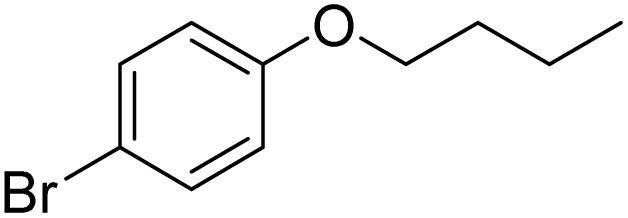	89
6	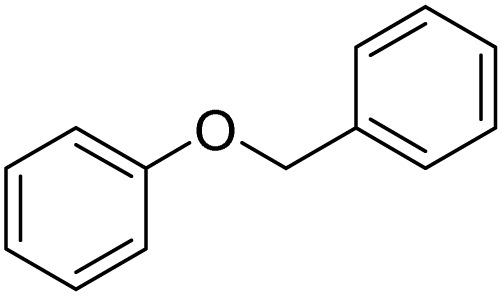	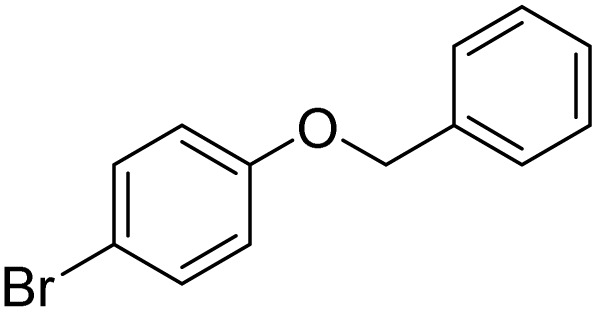	85/99^b^
7	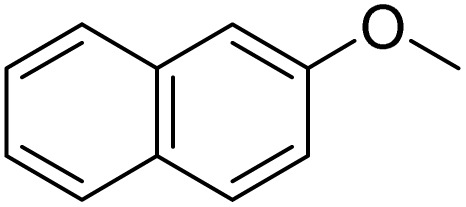	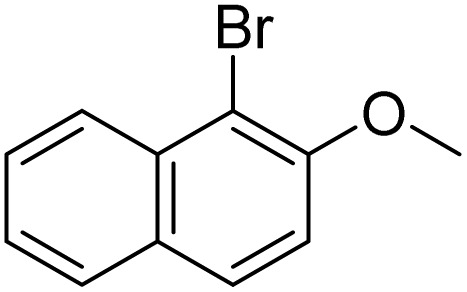	97
8	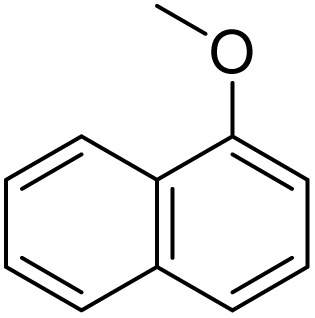	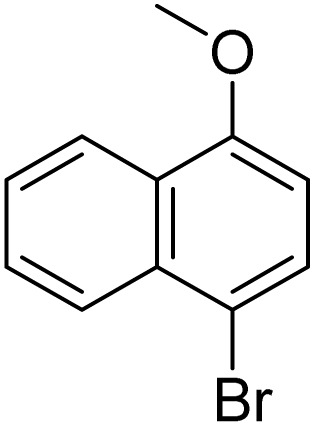	86^c^
9	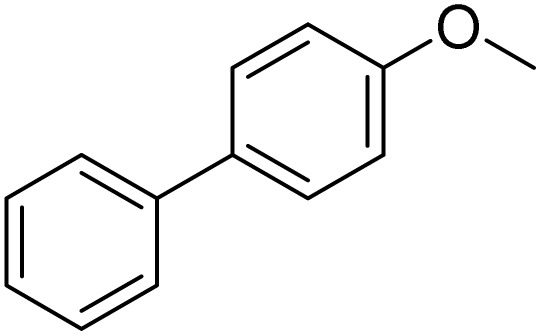	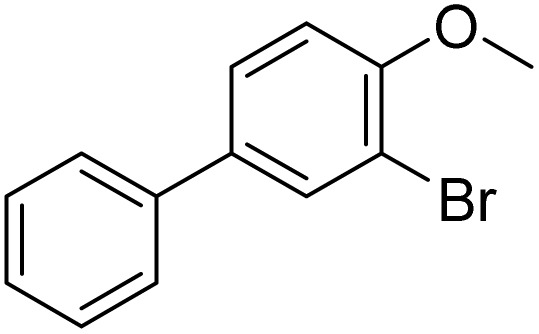	71/91^b^
10	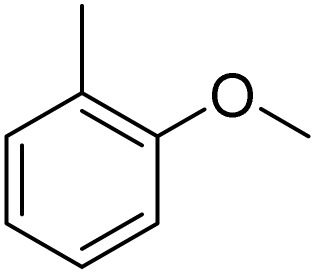	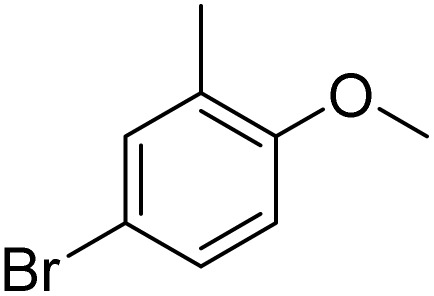	98
11	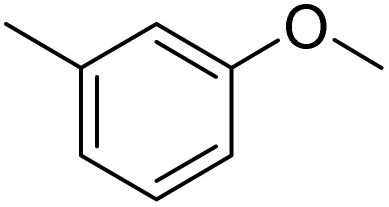	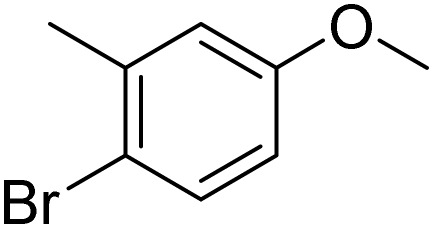	99
12	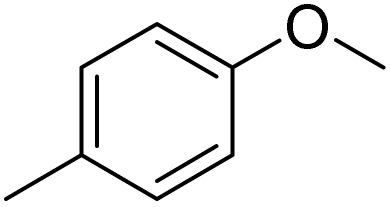	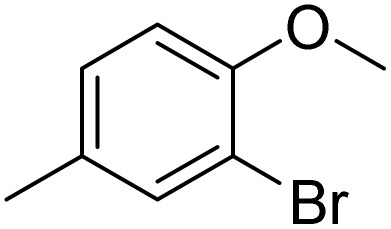	98
13	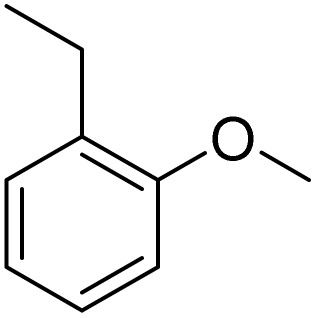	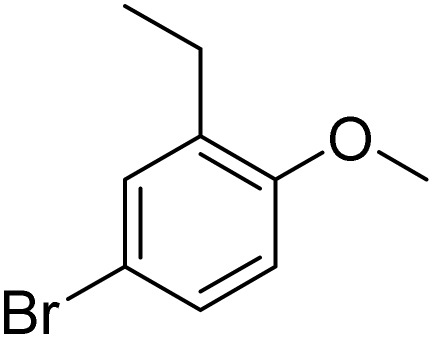	97
14	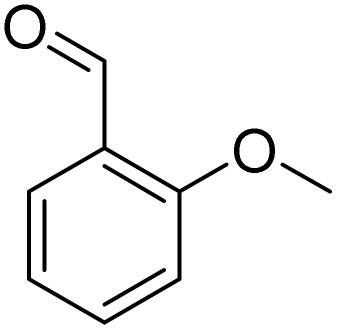	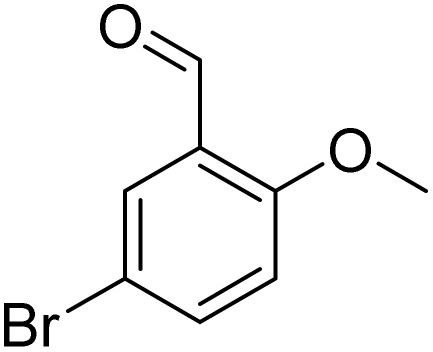	61/93^b^
15	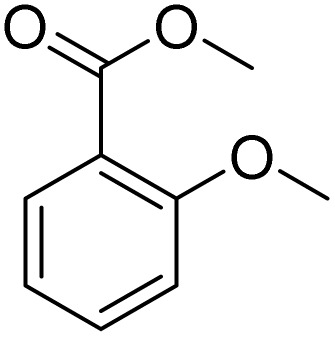	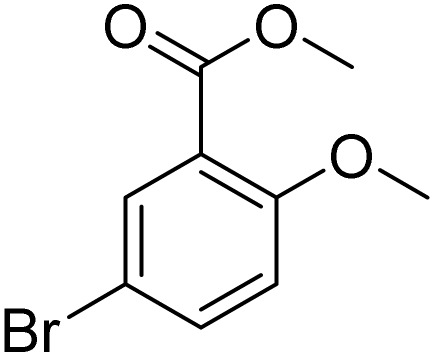	79/96^b^
16	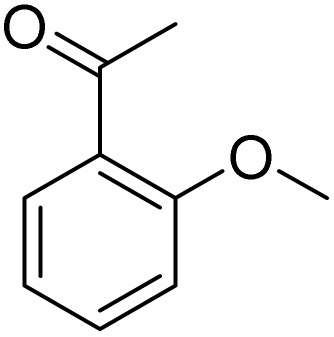	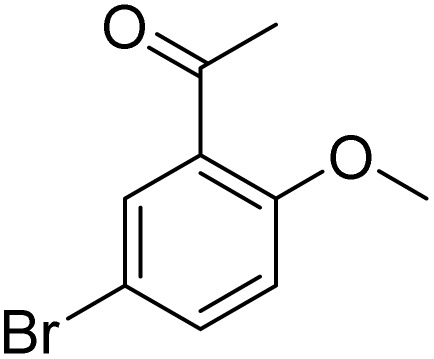	77/91^c^
17	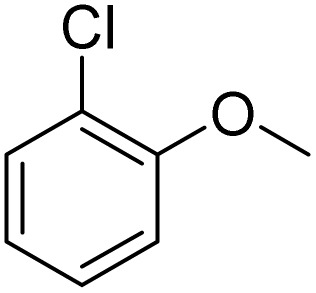	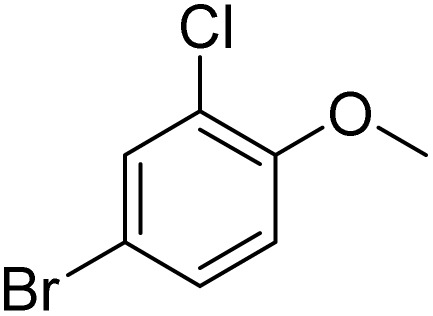	50/96^b^
18	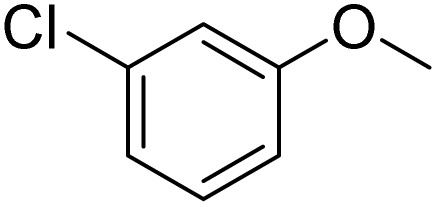	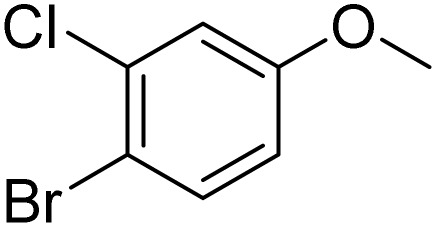	64/96^b^
19	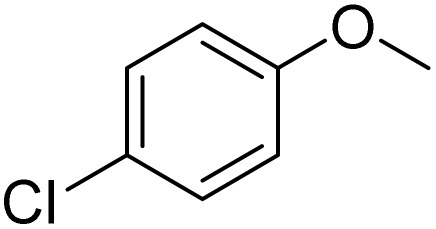	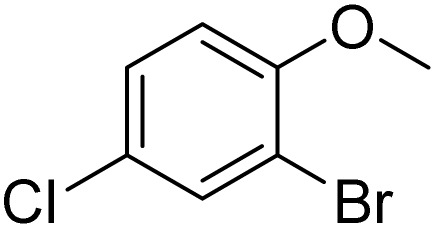	6/68^b^
20	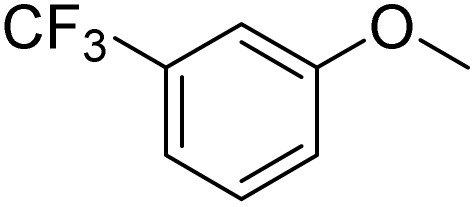	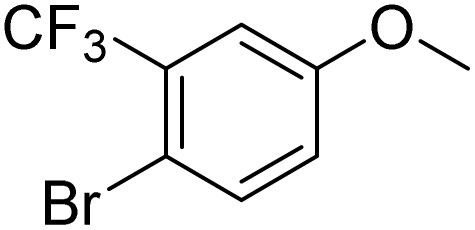	<1
21	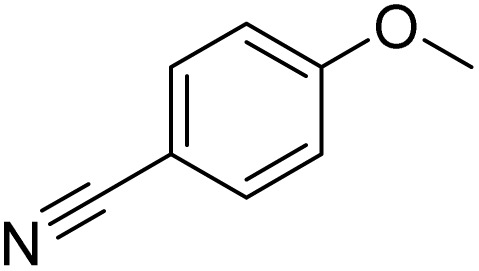	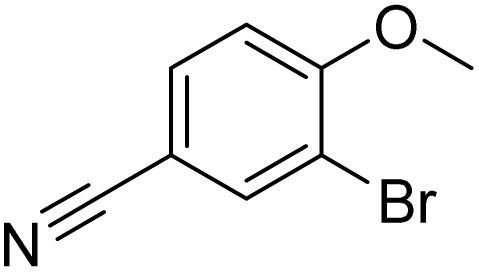	<1
22	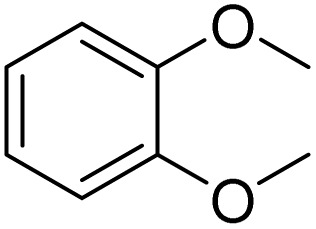	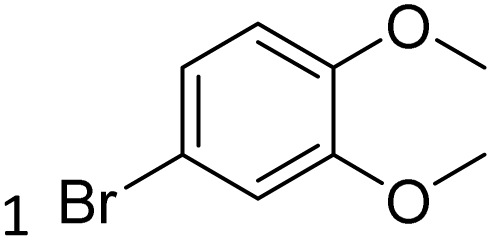	72/15
		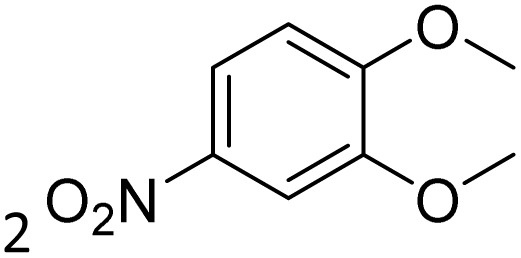	94^d^/5^d^
23	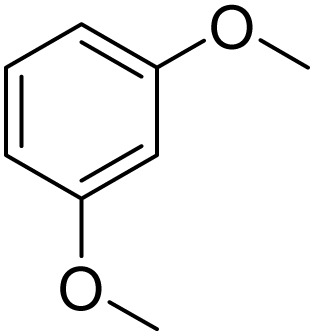	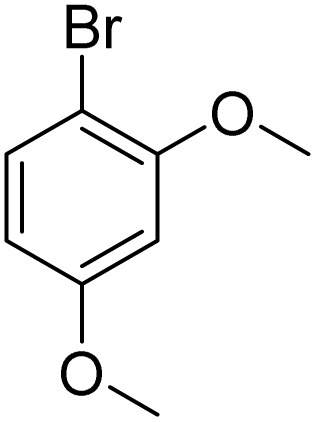	>99^e^
24	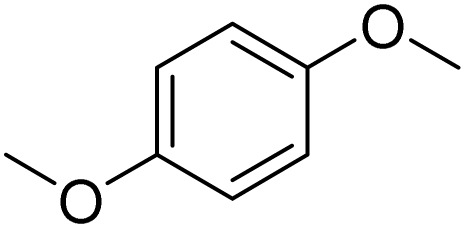	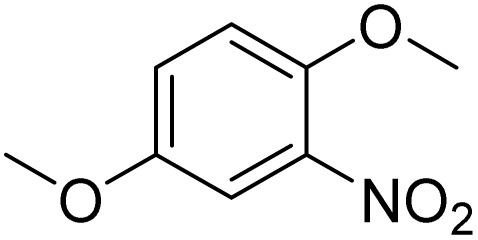	84
25	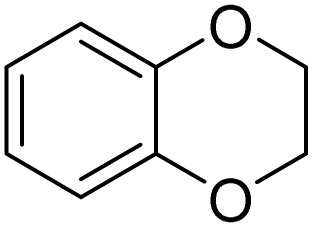	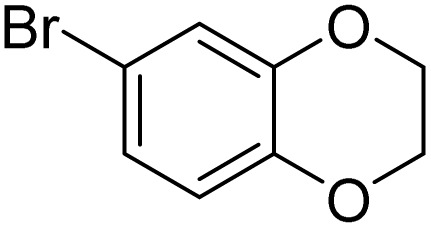	82
26	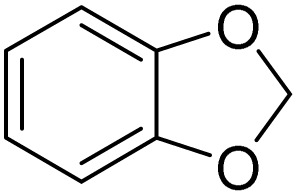	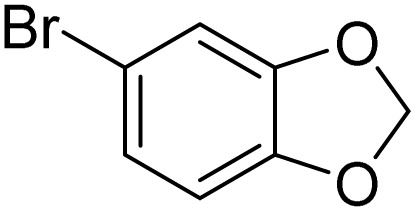	79
27	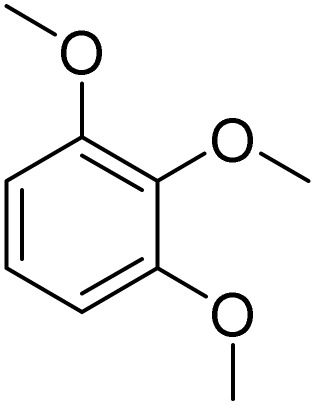	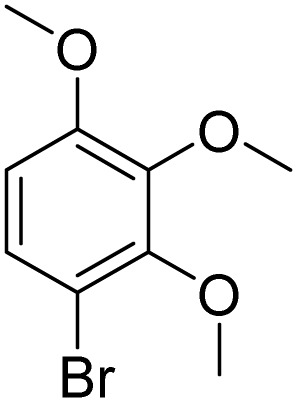	90^f^
28	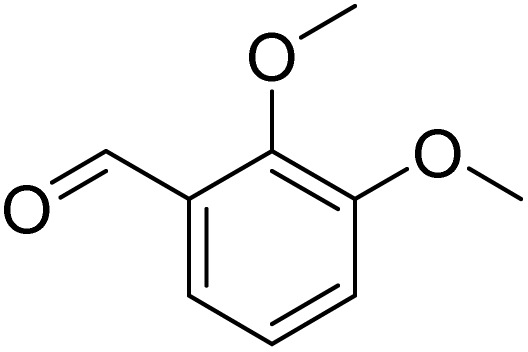	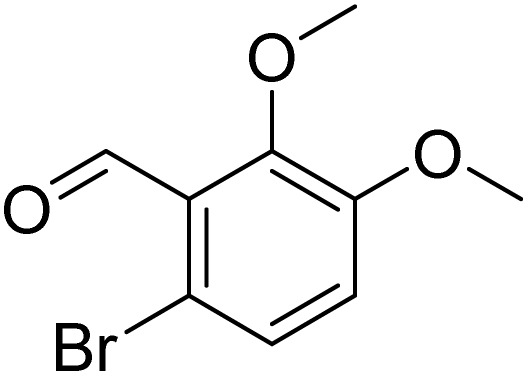	33/65^b^
29	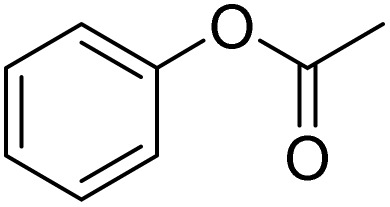	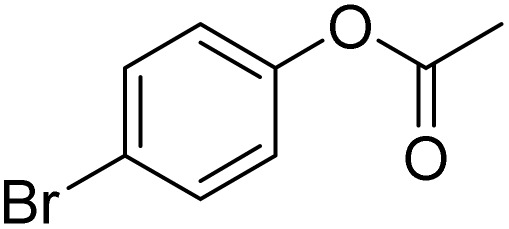	<1
30	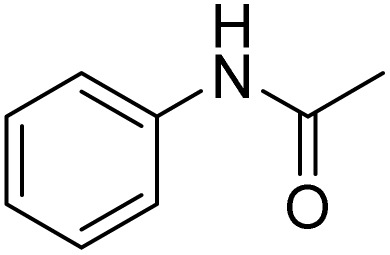	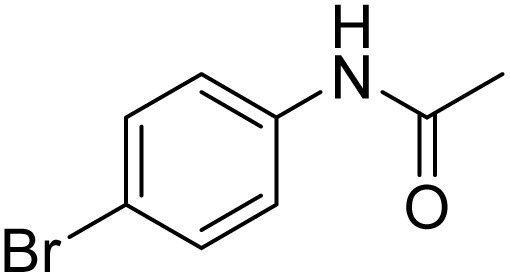	87

a Anisole (0.5 mmol), Fe(NO_3_)_3_·9H_2_O (0.625 mmol), KBr (0.625 mmol), rt, 2 h, GC yield.

b 60 °C.

c 4 h.

d Fe(NO_3_)_3_·9H_2_O (0.25 mmol).

e Fe(NO_3_)_3_·9H_2_O (0.25 mmol), KBr (0.6 mmol).

f Fe(NO_3_)_3_·9H_2_O (0.375 mmol), KBr (1.5 mmol).

Meanwhile, the strong electron-donating groups on anisole should be reacted under optimized conditions, that is, some bromination products or nitrification products were obtained under standard conditions (Table 2, entries 22–26). For 1,2-dimethoxybenzene, the side product was a nitrification product (15%). While the amount of Fe(NO_3_)_3_·9H_2_O was decreased to 0.25 mmol, the yield of desired product was increased to 94%, and the yield of nitrification product was decreased to 5% (Table 2, entry 22). For 1,3-dimethoxybenzene, the desired product was obtained in 99% yield at the ratio of Fe(NO_3_)_3_·9H_2_O (0.25 mmol)/KBr(0.6 mmol) (Table 2, entry 23). For 1,4-dimethoxybenzene, the main product was a nitrification product with 83% yield (Table 2, entry 24). In addition, for 1,2,3-trimethoxybenzene, the highly electron-rich substrate afforded the desired product in 90% yield with a Fe(NO_3_)_3_·9H_2_O/KBr ratio of 0.375 mmol/1.5 mmol. It should note that 2,3-dimethoxybenzaldehyde could offer 65% yield at a reaction temperature of 60 °C. Moreover, acetyl aniline and phenyl ester were also found to be compatible with this catalytic protocol with high yields (Table 2, entries 29 and 30). We also tested toluene, aniline and phenol, but unfortunately, the reaction was sluggish.

**Scheme 1:**
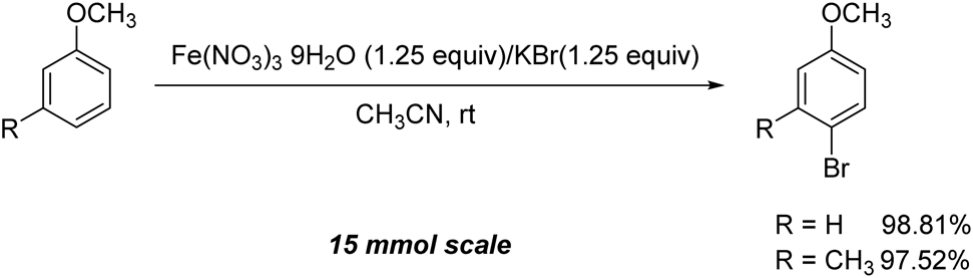
Scale-up of bromination by Fe(NO_3_)_3_·9H_2_O/KBr.

Furthermore, it is notable that the present bromination could be scaled up to gram level: excellent yields of the corresponding products were obtained in one batch from 15 mmol of anisole and 3-methylanisole by using Fe(NO_3_)_3_·9H_2_O/KBr as an additive for 22 h (Scheme 1).

Inspired by the protocol of the bromination of anisole, the scope of the reaction was extended to iodination with Fe(NO_3_)_3_·9H_2_O/NaI (Table S1†). The results are provided in Table 3, which indicate that this protocol is suitable for the iodination of anisole analogues by Fe(NO_3_)_3_·9H_2_O/NaI with a ratio of 2 equiv./2 equiv. for 5 h at room temperature. In addition, the results were similar to those of the bromination process.

**Table 3:** Iodination of various substrates^a^

Entry	Substrate	Product	Yield/%
1	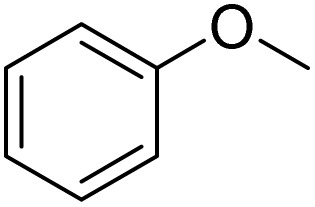	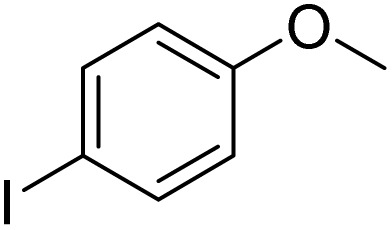	>99
2	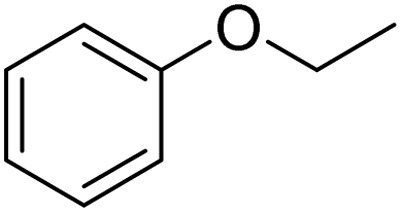	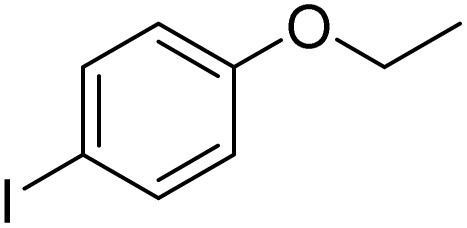	>99
3	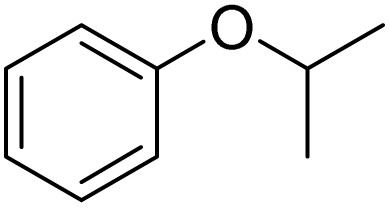	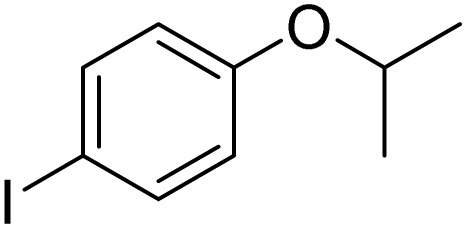	>99
4	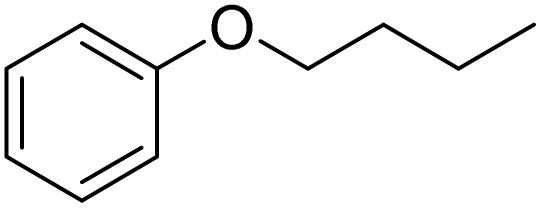	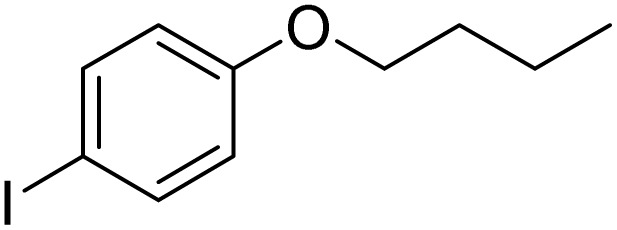	>99
5	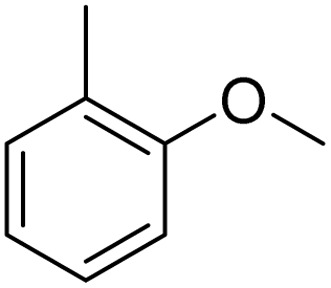	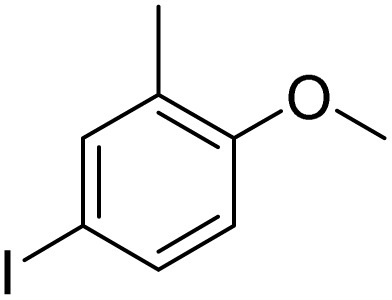	>99
6	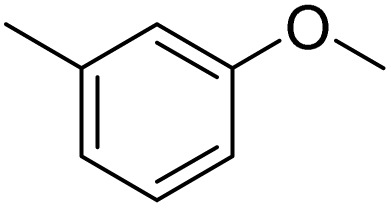	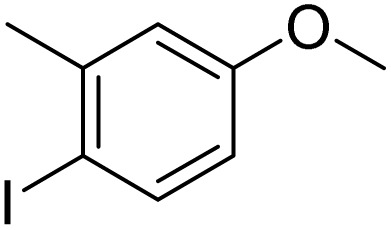	90
7	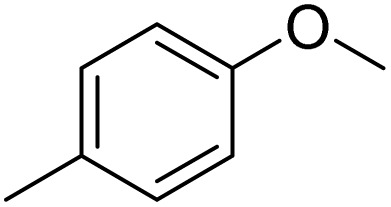	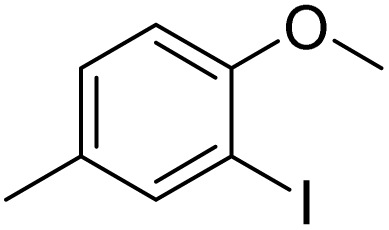	98
8	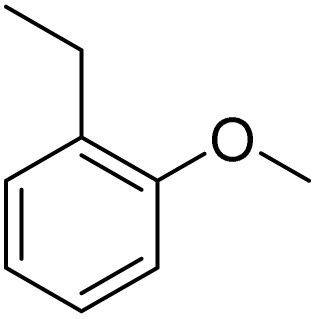	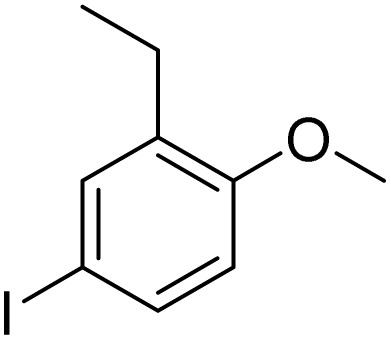	99
9	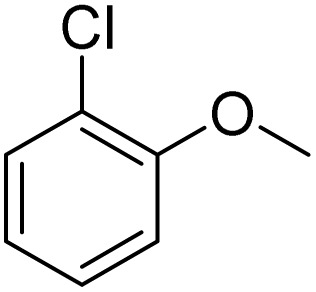	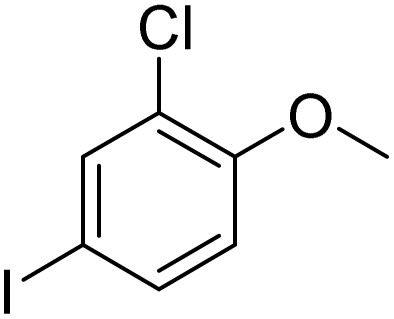	35^b^
10	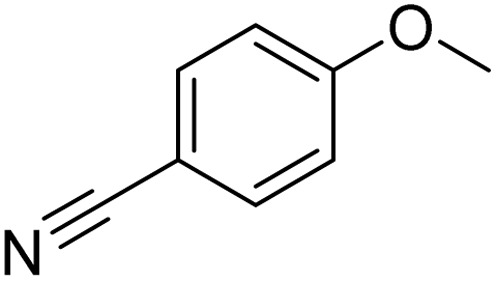	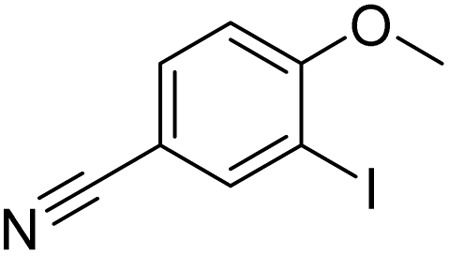	<1
11	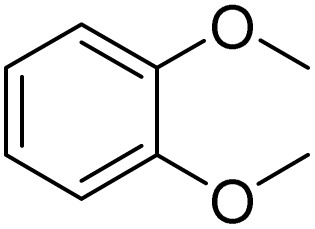	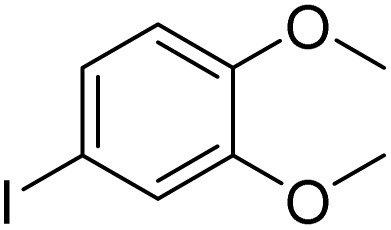	73
		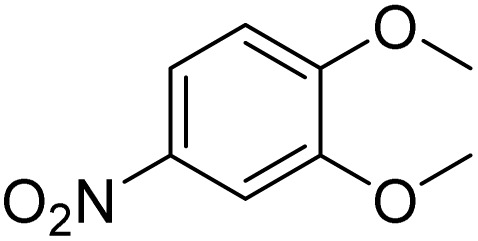	26
12	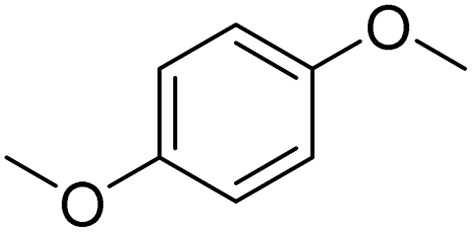	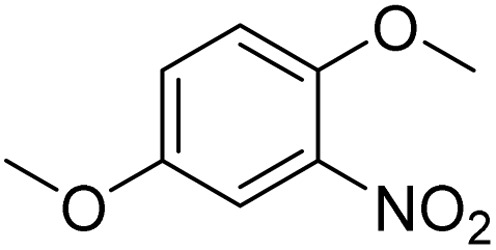	94
13	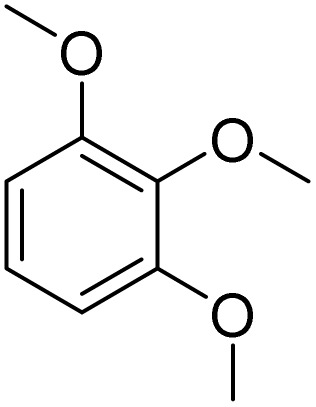	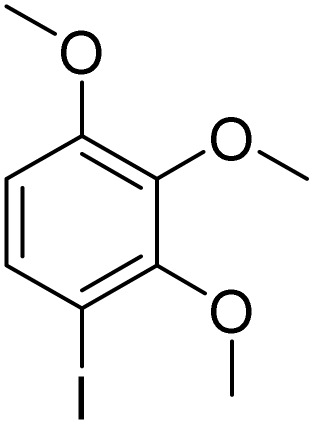	93
14	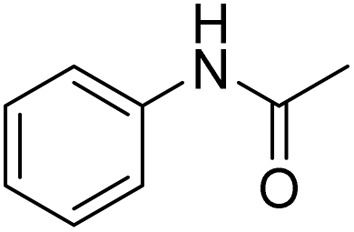	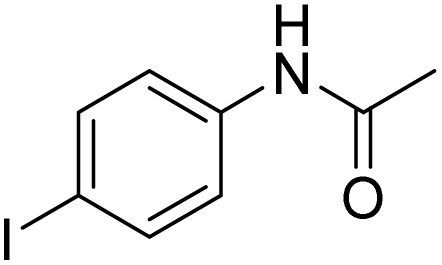	41/69^b^

a Anisole (0.5 mmol), Fe(NO_3_)_3_·9H_2_O (0.625 mmol), NaI (0.625 mmol), rt, 2 h, GC yield.

b 60 °C.

**Scheme 2:**
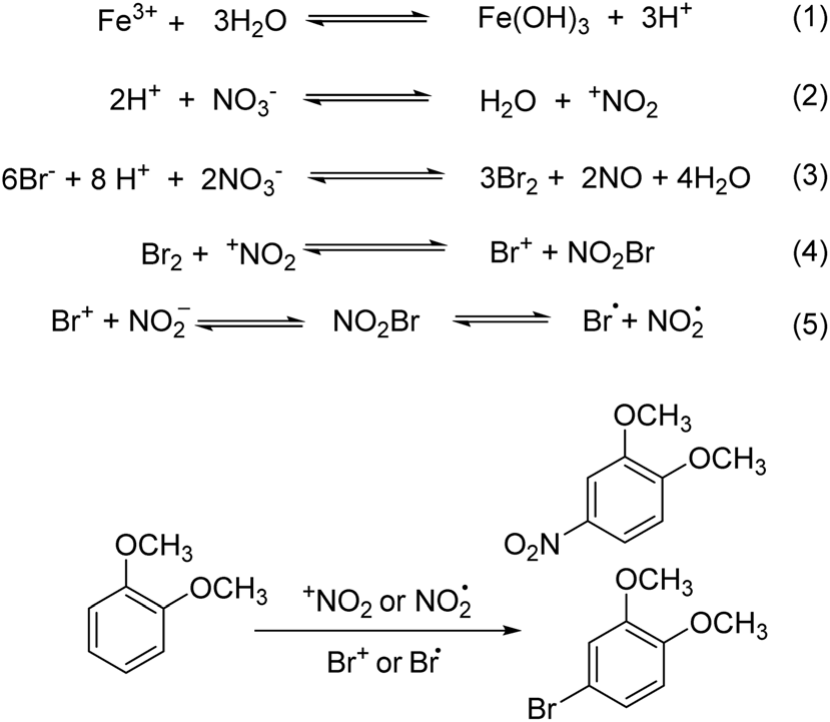
Possible mechanisms for bromination.

Moreover, possible mechanisms for the formation of the bromination are shown in Scheme 2. According to these results, that is, for 1,2-dimethoxybenzene, the side product was a nitro product (15%), and for 1,4-dimethoxybenzene, the main product was a nitrification product with 83% yield. Meanwhile, 2,2,6,6-tetramethylpiperidine-1-oxyl (TEMPO) and 2,6-di-*tert*-butyl-4-methylphenol (BHT) are the generally accepted radical scavengers in organic chemistry. It was successfully found that the addition of TEMPO and BHT could influence the nitrification process very significantly. For example, a low yield of 2 was obtained when TEMPO (2%, 0.5 equiv.) or BHT (7%, 0.25 equiv.) was added, and the bromination process was affected to a small extent under the reaction condition (Table 4).

**Table 4:** Quenching experiments for the bromination process

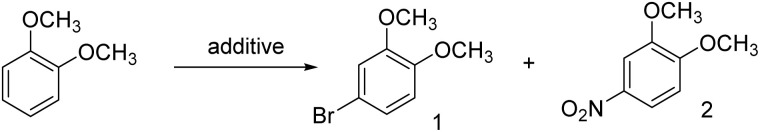			
Additive	Equiv.	Yield of 1 (%)	Yield of 2 (%)
TEMPO	0	72	16
TEMPO	0.5	69	2
TEMPO	0.25	60	13
BHT	0.25	72	7

From these results, we propose a similar ionic pathway for our bromination process since our results are comparable with some of those works, 25a–c (Scheme 2). First, Fe(NO_3_)_3_·9H_2_O was reacted with Fe(OH)_3_ and H^+^ (eqn (1)), and then NO_3_^−^ was converted into ^+^NO_2_ (eqn (2)). The bromide ion (Br^−^) from KBr would be reacted with ^−^NO_3_ and H^+^ to generate intermediate Br_2_ (eqn (3)). Then, unstable nitryl bromide (BrNO_2_) was formed and depending on the substrate nature, it could act as electrophilic and radical species (eqn (4) and (5)). It was presumed that the bromination process involves typical electrophilic bromination to a large extent.

## Conclusions

In conclusion, we have developed a simple, efficient and environmentally friendly methodology for the halogenation of anisole analogues using Fe(NO_3_)_3_·9H_2_O/KBr or NaI at room temperature. In this method, substrates with an electron-donating group afforded the corresponding product in good to excellent yields, while those with an electron-withdrawing group gave low to moderate yields. More importantly, this protocol is also applicable to gram-scale synthesis. It is hoped that this methodology will be very much useful in organic synthesis.

## Data availability

The authors confirm that the data supporting the findings of this study are available within the article and its ESI.†

## Author contributions

Conceptualization: Huanjun Xu and Yiying Li; methodology: Caicui Li, Yao Cheng and Fudan Pang; formal analysis: Xinmei Wang and Xiaodan Wang; investigation: Caicui Li, Xiushuo Yan, Zhengtao Huang and Yao Cheng; resources: Yiying Li, Huanjun Xu and Jinhui Wang; writing – original draft preparation: Xiaodan Wang and Huanjun Xu; writing – review and editing: Huanjun Xu and Jinhui Wang; supervision: Yiying Li and Huanjun Xu; project administration: Huanjun Xu; and funding acquisition: Yiying Li, Huanjun Xu and Jinhui Wang. All the authors have read and agreed to the published version of the manuscript.

## Conflicts of interest

There are no conflicts to declare.

## Supplementary Material

RA-015-D5RA00837A-s001
