# Supplementary material for: Halogenation of arenes using alkali metal halides/Fe(NO3)3·9H2O at room temperature
Source: RSC Adv. 2025 Mar 19;15(11):8523–8. doi: 10.1039/d5ra00837a (PMC11920861; doi:10.1039/d5ra00837a)
Supplement: RA-015-D5RA00837A-s001 [file RA-015-D5RA00837A-s001.pdf]

This journal is © The Royal Society of Chemistry 2025

Supporting Information

I. General experimental details

1.1. Materials

All of the materials were purchased from Beijing Innochem Company, and used as received.

1.2 Characterization

Some products were purified by flashchromatography on silica gel. anisole: analysis of crude reaction mixture was performed on a SHIMADZU 2030 GC System with a HPINNOWAX capillary column (30 m×0.25 mm×0.32 μm) and an FID detector. The following GC temperature program was used: 45°C is maintained for 2 minutes, rises to 280 °C at 15 °C/min, and hold for 5 minutes. Nitrogen was used as a carrier gas. The injector temperature was held at 250 °C. ^{1}H-NMR and ^{13}C-NMR spectra spectra were acquired on a 400 MHz JNM-ECZ400S/L1 instrument. Chemical shifts were reported in ppm relative to a peak of a residual protiated solvent ($CDCl_3$ or DMSO).

1.3 General procedures for typical procedure

Typical procedure: iron nitrate as catalyst,potassium bromide provides the bromine source,add the required amount of primary ether bottom (0.5 mmol),potassium bromide(0.625 mmol) and iron nitrate (0.625 mmol), acetonitrile (3 mL) to 10 mL reaction tube (open air), and complete the reaction within the setting time of room temperature. After the reaction, 2ml saturated sodium thiosulfate solution, 3ml saturated salt solution and 12ml dichloromethane were added for extraction.The desired product was purified by column chromatography on 200-300-mesh silica gel using ethyl acetate / petroleum ether as the elution agent. Some of the products are as follows: the combined substrate by GC / FID method, with dodecane as the internal standard for quantitative identification of the standard substrate.

iron nitrate as catalyst,sodium iodide provides the iodide source,add the required amount of primary ether bottom (0.5 mmol),sodium iodide(1 mmol) and iron nitrate (1 mmol), acetonitrile (3 mL) to 10 mL reaction tube (open air), and complete the reaction within the setting time of room temperature. After the reaction, 2ml saturated sodium thiosulfate solution, 3ml saturated salt solution and 12ml dichloromethane were added for extraction.The desired product was purified by column chromatography on 200-300-mesh silica gel using ethyl acetate / petroleum ether as the

elution agent. Some of the products are as follows: the combined substrate by GC / FID method, with dodecane as the internal standard for quantitative identification of the standard substrate.

II.Data of ^{1}H NMR and ^{13}C NMR of products

1-bromo-4-methoxybenzene(table 2-1)[1]: ^{1}H NMR (400 MHz, $CDCl_3$) δ 7.38 (d, J = 9.0 Hz, 2H), 6.78 (d, J = 9.0 Hz, 2H), 3.78 (s, 3H).^{13}C{^{1}H} NMR (101 MHz, $CDCl_3$) δ 158.8, 132.4, 115.9, 112.9, 77.5, 77.2, 76.8, 55.6.

1-bromo-4-ethoxybenzene(table 2-2)[2]: ^{1}H NMR (400 MHz, $CDCl_3$) δ 7.48 – 7.30 (m, 2H), 6.91 – 6.65 (m, 2H), 3.99 (q, J = 7.0 Hz, 2H), 1.40 (t, J = 7.0 Hz, 3H).^{13}C{^{1}H} NMR (101 MHz, $CDCl_3$) δ 158.2, 132.3, 116.4, 112.6, 77.5, 77.2, 76.8, 63.8, 14.9.

1-bromo-4-isopropoxybenzene(table 2-3)[3]: ^{1}H NMR (400 MHz, $CDCl_3$) δ 7.41 – 7.31 (m, 2H), 6.81 – 6.71 (m, 2H), 4.49 (p, J = 6.0 Hz, 1H), 1.32 (d, J = 6.1 Hz, 6H).^{13}C{^{1}H}NMR (101 MHz, $CDCl_3$) δ 157.1, 132.4, 117.8, 112.7, 77.5, 77.2, 76.8, 70.4, 22.1.

1-bromo-4-(tert-butoxy)benzene(table 2-4)[4]: ^{1}H NMR (400 MHz, $CDCl_3$)δ 7.34 – 7.22 (m, 2H), 6.85 – 6.72 (m, 2H), 1.24 (s, 9H).^{13}C{^{1}H}NMR (101 MHz, $CDCl_3$) δ 158.4, 132.3, 116.4, 112.7, 68.1, 31.4, 19.3, 14.0.

1-bromo-4-butoxybenzene(table 2-5)[5]: ^{1}H NMR (400 MHz, $CDCl_3$) δ 7.42 – 7.29 (m, 2H), 6.84 – 6.72 (m, 2H), 3.92 (t, J = 6.5 Hz, 2H), 1.83 – 1.67 (m, 2H), 1.54 – 1.41 (m, 2H), 0.97 (t, J = 7.4 Hz, 3H).^{13}C{^{1}H} NMR (101 MHz, $CDCl_3$) δ 158.4, 132.3, 116.4, 112.7, 68.1, 31.4, 19.3, 14.0.

1-(benzyloxy)-4-bromobenzene(table 2-6)[6]: ^{1}H NMR (400 MHz, $CDCl_3$) δ 7.40 (m, 8H), 6.87 (m, 2H), 5.04 (t, J = 4.8 Hz, 2H).^{13}C{^{1}H}NMR (101 MHz, $CDCl_3$) δ 158.0, 136.7, 132.4, 128.8, 128.2, 127.6, 116.8, 113.3, 77.5, 77.2, 76.8, 70.3.

1-bromo-2-methoxynaphthalene(table 2-7)[1]: ^{1}H NMR (400 MHz, $CDCl_3$) δ 8.23 (dt, J = 8.7, 0.9 Hz, 1H), 7.85 – 7.74 (m, 2H), 7.57 (ddd, J = 8.4, 6.8, 1.3 Hz, 1H), 7.40 (ddd, J = 8.0, 6.8, 1.1 Hz, 1H), 7.27 (d, J = 9.0 Hz, 1H), 4.03 (s, 3H).^{13}C{^{1}H} NMR (101 MHz, $CDCl_3$) δ 153.9, 133.3, 129.9, 129.1, 128.2, 127.9, 126.2, 124.4, 113.7, 108.8, 77.5, 77.2, 76.8, 57.2.

1-bromo-4-methoxynaphthalene(table 2-8)[1]: ^{1}H NMR (400 MHz, $CDCl_3$) δ 8.27 (ddd, J = 8.4, 1.4, 0.7 Hz, 1H), 8.16 (ddt, J = 8.5, 1.4, 0.7 Hz, 1H), 7.65 (d, J = 8.2 Hz, 1H), 7.60 (ddd, J = 8.4, 6.8, 1.3 Hz, 1H), 7.52 (ddd, J = 8.2, 6.8, 1.3 Hz, 1H), 6.67 (d, J = 8.2 Hz, 1H), 3.98 (s, 3H).

$^{13}C\{^{1}H\}$ NMR (101 MHz, $CDCl_3$) δ 155.4, 132.5, 129.6, 127.9, 127.0, 126.9, 126.1, 122.6, 113.4, 104.6, 77.5, 77.2, 76.8, 55.8.

3-bromo-4-methoxy-1,1'-biphenyl(table 2-9)[7]: ^{1}H NMR (400 MHz, $CDCl_3$) δ 7.80 (d, J = 2.3 Hz, 1H), 7.56 – 7.49 (m, 3H), 7.43 (ddd, J = 7.8, 6.9, 1.2 Hz, 2H), 7.37 – 7.30 (m, 1H), 6.97 (d, J = 8.5 Hz, 1H), 3.94 (s, 3H). $^{13}C\{^{1}H\}$ NMR (101 MHz, $CDCl_3$) δ 155.4, 139.6, 135.3, 132.1, 129.0, 127.4, 127.2, 126.9, 112.2, 112.2, 77.5, 77.2, 76.8, 56.5.

4-bromo-1-methoxy-2-methylbenzene(table 2-10)[1]: ^{1}H NMR (400 MHz, $CDCl_3$) δ 7.30 – 7.18 (m, 2H), 6.72 – 6.65 (m, 1H), 3.80 (s, 3H), 2.19 (s, 3H). $^{13}C\{^{1}H\}$ NMR (101 MHz, $CDCl_3$) δ 157.0, 133.3 129.5, 129.1, 112.5, 111.6, 77.5, 77.2, 76.8, 55.6, 16.2.

1-bromo-4-methoxy-2-methylbenzene(table 2-11)[1]: ^{1}H NMR (400 MHz, $CDCl_3$)δ 7.40 (d, J = 8.8 Hz, 1H), 6.79 (dd, J = 3.1, 0.8 Hz, 1H), 6.61 (ddd, J = 8.8, 3.1, 0.6 Hz, 1H), 3.77 (s, 3H), 2.36 (s, 3H).$^{13}C\{^{1}H\}$ NMR (101 MHz, $CDCl_3$)δ 158.9, 139.0, 132.9, 116.6, 115.56, 113.1, 77.5, 77.4, 77.2, 76.8, 55.5, 23.3.

2-bromo-1-methoxy-4-methylbenzene(table 2-12)[1]: ^{1}H NMR (400 MHz, $CDCl_3$)δ 7.36 (dd, J = 2.1, 0.8 Hz, 1H), 7.06 (ddt, J = 8.3, 2.2, 0.8 Hz, 1H), 6.79 (d, J = 8.3 Hz, 1H), 3.86 (s, 3H), 2.27 (t, J = 0.7 Hz, 3H).$^{13}C\{^{1}H\}$NMR (101 MHz, $CDCl_3$)δ 153.9, 133.9, 131.6, 129.0, 112.0, 111.4, 77.5, 77.2, 76.84, 56.4, 20.3.

4-bromo-2-ethyl-1-methoxybenzene(table 2-13)[8]: ^{1}H NMR (400 MHz, $CDCl_3$)δ 7.29 – 7.21 (m, 2H), 6.70 (ddd, J = 8.0, 4.8, 3.6 Hz, 1H), 3.80 (q, J = 3.5 Hz, 3H), 2.59 (tq, J = 7.5, 3.7 Hz, 2H), 1.17 (tt, J = 7.5, 3.8 Hz, 3H).$^{13}C\{^{1}H\}$ NMR (101 MHz, $CDCl_3$)δ 156.6, 135.0, 131.7, 129.4, 112.7, 111.9, 55.6, 23.1, 13.9.

5-bromo-2-methoxybenzaldehyde(table 2-14)[9]: ^{1}H NMR (400 MHz, $CDCl_3$) δ 10.38 (s, 1H), 7.91 (d, J = 2.6 Hz, 1H), 7.63 (dd, J = 8.9, 2.6 Hz, 1H), 6.89 (d, J = 8.9 Hz, 1H), 3.92 (s, 3H). $^{13}C\{^{1}H\}$ NMR (101 MHz, $CDCl_3$) δ 188.6, 160.9, 138.4, 131.2, 126.2, 113.8, 113.6, 77.5, 77.2, 76.8, 56.1.

methyl 5-bromo-2-methoxybenzoate(table 2-15)[10]: ^{1}H NMR (400 MHz, $CDCl_3$) δ 7.90 (d, J = 2.6 Hz, 1H), 7.54 (dd, J = 8.9, 2.6 Hz, 1H), 6.86 (d, J = 8.9 Hz, 1H), 3.88 (s, 6H). $^{13}C\{^{1}H\}$ NMR (101 MHz, $CDCl_3$) δ 165.4, 158.4, 136.2, 134.4, 121.8, 114.0, 112.3, 77.5, 77.2, 76.8, 56.4, 52.4.

1-(5-bromo-2-methoxyphenyl)ethanone(table 2-16-1)[10]: ^{1}H NMR (400 MHz, $CDCl_3$) δ 7.82 (d, J = 2.6 Hz, 1H), 7.53 (dd, J = 8.8, 2.6 Hz, 1H), 6.85 (d, J = 8.8 Hz, 1H), 3.89 (s, 3H), 2.59 (s, 3H).

$^{13}C\{^1H\}$ NMR (101 MHz, $CDCl_3$) δ 198.4, 158.1, 136.2, 133.1, 129.7, 113.7, 113.2, 77.5, 77.2, 76.8, 55.9, 31.8.

4-bromo-2-chloro-1-methoxybenzene(table 2-17)[10]: ^1H NMR (400 MHz, $CDCl_3$) δ 7.50 (d, J = 2.4 Hz, 1H), 7.33 (dd, J = 8.8, 2.4 Hz, 1H), 6.80 (d, J = 8.8 Hz, 1H), 3.88 (s, 3H). $^{13}C\{^1H\}$ NMR (101 MHz, $CDCl_3$) δ 154.5, 132.8, 130.7, 123.7, 113.4, 112.6, 77.5, 77.2, 76.8, 56.4.

1-bromo-2-chloro-4-methoxybenzene(table 2-18)[10]: ^1H NMR (400 MHz, $CDCl_3$) δ 7.45 (d, J = 8.9 Hz, 1H), 6.99 (d, J = 2.9 Hz, 1H), 6.68 (dd, J = 8.9, 2.9 Hz, 1H), 3.77 (s, 3H). $^{13}C\{^1H\}$ NMR (101 MHz, $CDCl_3$) δ 159.5, 135.0, 134.0, 115.9, 114.6, 1123.0, 77.5, 77.2, 76.8, 55.8.

2-bromo-4-chloro-1-methoxybenzene(table 2-19)[1]: ^1H NMR (400 MHz, $CDCl_3$) δ 7.68 – 7.38 (m, 1H), 7.33 – 7.09 (m, 1H), 6.92 – 6.67 (m, 1H), 3.85 (s, 3H). $^{13}C\{^1H\}$ NMR (101 MHz, $CDCl_3$) δ 154.9, 132.9, 128.4, 126.1, 112.6, 112.2, 77.4, 77.1, 76.8, 56.6.

4-bromo-1,2-dimethoxybenzene(table 2-22-1)[1]: ^1H NMR (400 MHz, $CDCl_3$) δ 7.03 (dd, J = 8.5, 2.3 Hz, 1H), 6.98 (d, J = 2.3 Hz, 1H), 6.73 (d, J = 8.5 Hz, 1H), 3.86 (s, 3H), 3.85 (s, 3H). $^{13}C\{^1H\}$ NMR (101 MHz, $CDCl_3$) δ 149.8, 148.4, 123.4, 114.8, 112.8, 112.5, 77.4, 77.1, 76.8, 56.2, 56.1.

1,2-dimethoxy-4-nitrobenzene(table 2-22-2 and table 3-18-2)[1]: ^1H NMR (400 MHz, $CDCl_3$) δ 7.91 (dd, J = 8.8, 2.6 Hz, 1H), 7.73 (d, J = 2.6 Hz, 1H), 6.91 (d, J = 8.8 Hz, 1H), 3.97 (s, 3H), 3.95 (s, 3H). $^{13}C\{^1H\}$ NMR (101 MHz, $CDCl_3$) δ 154.6, 149.0, 141.6, 117.9, 109.9, 106.5, 77.5, 77.2, 76.8, 56.6, 56.4.

1-bromo-2,4-dimethoxybenzene(table 2-23)[1]: ^1H NMR (400 MHz, $CDCl_3$) δ 7.41 (d, J = 8.7 Hz, 1H), 6.49 (d, J = 2.7 Hz, 1H), 6.40 (dd, J = 8.7, 2.7 Hz, 1H), 3.87 (s, 3H), 3.80 (s, 3H). $^{13}C\{^1H\}$ NMR (101 MHz, $CDCl_3$) δ 160.4, 156.7, 133.3, 106.0, 102.6, 100.1, 56.3, 55.7.

1,4-dimethoxy-2-nitrobenzene(table 2-24 and table 3-20)[11]: ^1H NMR (400 MHz, $CDCl_3$) δ 7.39 (d, J = 3.1 Hz, 1H), 7.11 (dd, J = 9.2, 3.1 Hz, 1H), 7.03 (d, J = 9.2 Hz, 1H), 3.91 (s, 3H), 3.81 (s, 3H). $^{13}C\{^1H\}$ NMR (101 MHz, $CDCl_3$) δ 153.0, 147.5, 139.7, 121.1, 115.3, 110.1, 77.5, 77.2, 76.8, 57.2, 56.2

6-bromo-2,3-dihydrobenzo[b][1,4]dioxine(table 2-25)[9]: ^1H NMR(400 MHz, $CDCl_3$) δ 7.01 (d, J = 2.3 Hz, 1H), 6.93 (dd, J = 8.6, 2.4 Hz, 1H), 6.73 (d, J = 8.6 Hz, 1H), 4.24 (s, 4H). $^{13}C\{^1H\}$ NMR (101 MHz, $CDCl_3$)δ 144.5, 143.0, 124.4, 120.4, 118.7, 112.9, 64.4, 64.3.

5-bromobenzo[d][1,3]dioxole(table 2-26)[12]: ^{1}H NMR(400 MHz, $CDCl_3$) δ 7.01 – 6.87 (m, 2H), 6.69 (dt, J = 8.1, 0.8 Hz, 1H), 5.97 (d, J = 0.7 Hz, 2H). $^{13}C\{^1H\}$ NMR (101 MHz, $CDCl_3$) δ 148.7, 147.1, 124.5, 113.2, 112.4, 109.7, 101.7.

1-bromo-2,3,4-trimethoxybenzene(table 2-27)[13]: ^{1}H NMR(400 MHz, $CDCl_3$) δ 7.20 (d, J = 8.9 Hz, 1H), 6.58 (d, J = 8.9 Hz, 1H), 3.90 (s, 3H), 3.88 (s, 3H), 3.84 (s, 3H). $^{13}C\{^1H\}$ NMR (101 MHz, $CDCl_3$) δ 153.5, 151.1, 143.7, 126.9, 108.7, 108.5, 77.5, 77.2, 76.8, 61.2, 61.2, 56.3.

6-bromo-2,3-dimethoxybenzaldehyde(table 2-28)[14]: ^{1}H NMR(400 MHz, $CDCl_3$) δ 10.33 (d, J = 2.3 Hz, 1H), 7.34 (dd, J = 8.8, 2.3 Hz, 1H), 6.96 (dd, J = 8.9, 2.2 Hz, 1H), 3.92 (d, J = 2.3 Hz, 3H), 3.88 (d, J = 2.2 Hz, 3H). $^{13}C\{^1H\}$ NMR (101 MHz, $CDCl_3$) δ 190.6, 152.9, 152.2, 129.5, 128.7, 117.6, 112.9, 77.5, 77.1, 76.8, 62.5, 56.3, 31.5, 30.3, 29.8.

N-(4-bromophenyl)acetamide(table 2-30)[1]: ^{1}H NMR(400 MHz, $CDCl_3$) δ 7.41 (d, J = 1.9 Hz, 5H), 2.17 (s, 3H). $^{13}C\{^1H\}$ NMR (101 MHz, $CDCl_3$) δ 168.5, 137.1, 132.1, 121.5, 117.0, 77.5, 77.4, 77.2, 76.8, 24.7.

1-iodo-4-methoxybenzene (table 3-1)[15]: ^{1}H NMR(400 MHz, $CDCl_3$) δ 7.56 (d, J = 9.0 Hz, 1H), 6.68 (d, J = 9.0 Hz, 1H), 3.78 (s, 3H). $^{13}C\{^1H\}$ NMR (101 MHz, $CDCl_3$) δ 159.6, 138.3, 116.5, 82.8, 77.5 77.4, 77.2, 76.8, 55.5.

1-ethoxy-4-iodobenzene (table 3-2)[15]: ^{1}H NMR(400 MHz, $CDCl_3$) δ 7.54 (d, J = 8.9 Hz, 2H), 6.67 (d, J = 8.9 Hz, 2H), 3.99 (q, J = 7.0 Hz, 2H), 1.40 (t, J = 7.0 Hz, 3H). $^{13}C\{^1H\}$ NMR (101 MHz, $CDCl_3$) δ 158.9, 138.3, 117.0, 82.6, 77.5, 77.2, 76.8, 63.7, 14.9.

1-iodo-4-isopropoxybenzene (table 3-3)[16]: ^{1}H NMR(400 MHz, $CDCl_3$) δ7.53 (d, J = 8.9 Hz, 2H), 6.66 (d, J = 8.9 Hz, 2H), 4.49 (hept, J = 6.1 Hz, 1H), 1.32 (d, J = 6.1 Hz, 6H). $^{13}C\{^1H\}$ NMR (101 MHz, $CDCl_3$) δ 157.9, 138.4, 118.4, 82.5, 77.5, 77.2, 76.8, 70.2, 22.0.

1-(tert-butoxy)-4-iodobenzene (table 3-4)[15]: ^{1}H NMR(400 MHz, $CDCl_3$) δ 7.54 (d, J = 8.9 Hz, 2H), 6.67 (d, J = 8.9 Hz, 2H), 3.99 (q, J = 7.0 Hz, 2H), 1.40 (t, J = 7.0 Hz, 3H). $^{13}C\{^1H\}$ NMR (101 MHz, $CDCl_3$) δ 158.9, 138.3, 117.0, 82.6, 77.5, 77.2, 76.8, 63.7, 14.9.

1-butoxy-4-iodobenzene (table 3-5)[17]: ^{1}H NMR(400 MHz, $CDCl_3$)δ7.53 (d, J = 8.9 Hz, 2H), 6.66 (d, J = 8.9 Hz, 2H), 4.49 (hept, J = 6.1 Hz, 1H), 1.32 (d, J = 6.1 Hz, 6H). $^{13}C\{^1H\}$ NMR (101 MHz, $CDCl_3$) δ 159.1, 138.3, 117.1, 82.5, 67.9, 31.3, 19.3, 14.0.

4-iodo-1-methoxy-2-methylbenzene (table 3-6)[9]: ^{1}H NMR(400 MHz, $CDCl_3$) δ 7.51 – 7.39 (m, 2H), 6.59 (dd, J = 8.4, 2.6 Hz, 1H), 3.80 (s, 3H), 2.17 (s, 3H). ^{13}C{^{1}H} NMR (101 MHz, $CDCl_3$) δ 157.8, 139.1, 135.6, 129.6, 112.3, 82.6, 77.5, 77.2, 76.9, 55.5, 16.0.

1-iodo-4-methoxy-2-methylbenzene (table 3-7)[18]: ^{1}H NMR(400 MHz, $CDCl_3$) δ 7.66 (d, J = 8.7 Hz, 1H), 6.82 (dd, J = 2.9, 0.8 Hz, 1H), 6.48 (dd, J = 8.7, 3.1 Hz, 1H), 3.77 (s, 3H), 2.40 (s, 3H). ^{13}C{^{1}H} NMR (101 MHz, $CDCl_3$) δ 160.0, 142.5, 139.5, 116.0, 113.50, 89.8, 77.5, 77.2, 76.8, 55.4, 28.4.

2-iodo-1-methoxy-4-methylbenzene (table 3-8)[15]: ^{1}H NMR(400 MHz, $CDCl_3$) δ 7.60 (d, J = 2.1 Hz, 1H), 7.14 – 7.05 (m, 1H), 6.72 (d, J = 8.3 Hz, 1H), 3.85 (s, 3H), 2.26 (s, 3H). ^{13}C{^{1}H} NMR (101 MHz, $CDCl_3$) δ 160.0, 142.5, 139.5, 116.0, 113.5, 89.8, 77.5, 77.2, 76.8, 55.4, 28.4.

2-ethyl-4-iodo-1-methoxybenzene (table 3-9)[15]: ^{1}H NMR(400 MHz, $CDCl_3$) δ 7.57 – 7.35 (m, 2H), 6.59 (dd, J = 8.5, 3.9 Hz, 1H), 3.86 – 3.66 (m, 3H), 2.66 – 2.50 (m, 2H), 1.28 – 1.10 (m, 3H). ^{13}C{^{1}H} NMR (101 MHz, $CDCl_3$) δ 157.4, 137.6, 135.6, 135.6, 112.6, 83.0, 77.5, 77.2, 76.8, 55.5, 23.1, 14.0.

2-chloro-4-iodo-1-methoxybenzene (table 3-10)[19]: ^{1}H NMR(400 MHz, $CDCl_3$) δ 7.57 – 7.35 (m, 2H), 6.59 (dd, J = 8.5, 3.9 Hz, 1H), 3.86 – 3.66 (m, 3H), 2.66 – 2.50 (m, 2H), 1.28 – 1.10 (m, 3H). ^{13}C{^{1}H} NMR (101 MHz, $CDCl_3$) δ 157.4, 137.6, 135.6, 135.6, 112.6, 83.0, 77.5, 77.2, 76.8, 55.5, 23.1, 14.0.

4-iodo-1,2-dimethoxybenzene (table 3-18-1)[15]: ^{1}H NMR(400 MHz, $CDCl_3$) δ 7.23 (dd, J = 8.4, 2.0 Hz, 1H), 7.12 (d, J = 2.0 Hz, 1H), 6.62 (d, J = 8.4 Hz, 1H), 3.86 (s, 3H), 3.85 (s, 3H). ^{13}C{^{1}H}NMR (101 MHz, $CDCl_3$) δ 150.0, 149.3, 129.9, 120.5, 113.3, 82.5, 77.5, 77.4, 77.2, 76.8, 56.2, 56.1.

1,5-diiodo-2,4-dimethoxybenzene (table 3-19)[15]: ^{1}H NMR(400 MHz, $CDCl_3$) δ 8.03 (s, 1H), 6.37 (s, 1H), 3.89 (s, 6H). ^{13}C{^{1}H} NMR (101 MHz, $CDCl_3$) δ 159.8, 147.0, 95.9, 77.5, 77.2, 76.8, 75.6, 56.7.

2,4-diiodo-1,3,5-trimethoxybenzene (table 3-21)[20]: ^{1}H NMR(400 MHz, $CDCl_3$) δ 6.24 (hept, J = 3.5, 2.9 Hz, 1H), 3.95 – 3.86 (m, 6H), 3.85 (tt, J = 4.3, 2.4 Hz, 3H). ^{13}C{^{1}H} NMR (101 MHz, $CDCl_3$) δ 160.6, 160.5, 92.0, 77.5, 77.2, 76.8, 72.7, 60.7, 56.9.

1-iodo-2,3,4-trimethoxybenzene (table 3-22)[21]: ^{1}H NMR(400 MHz, $CDCl_3$) δ 7.42 (d, *J* = 9.0 Hz, 1H), 6.50 (d, *J* = 8.9 Hz, 1H), 3.87 (s, 6H), 3.85 (s, 3H). ^{13}C{^{1}H} NMR (101 MHz, $CDCl_3$) δ 154.6, 153.5, 142.9, 132.7, 109.9, 81.5, 61.2, 61.0, 56.3.

***N*-(4-iodophenyl)acetamide (table 3-24)[15]:** ^{1}H NMR(400 MHz, $CDCl_3$) δ 7.62 – 7.56 (m, 2H), 7.31 (s, 1H), 7.29 – 7.23 (m, 2H), 2.15 (s, 3H). ^{13}C{^{1}H} NMR (101 MHz, $CDCl_3$) δ 168.5, 138.1, 137.8, 121.8, 87.6, 77.5, 77.2, 76.8, 24.8.

Reference:

1. Chen P H, Hsu S J, Hou D R. Nitrosonium Ion Catalyzed Oxidative Bromination of Arenes[J]. Advanced Synthesis & Catalysis, 2024, 366(7): 1575-1582.

2. Sharma P K, Babbar A, Mallick D, et al. Constructing 1-Ethoxyphenanthro [9, 10-e] acephenanthrylene for the Synthesis of a Polyaromatic Hydrocarbon Containing a Formal Azulene Unit[J]. The Journal of Organic Chemistry, 2023, 88(9): 5473-5482.

3. Xiong X, Tan F, Yeung Y Y. Zwitterionic-salt-catalyzed site-selective monobromination of arenes[J]. Organic letters, 2017, 19(16): 4243-4246.

4. Bérubé C, Guay L D, Fraser T, et al. Convenient route to Fmoc-homotyrosine via metallaphotoredox catalysis and its use in the total synthesis of anabaenopeptin cyclic peptides[J]. Organic & Biomolecular Chemistry, 2023, 21(45): 9011-9020.

5. Dahiya A, Gevondian A G, Schoenebeck F. Orthogonal C–O Bond Construction with Organogermanes[J]. Journal of the American Chemical Society, 2023, 145(14): 7729-7735.

6. Ganguly A, Chandrasekaran R, Balamurugan B S S, et al. Application of Solid Me3SiZnI for the Synthesis of Aryl and Alkyl Trimethylsilanes[J]. Advanced Synthesis & Catalysis, 2024, 366(6): 1442-1447.

7. Schmidt B, Berger R. A Deacetylation–Diazotation–Coupling Sequence: Palladium-Catalyzed C-C Bond Formation with Acetanilides as Formal Leaving Groups[J]. Advanced Synthesis & Catalysis, 2013, 355(2-3): 463-476.

8. Barber V, Mielke T, Cartwright J, et al. Unspecific peroxygenase (UPO) can be tuned for oxygenation or halogenation activity by controlling the reaction pH[J]. Chemistry–A European Journal, 2024, 30(40): e202401706.

9. Mondal H, Patra S, Saha S, et al. Late-Stage Halogenation of Peptides, Drugs and (Hetero) aromatic Compounds with a Nucleophilic Hydrazide Catalyst[J]. Angewandte Chemie International Edition, 2023, 62(51): e202312597.

10. Pramanick P K, Hou Z L, Yao B. Mechanistic study on iodine-catalyzed aromatic bromination of aryl ethers by N-bromosuccinimide[J]. Tetrahedron, 2017, 73(50): 7105-7114.

11. Zheng Y, Hu Q Q, Huang Q, et al. Late-Stage C–H Nitration of Unactivated Arenes by Fe (NO3) 3· 9H2O in Hexafluoroisopropanol[J]. Organic Letters, 2024, 26(15): 3316-3320.

12. Karlsen M, Liu H L, Berg T, et al. Synthesis of [13C6]-labelled phenethylamine derivatives for drug quantification in biological samples[J]. Journal of Labelled Compounds and Radiopharmaceuticals, 2014, 57(5): 378-387.

13. Ren Y L, Wang B, Tian X Z, et al. Aerobic oxidative bromination of arenes using an ionic liquid as both the catalyst and the solvent[J]. Tetrahedron letters, 2015, 56(46): 6452-6455.

14. Borges I D, Navarrete A, Aguirre G, et al. Synthesis and molecular modeling study of two bromo-dimethoxybenzaldehydes[J]. Journal of the Brazilian Chemical Society, 2022, 33(09): 1069-1085.

15. Narobe R, Düsel S J S, Iskra J, et al. Photocatalytic Oxidative Iodination of Electron-Rich Arenes[J]. Advanced Synthesis & Catalysis, 2019, 361(17): 3998-4004.

16. Bodzioch A, Obijalska E, Jakubowski R, et al. Electronic and Magnetic Interactions in 6-Oxoverdazyl Diradicals: Connection through N (1) vs C (3) Revisited[J]. The Journal of Organic Chemistry, 2024, 89(9): 6306-6321.

17. Xie T, Li Y, Zhang M, et al. Aggregation-induced emission activity of sensor TBM-C1 hybrid of methoxy-triphenylamine (OMe-TPA) and dicyanovinyl for cyanide detection in aqueous THF: Mechanistic insights and potential applications[J]. Spectrochimica Acta Part A: Molecular and Biomolecular Spectroscopy, 2024, 312: 124058.

18. Dherbassy Q, Djukic J P, Wencel-Delord J, et al. Two stereoinduction events in one C− H activation step: A route towards terphenyl ligands with two atropisomeric axes[J]. Angewandte Chemie, 2018, 130(17): 4758-4762.

19. Webster S, O'Rourke K M, Fletcher C, et al. Rapid Iododeboronation with and without Gold Catalysis: Application to Radiolabelling of Arenes[J]. Chemistry–A European Journal, 2018, 24(4): 937-943.

20. Zhang J, Li S, Deng G J, et al. Metal-Free, Oxidant-Free, and Controllable Graphene Oxide Catalyzed Direct Iodination of Arenes and Ketones[J]. ChemCatChem, 2018, 10(2): 376-380.

21. Wu Z, Wei F, Wan B, et al. Pd-Catalyzed ipso, meta-Dimethylation of ortho-Substituted Iodoarenes via a Base-Controlled C–H Activation Cascade with Dimethyl Carbonate as the Methyl Source[J]. Journal of the American Chemical Society, 2021, 143(12): 4524-4530.

III. NMR spectra of the products

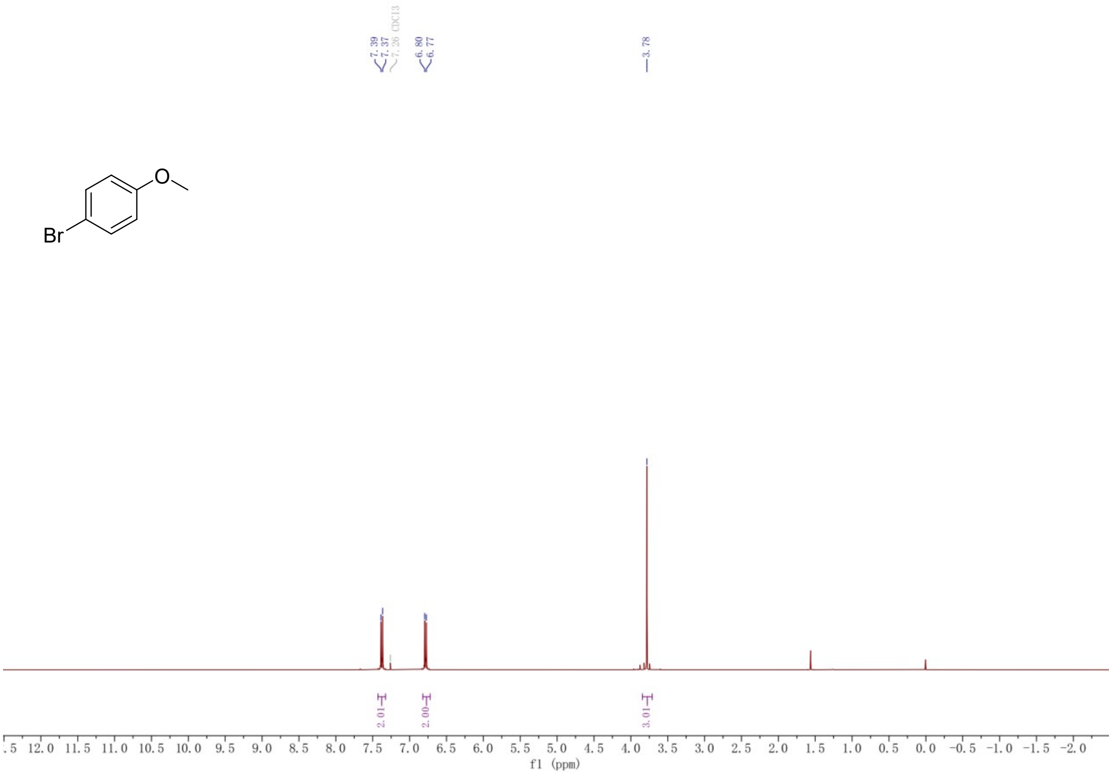

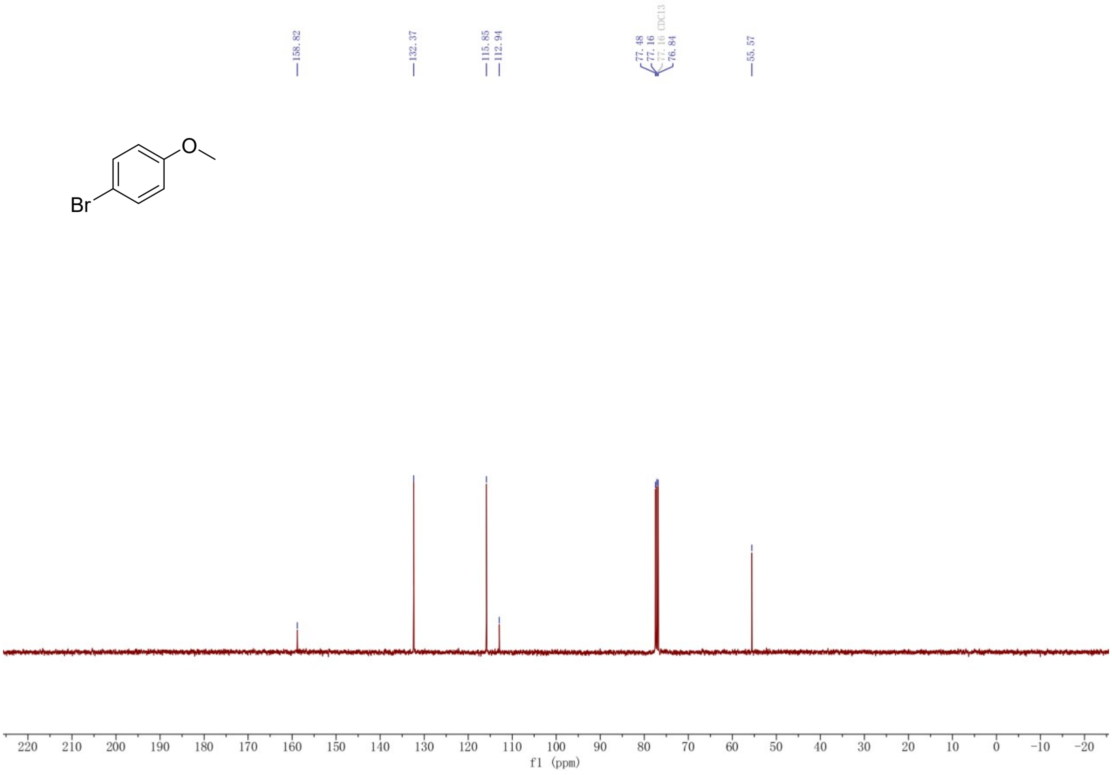

Figure S1. ¹H (top) and ¹³C (bottom) NMR spectra of 1-bromo-4-methoxybenzene(table 2-1).

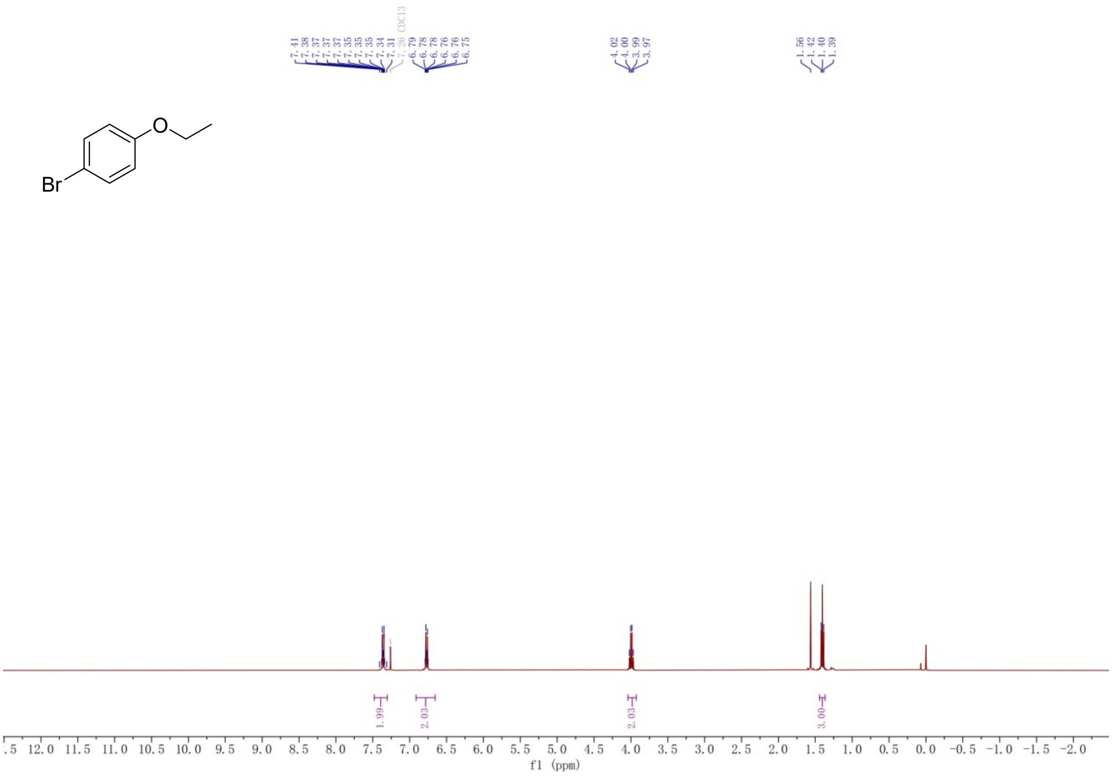

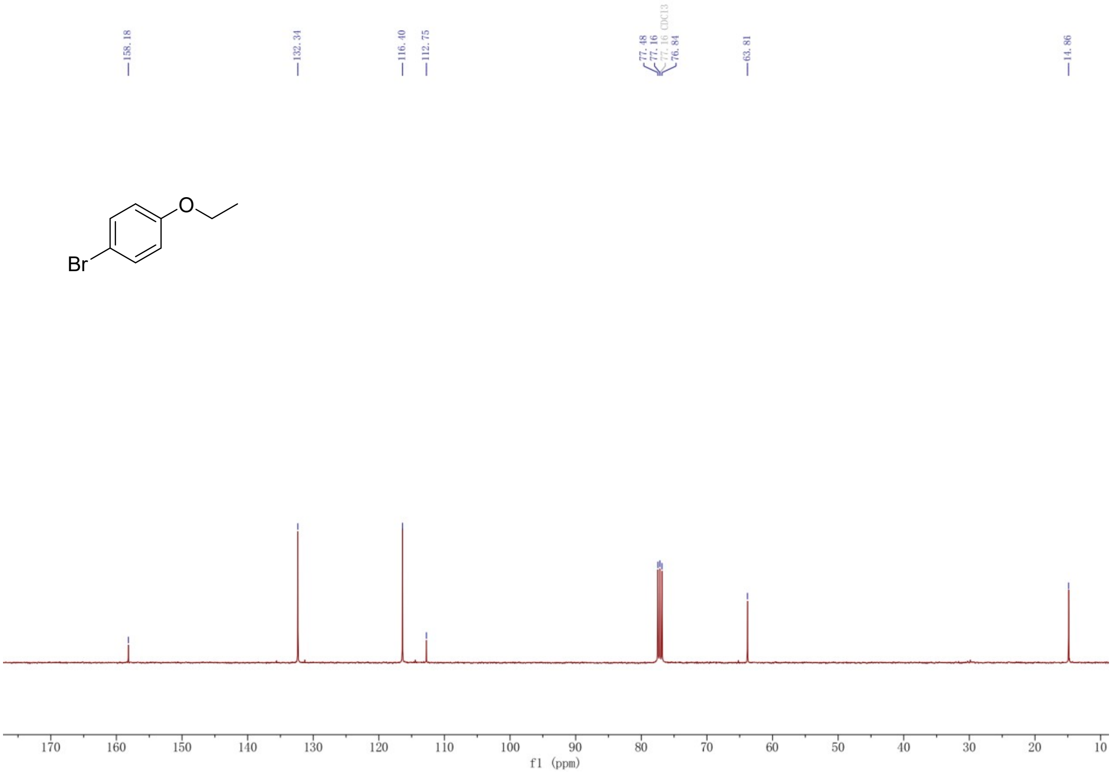

Figure S2. ^{1}H (top) and ^{13}C (bottom) NMR spectra of 1-bromo-4-ethoxybenzene(table 2-2).

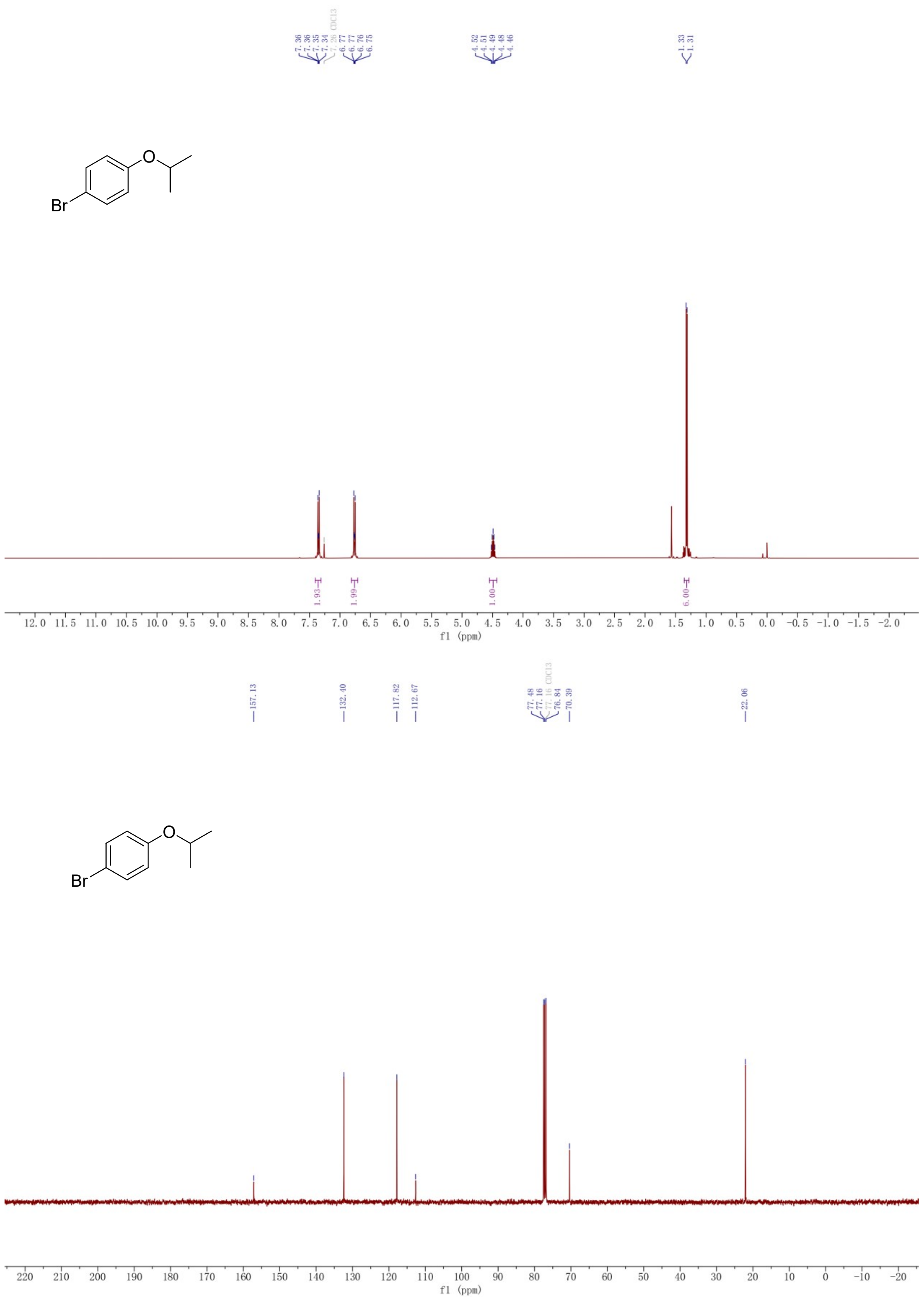

Figure S3. ^{1}H **(top) and** ^{13}C **(bottom) NMR spectra of 1-bromo-4-isopropoxybenzene(table 2-3).**

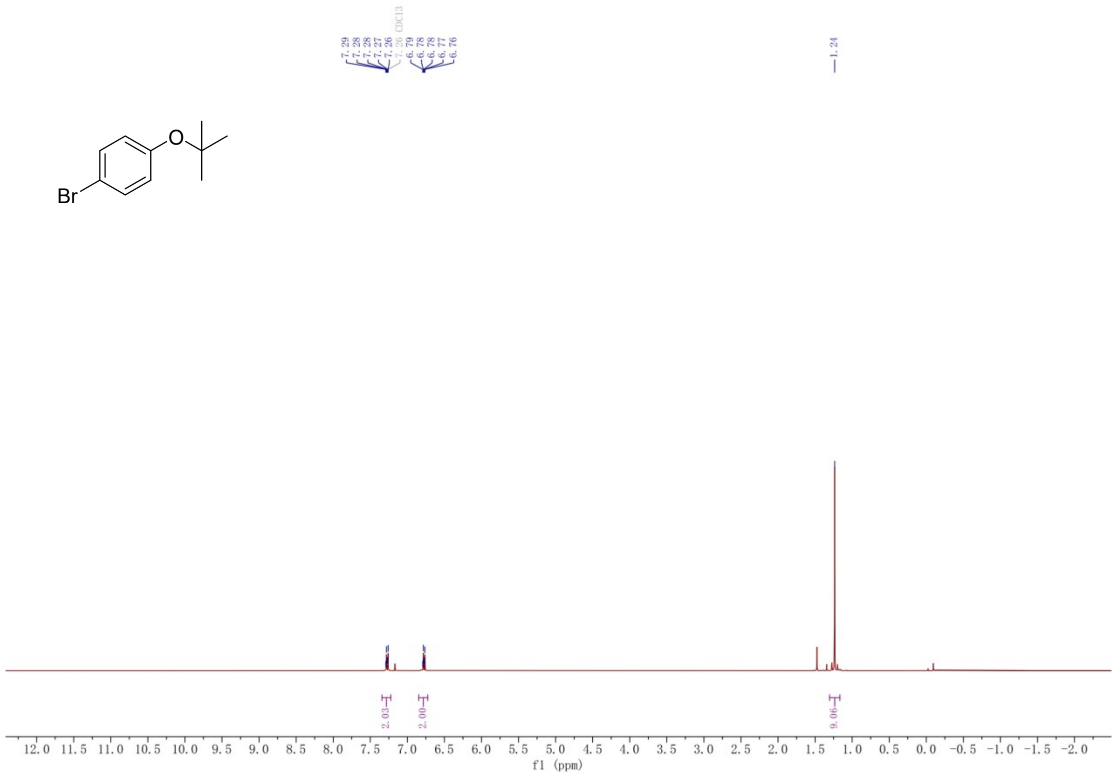

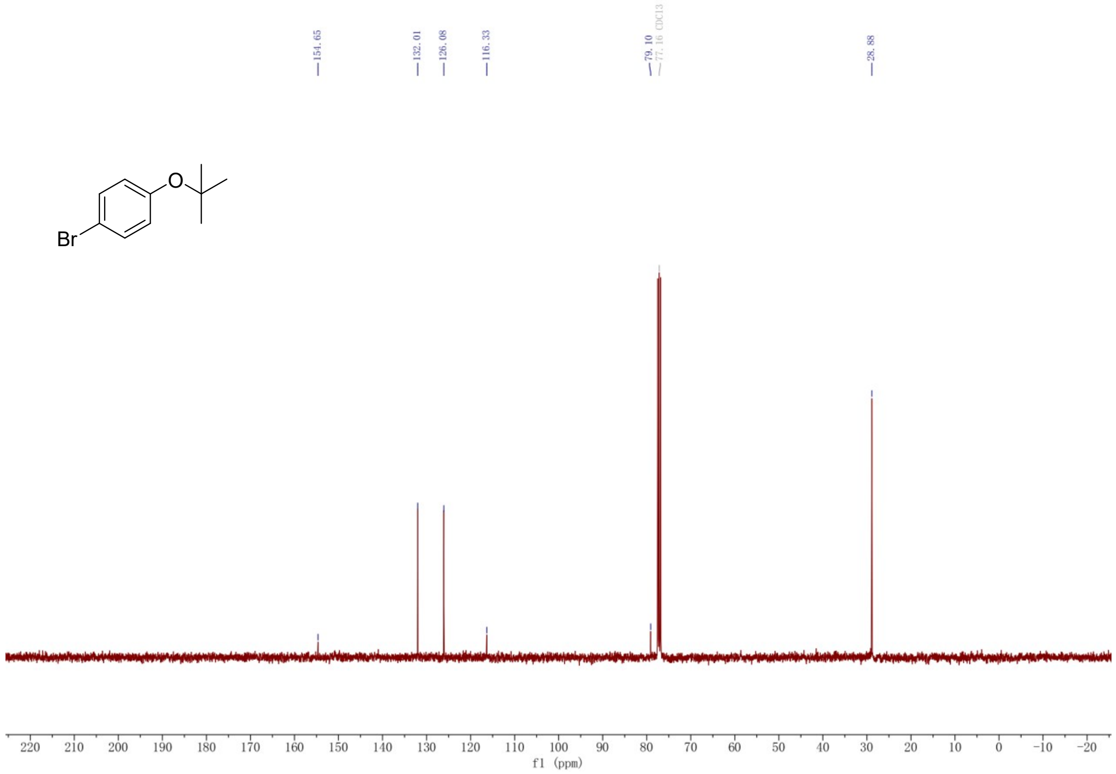

Figure S4. ^{1}H **(top) and** ^{13}C **(bottom) NMR spectra of 1-bromo-4-(tert-butoxy)benzene(table 2-4).**

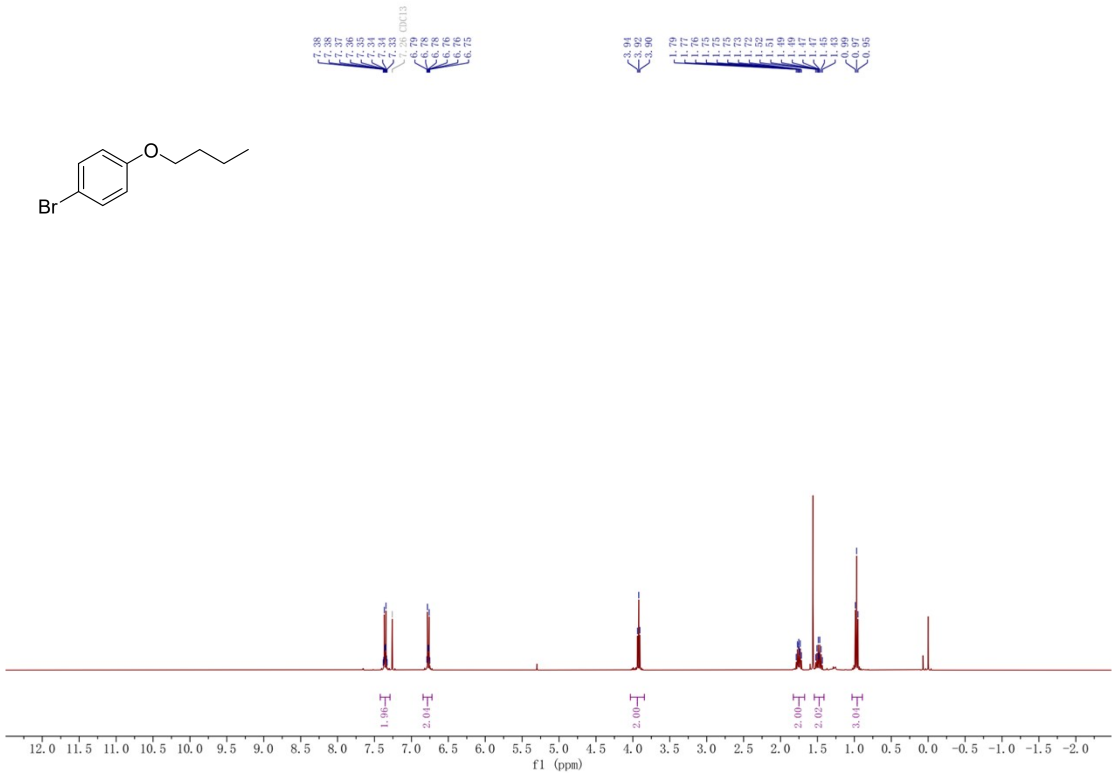

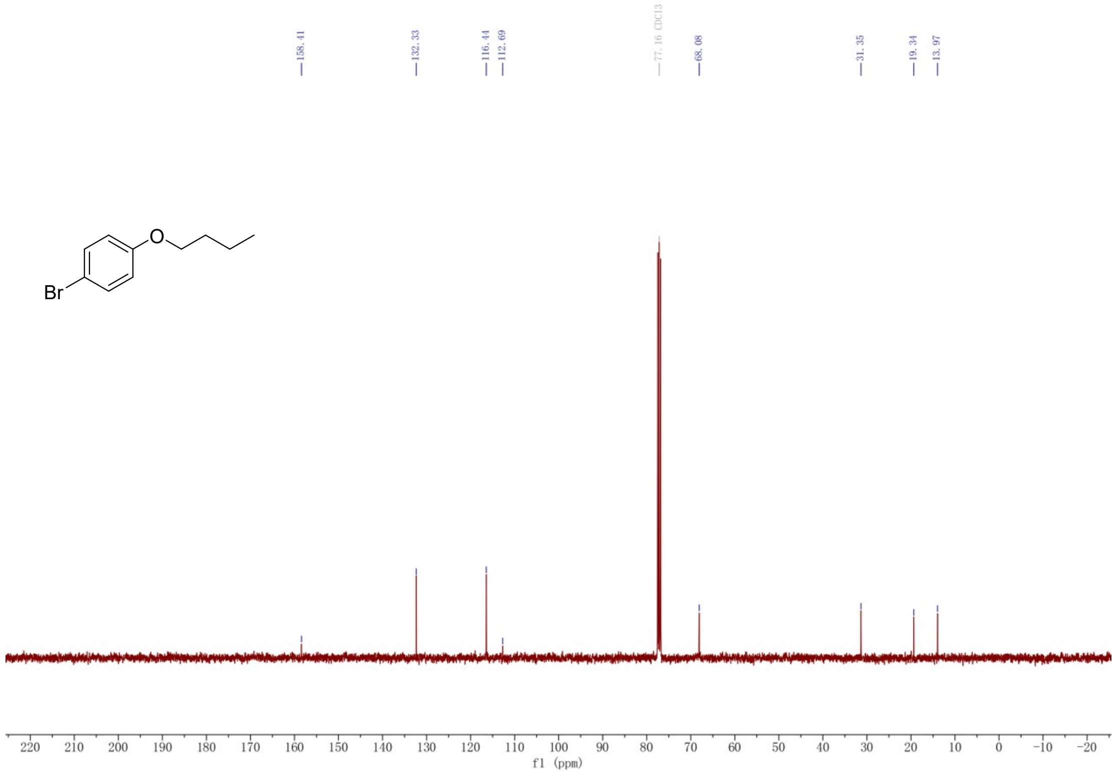

Figure S5. ^{1}H (top) and ^{13}C (bottom) NMR spectra of 1-bromo-4-butoxybenzene(table 2-5).

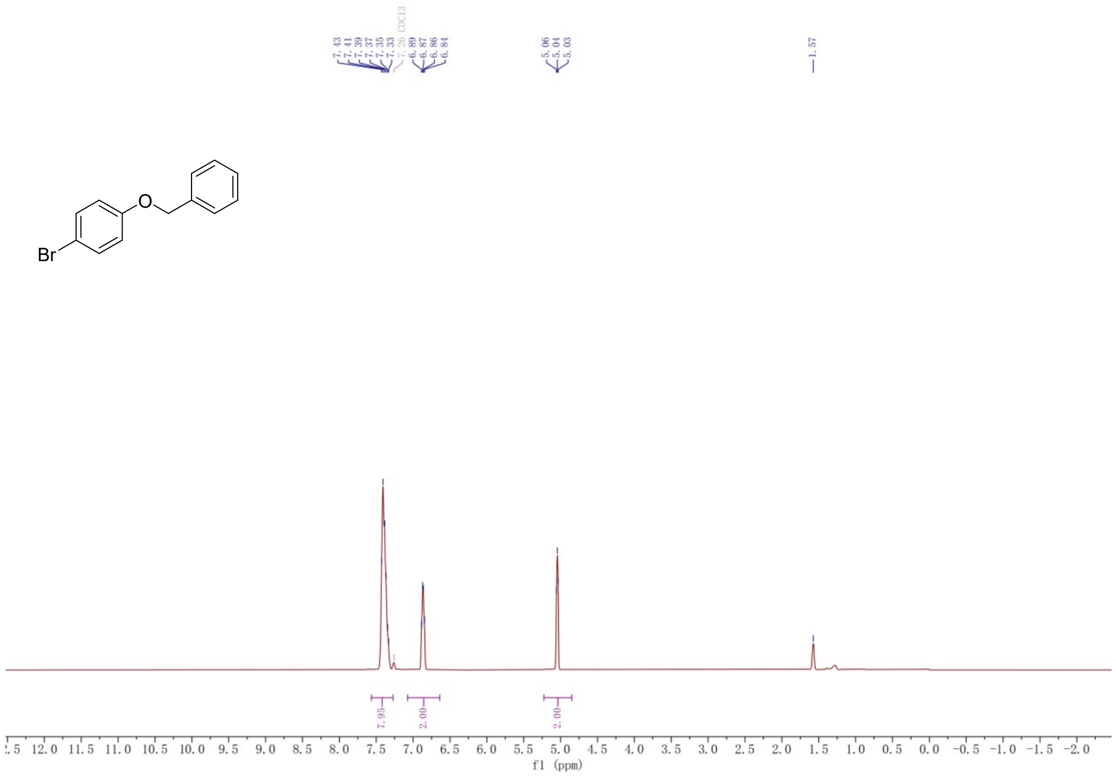

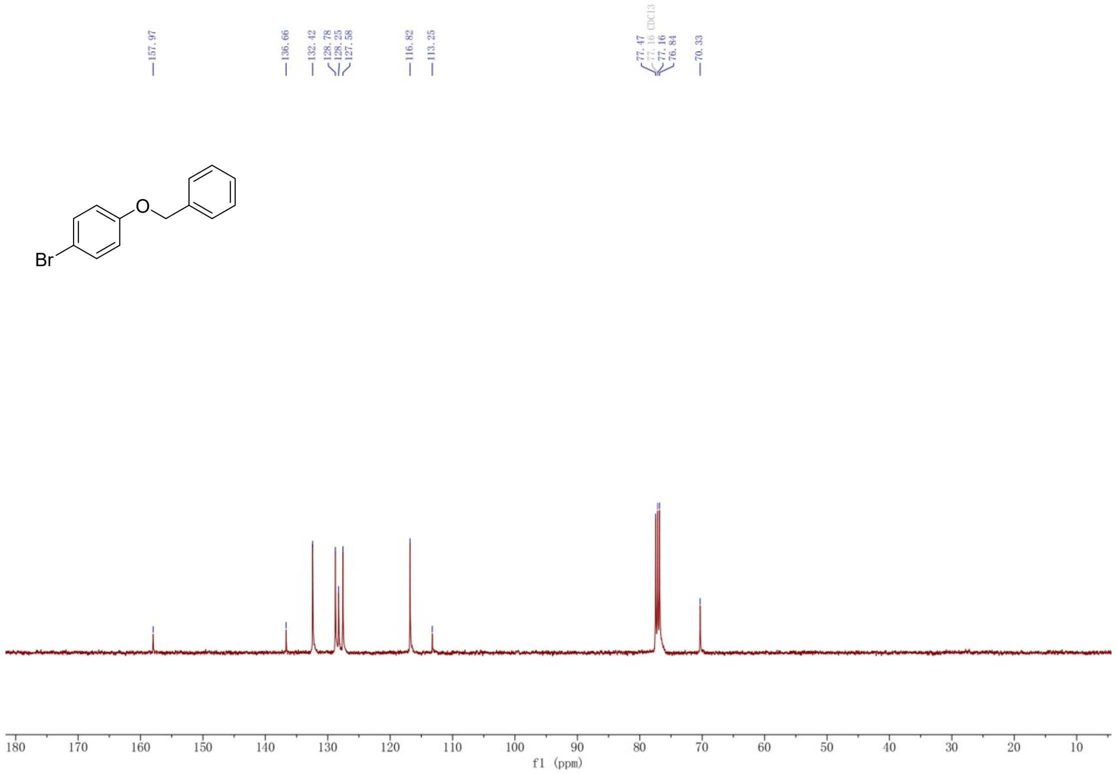

Figure S6. **^{1}H (top) and ^{13}C (bottom) NMR spectra of 1-(benzyloxy)-4-bromobenzene(table 2-6).**

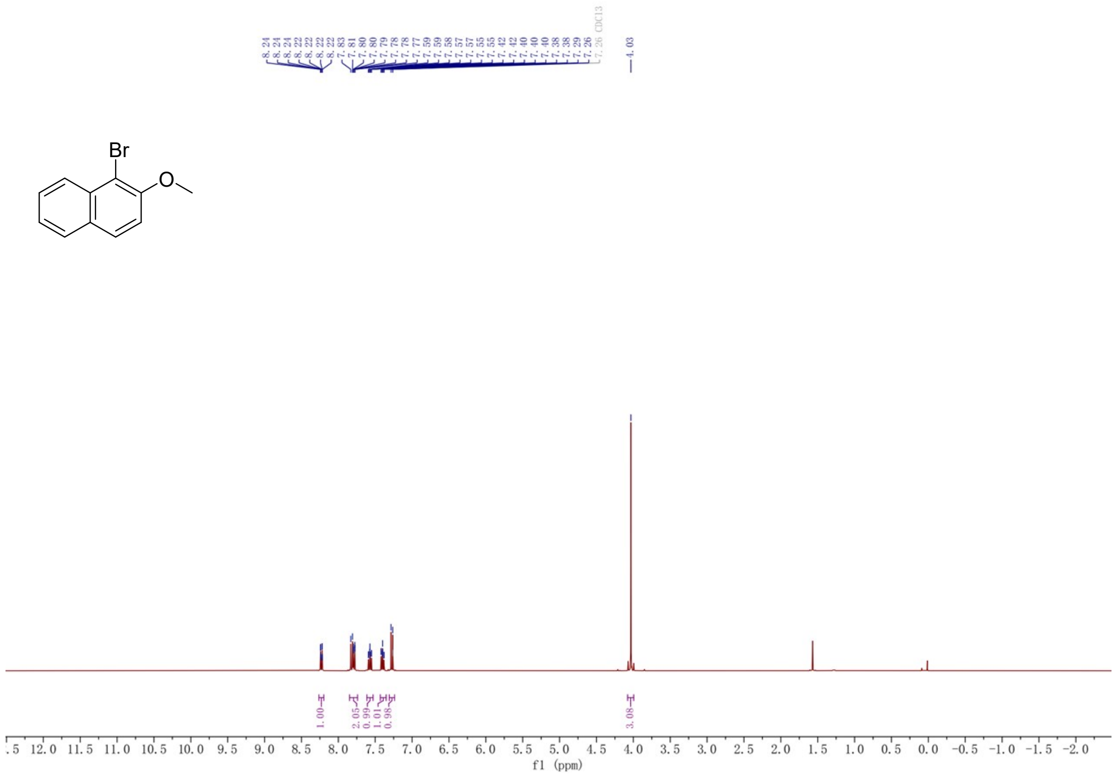

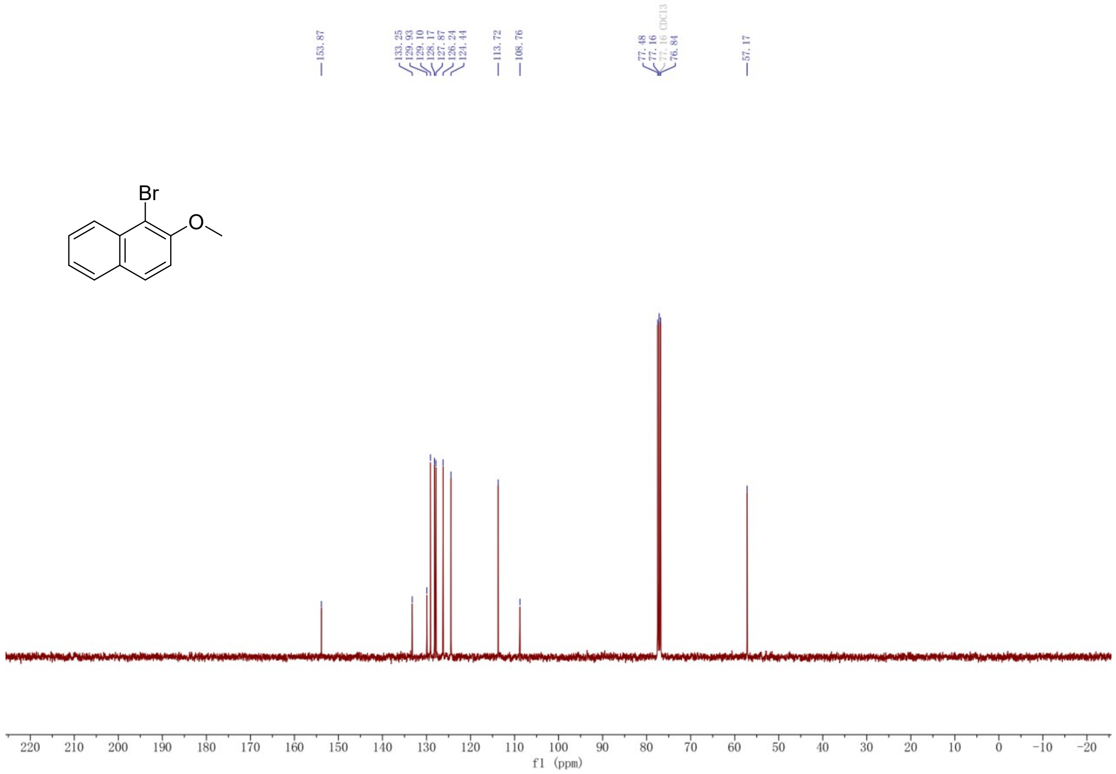

Figure S7. ^{1}H (top) and ^{13}C (bottom) NMR spectra of 1-bromo-2-methoxynaphthalene(table 2-7).

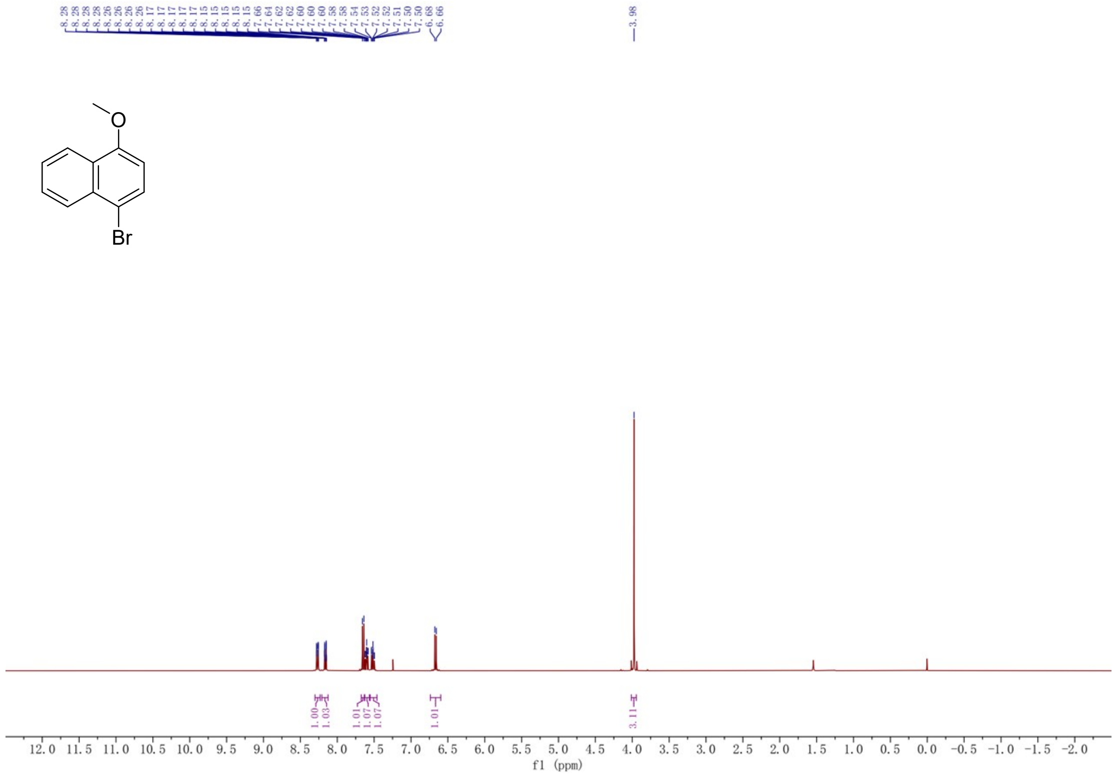

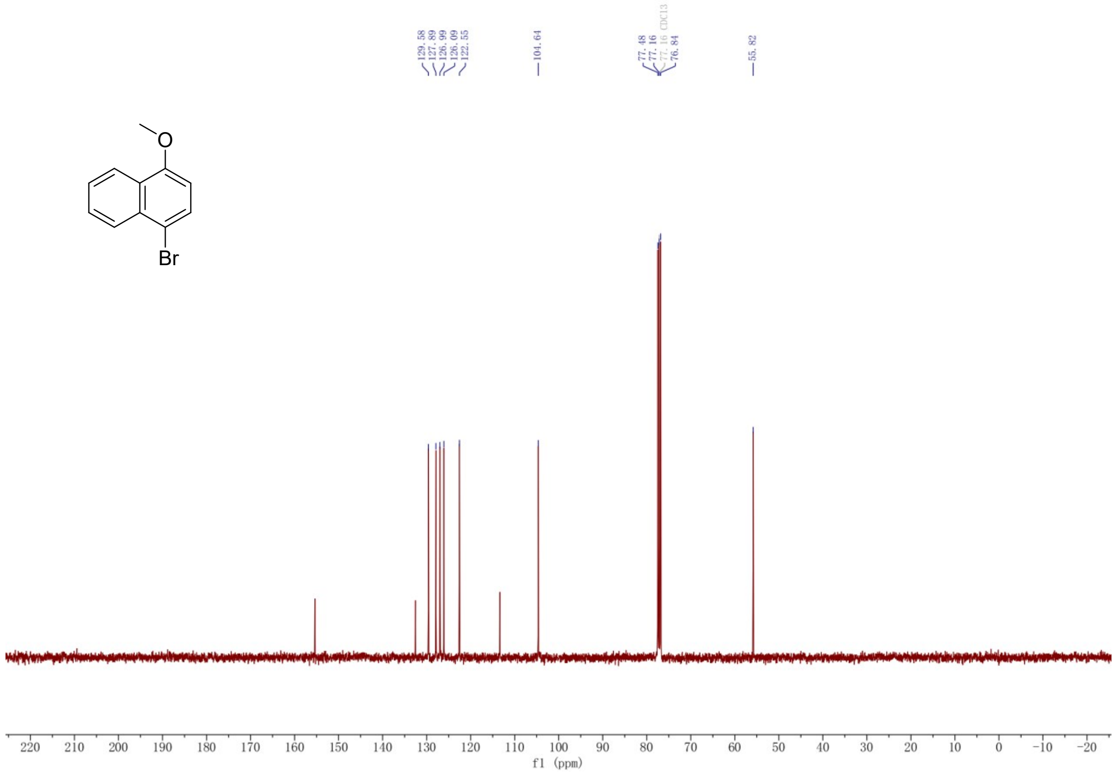

Figure S8. ^{1}H (top) and ^{13}C (bottom) NMR spectra of 1-bromo-4-methoxynaphthalene(table 2-8).

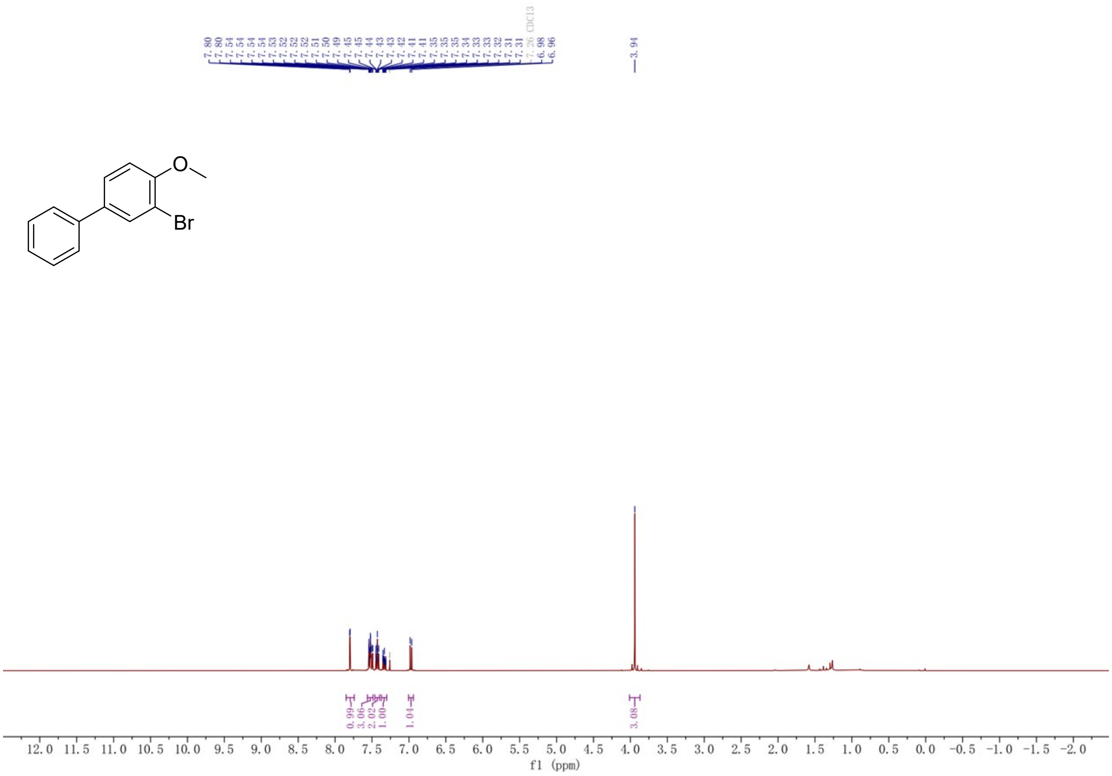

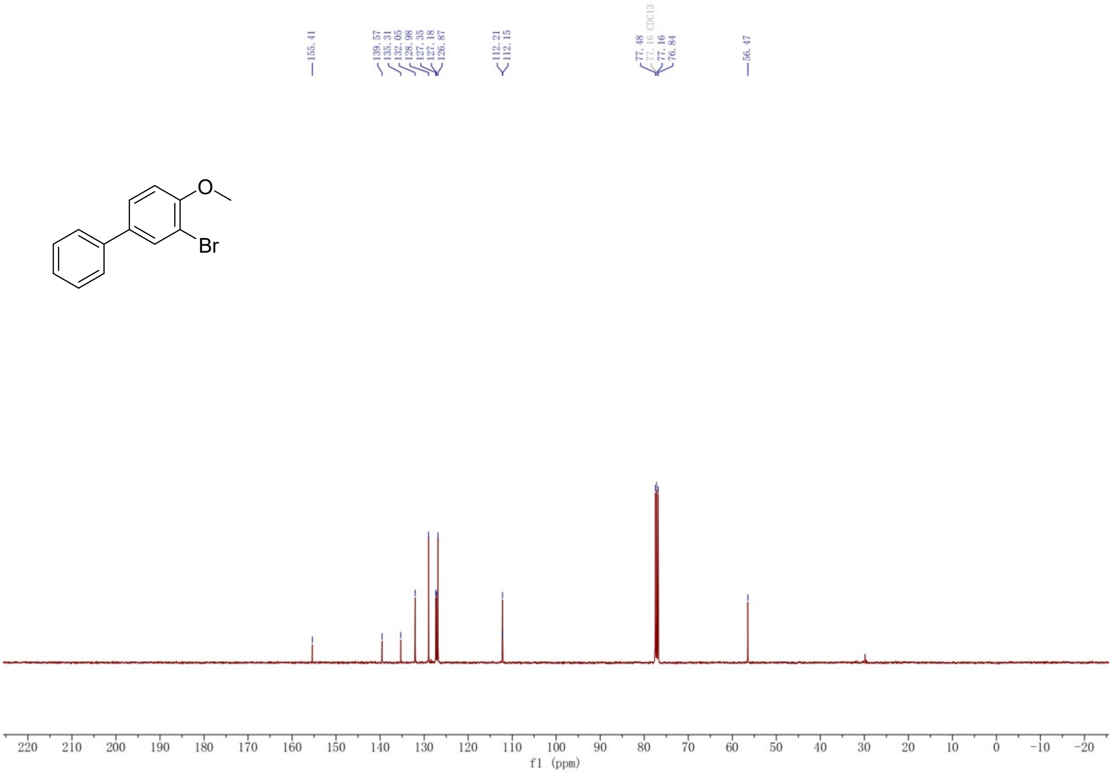

Figure S9. ^{1}H (top) and ^{13}C (bottom) NMR spectra of 3-bromo-4-methoxy-1,1'-biphenyl(table 2-9).

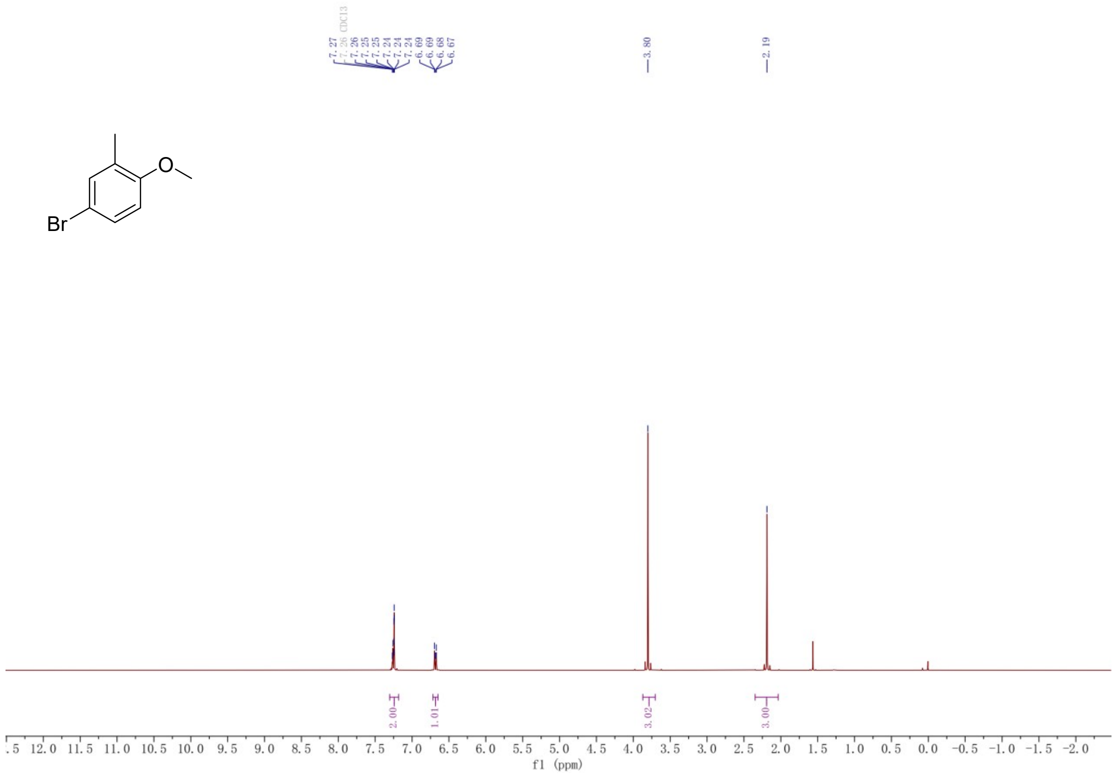

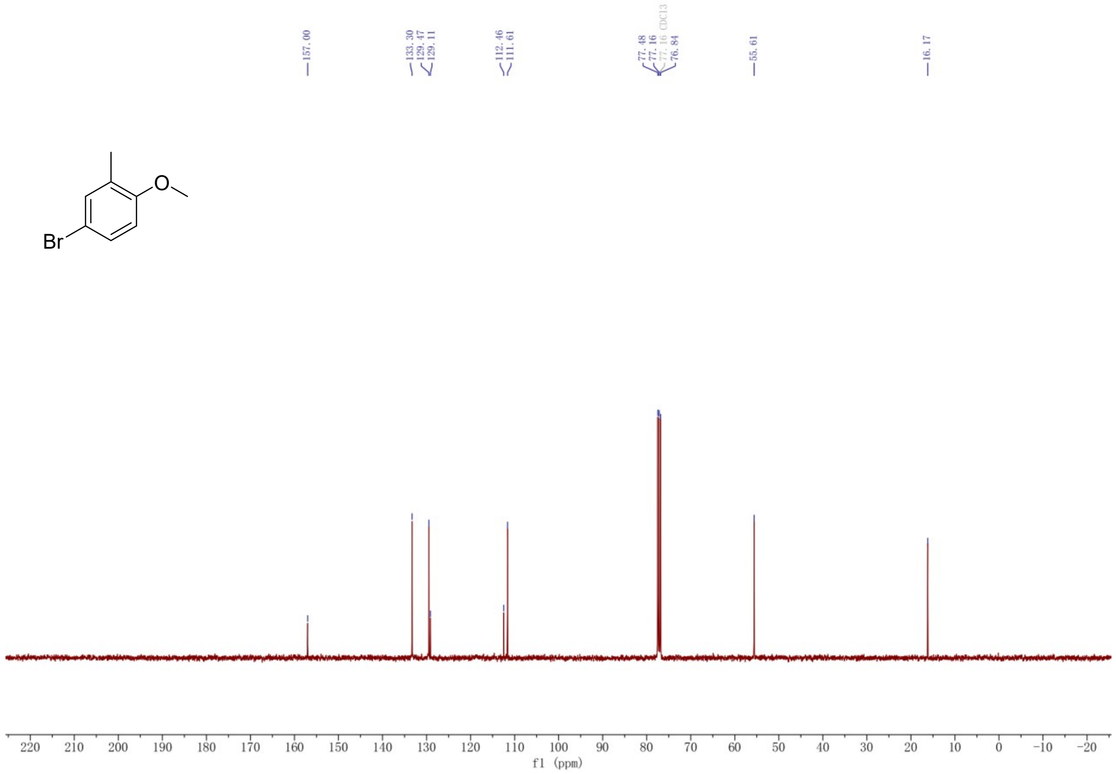

Figure S10. ^{1}H **(top) and** ^{13}C **(bottom) NMR spectra of 4-bromo-1-methoxy-2-methylbenzene(table 2-10).**

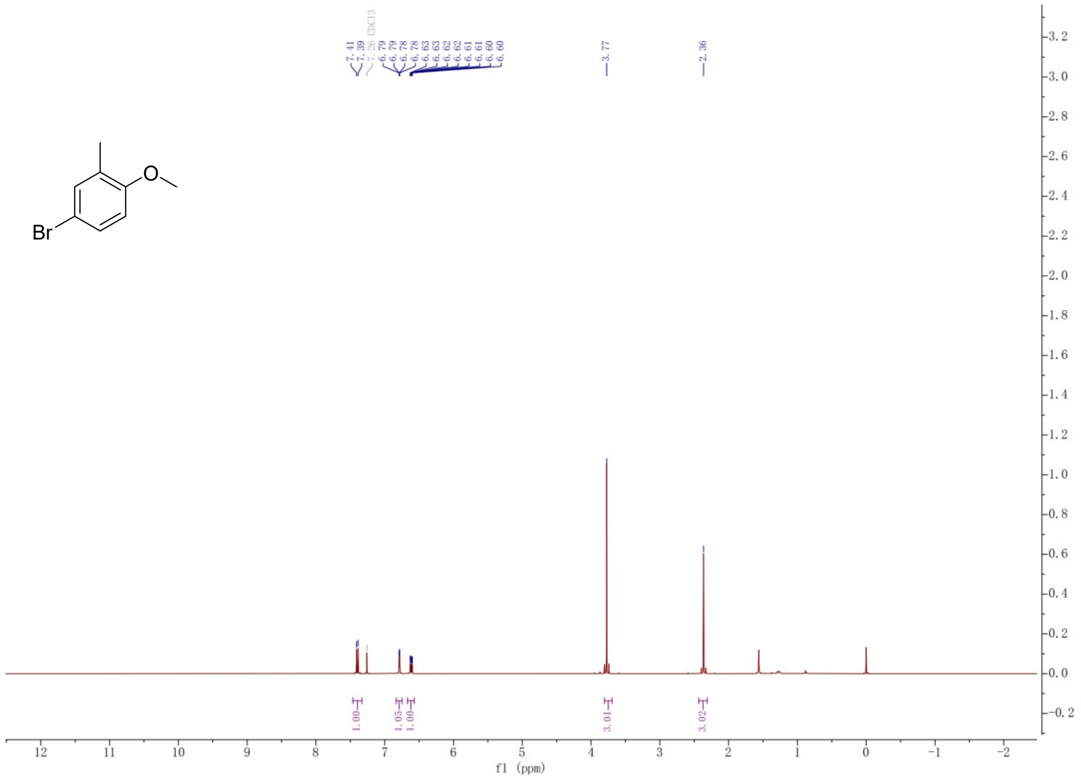

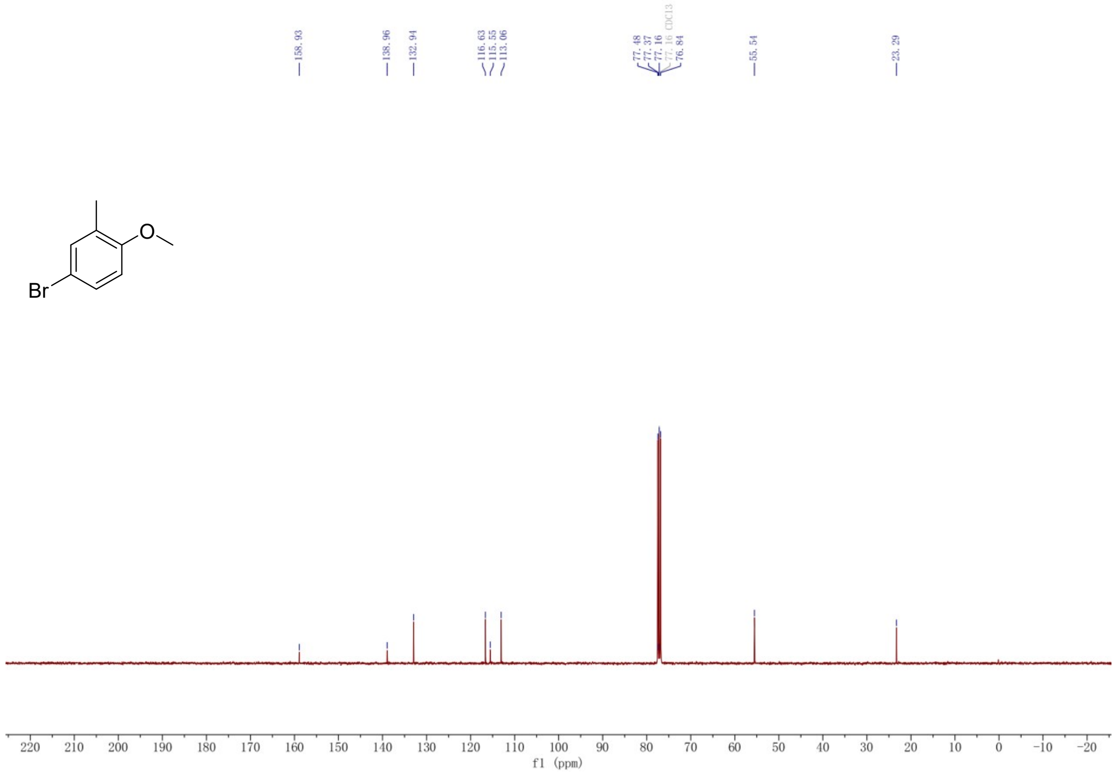

Figure S11. **^{1}H (top) and ^{13}C (bottom) NMR spectra of 1-bromo-4-methoxy-2-methylbenzene(table 2-11).**

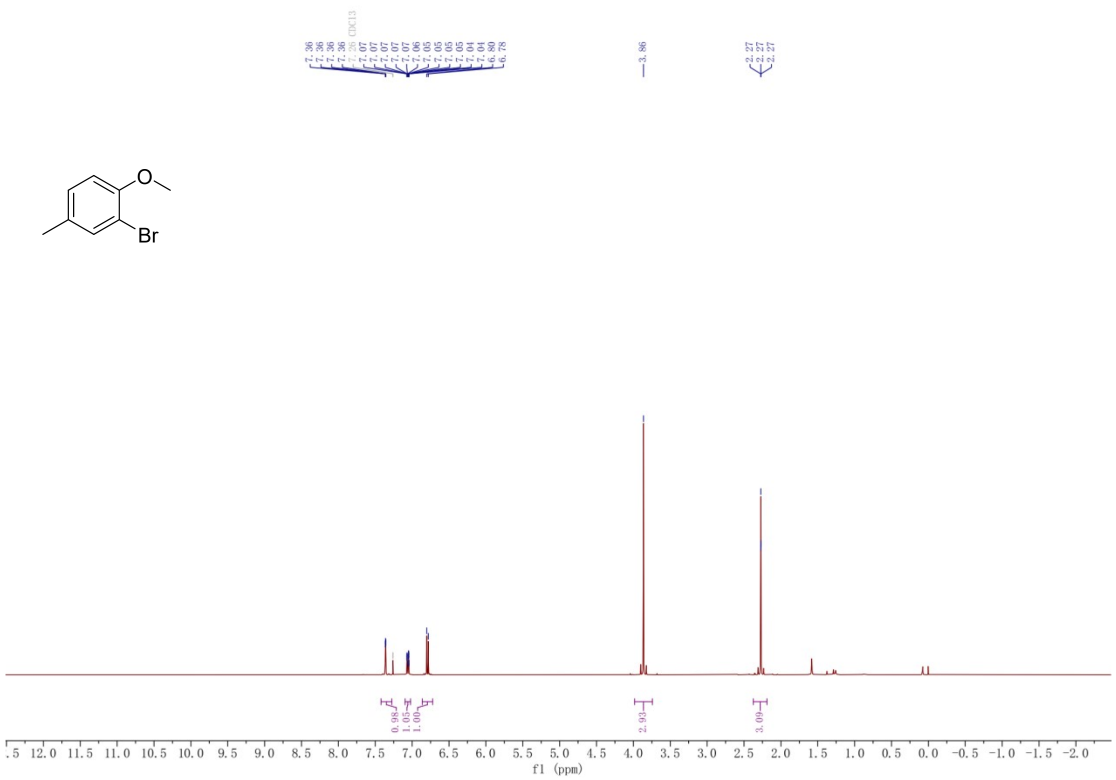

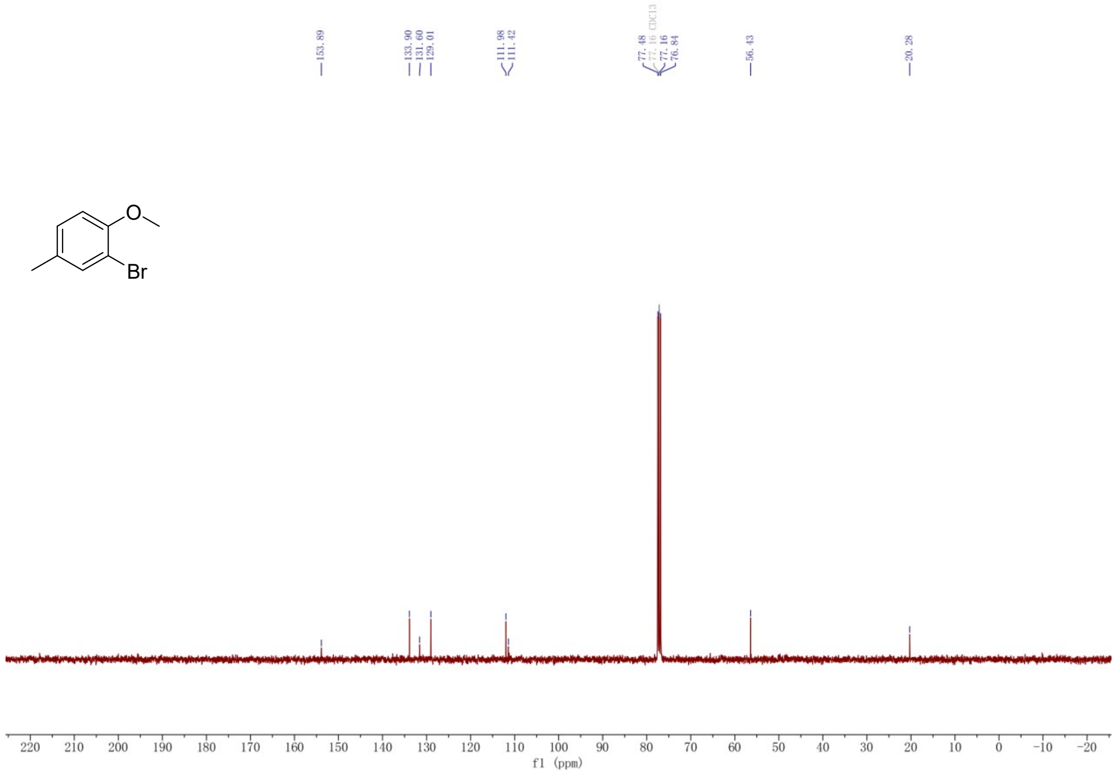

Figure S12. **^{1}H (top) and ^{13}C (bottom) NMR spectra of 2-bromo-1-methoxy-4-methylbenzene(table 2-12).**

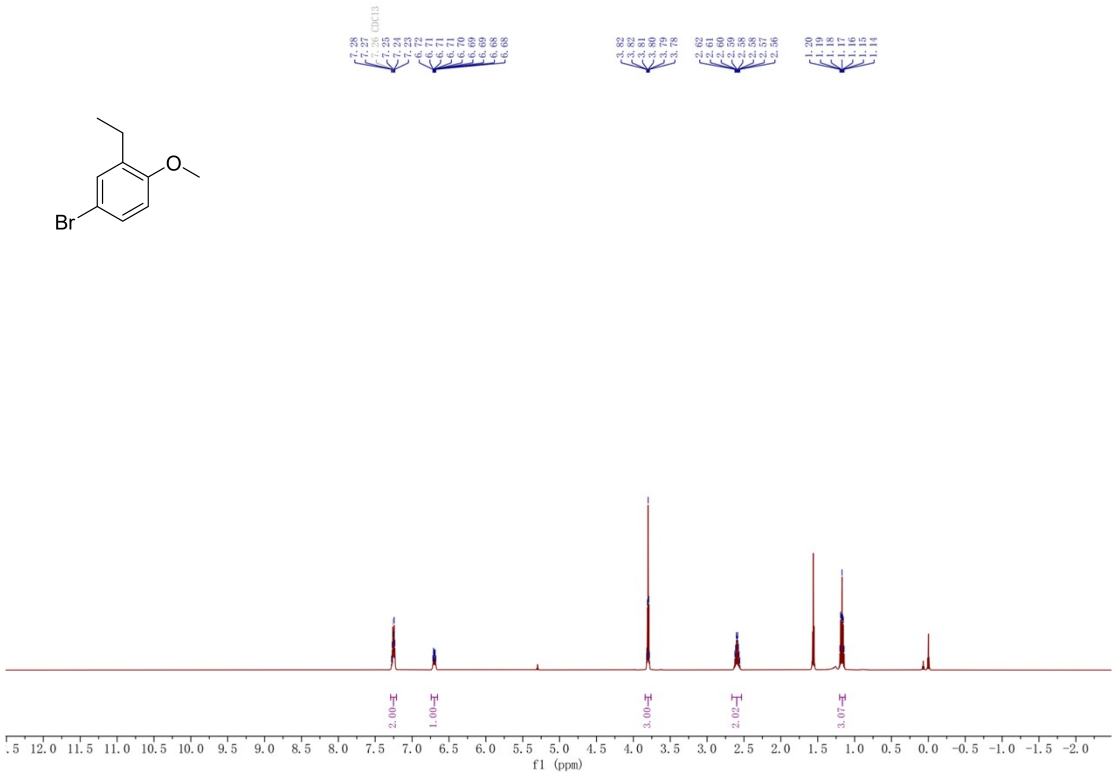

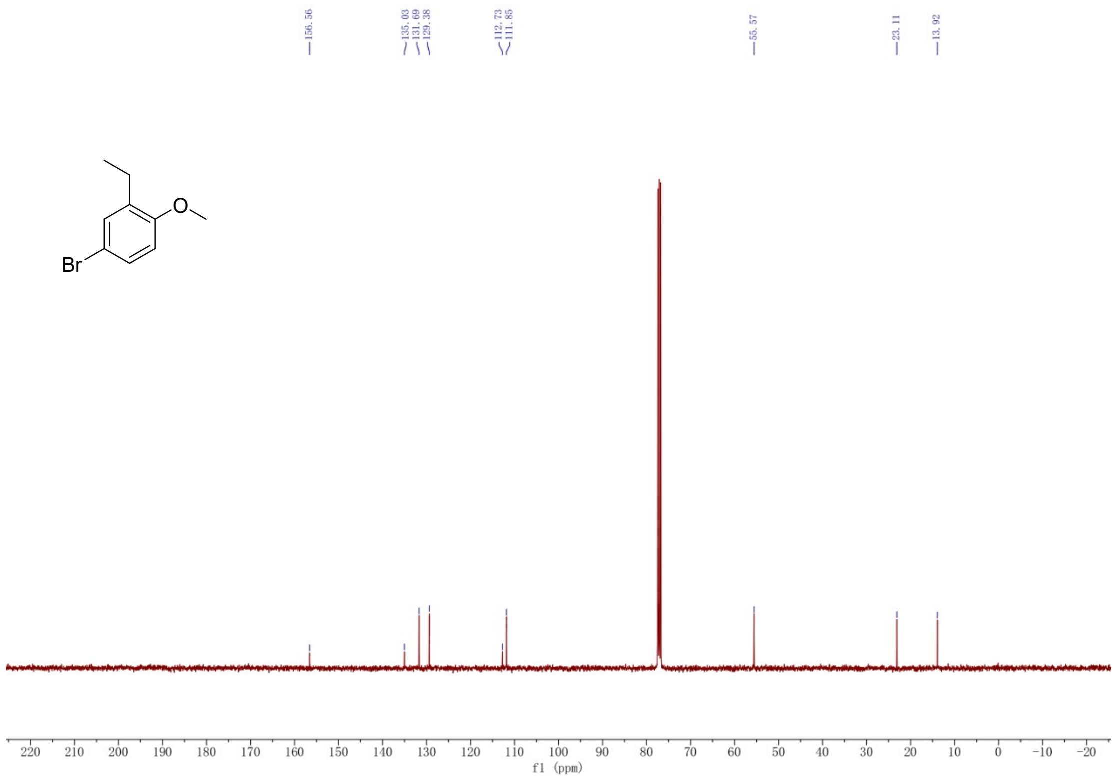

Figure S13. ^{1}H (top) and ^{13}C (bottom) NMR spectra of 4-bromo-2-ethyl-1-methoxybenzene(table 2-13).

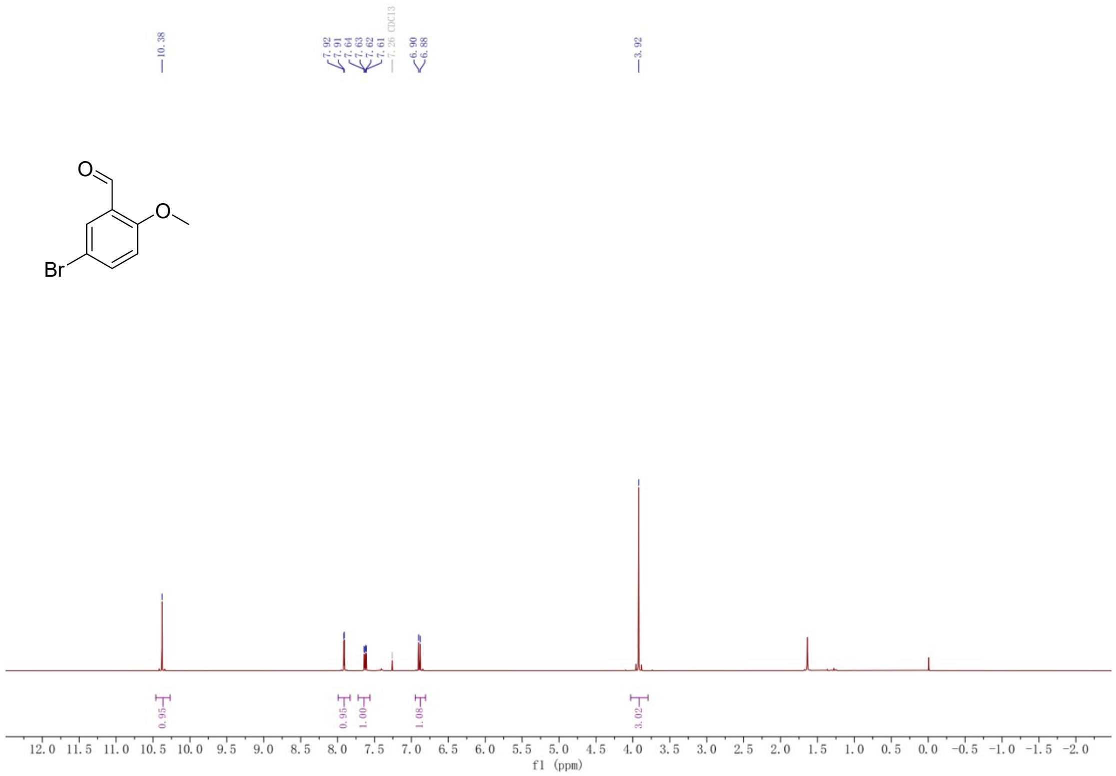

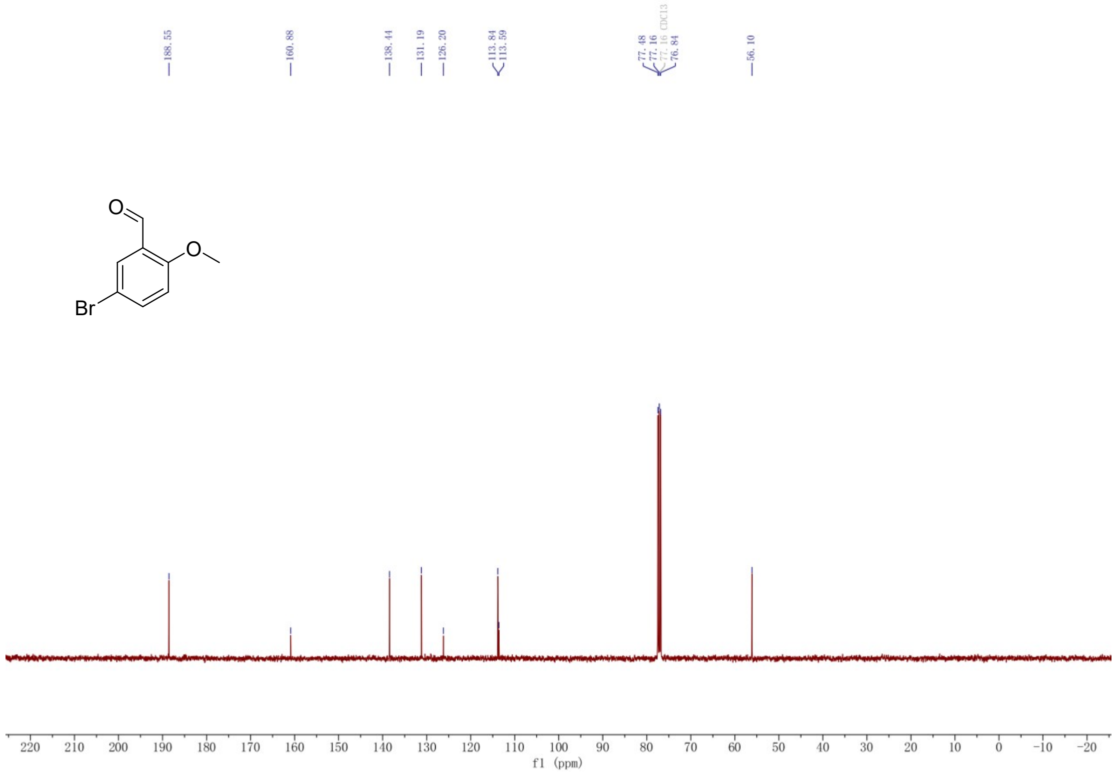

Figure S14. ^{1}H (top) and ^{13}C (bottom) NMR spectra of 5-bromo-2-methoxybenzaldehyde(table 2-14).

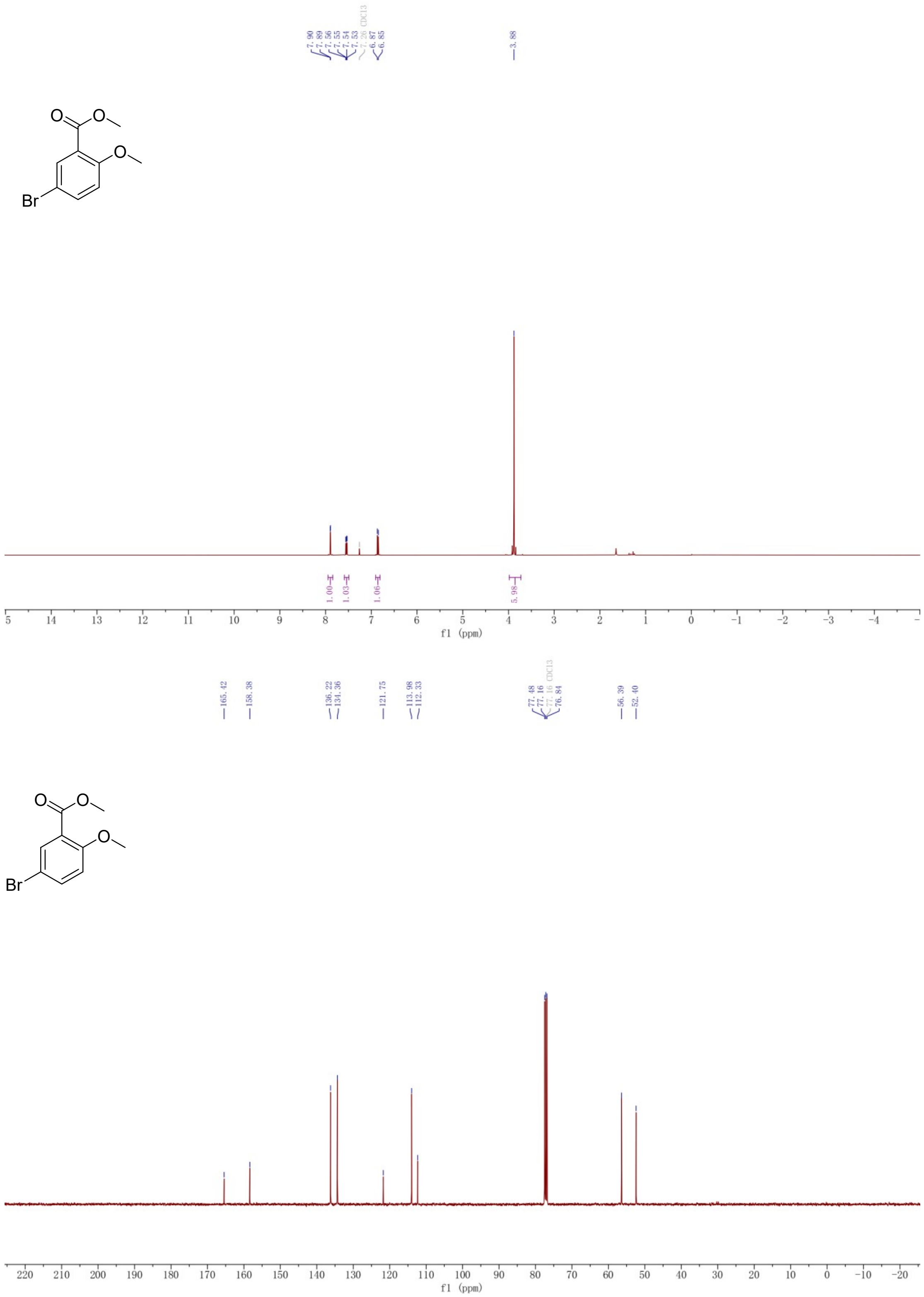

Figure S15. ^{1}H (top) and ^{13}C (bottom) NMR spectra of methyl 5-bromo-2-methoxybenzoate(table 2-15).

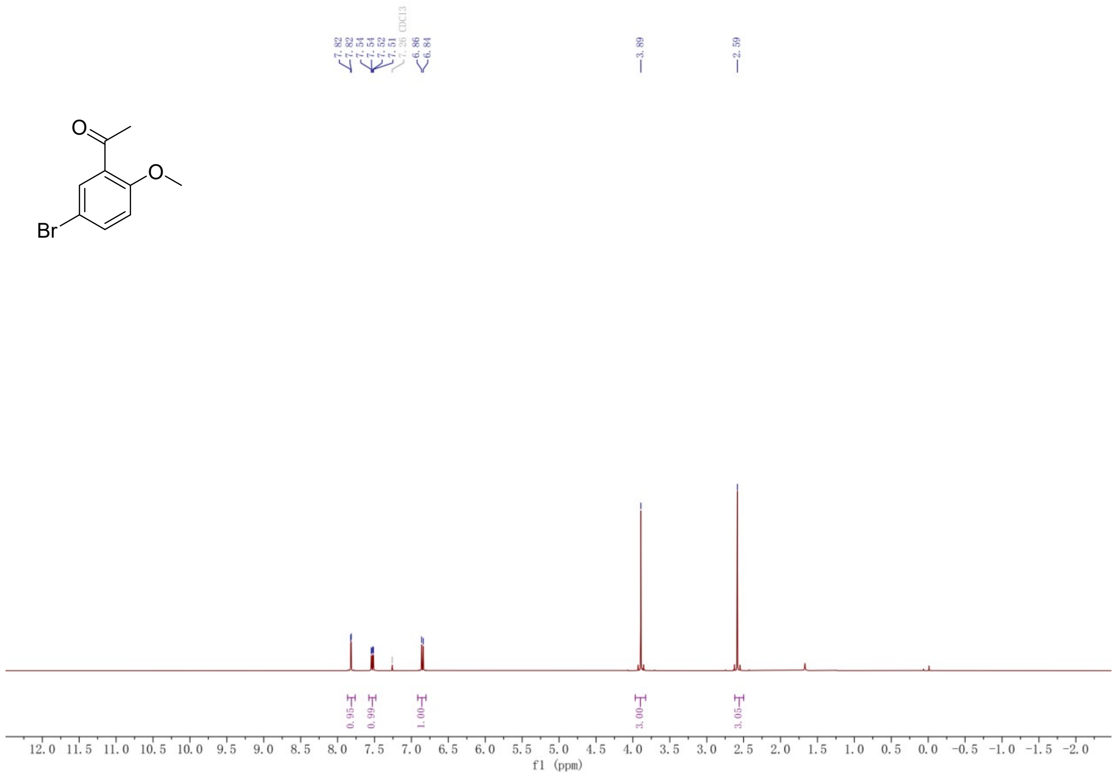

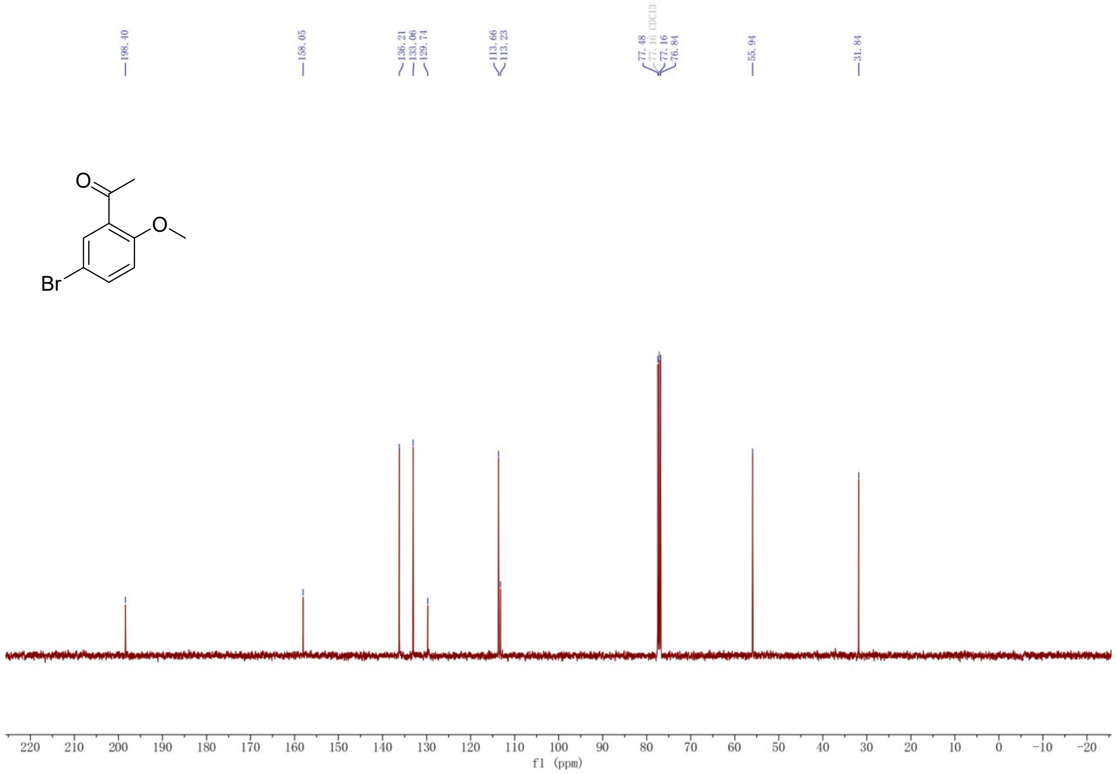

Figure S16. **^{1}H (top) and ^{13}C (bottom) NMR spectra of 1-(5-bromo-2-methoxyphenyl)ethanone(table 2-16-1).**

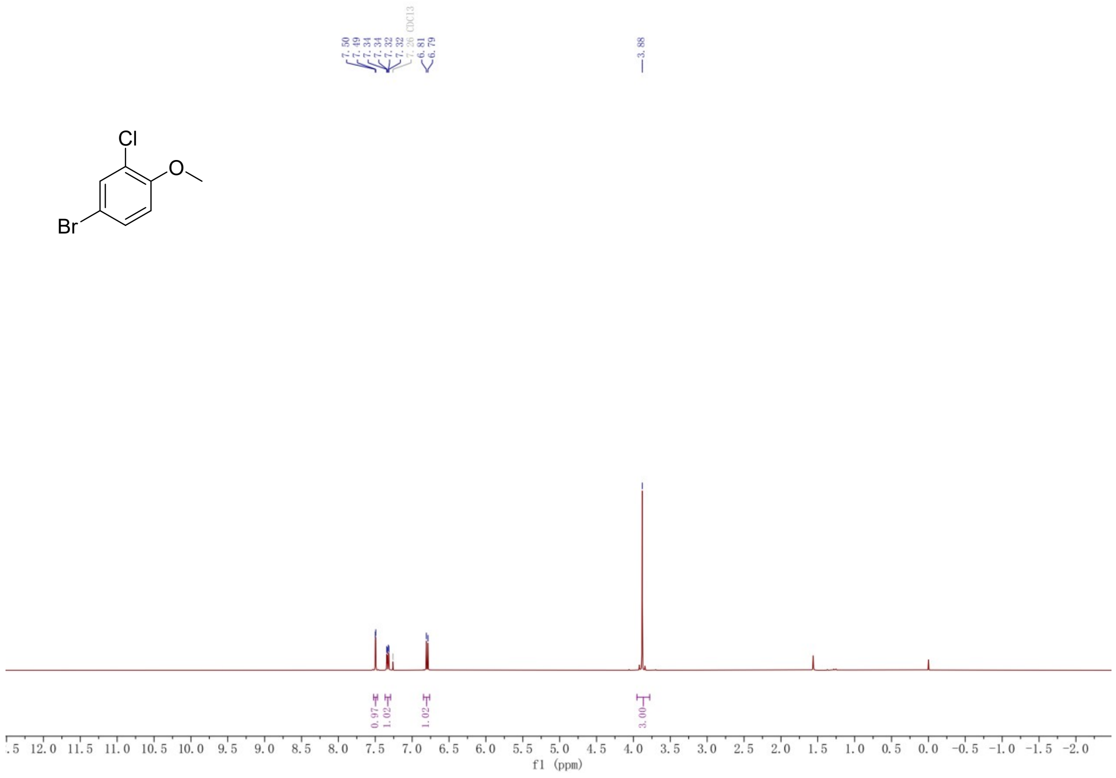

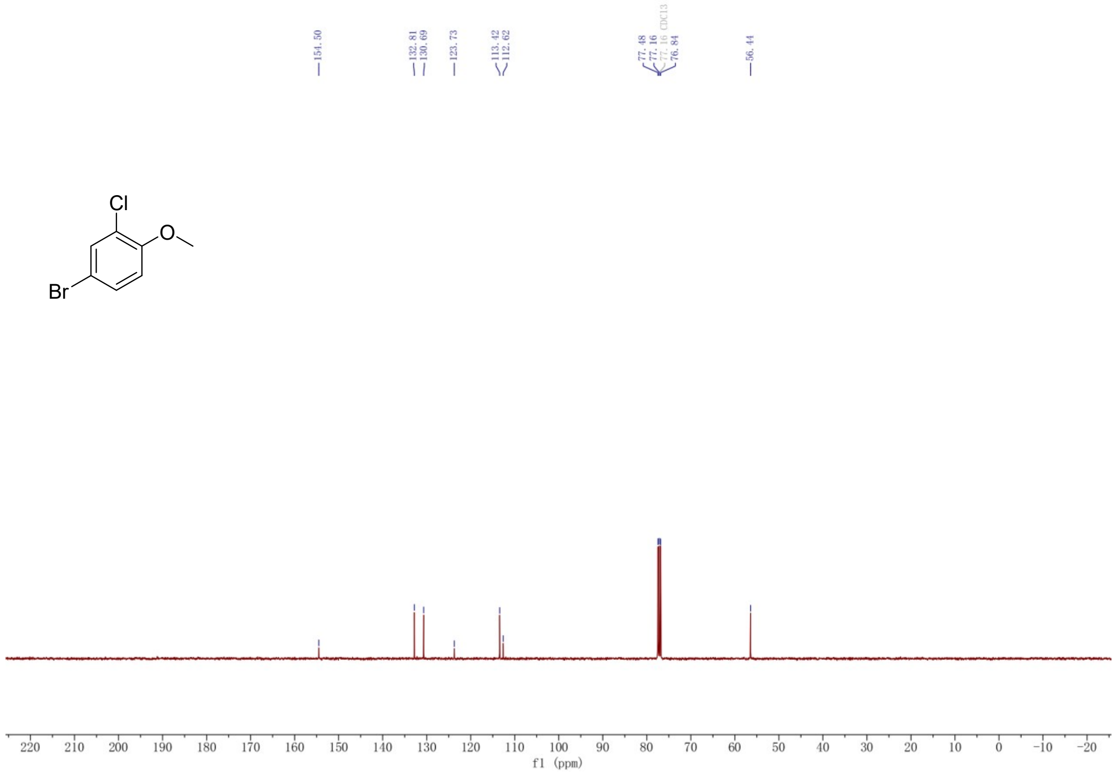

Figure S17. **^{1}H (top) and ^{13}C (bottom) NMR spectra of 4-bromo-2-chloro-1-methoxybenzene(table 2-17).**

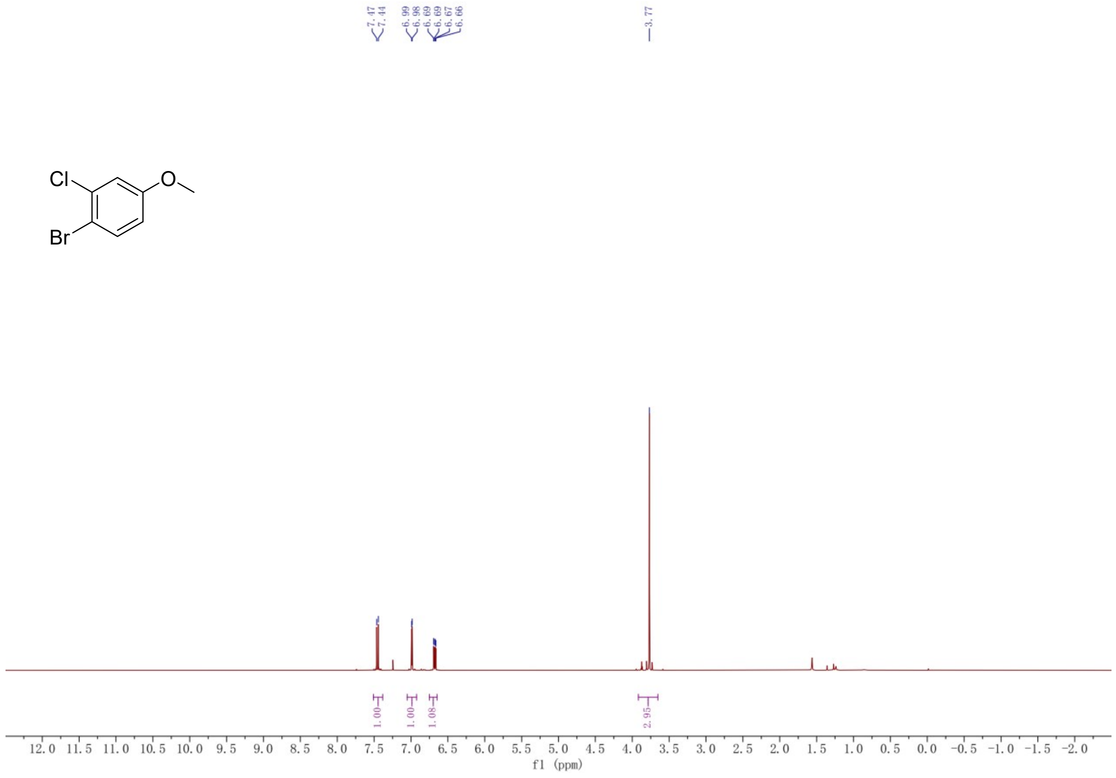

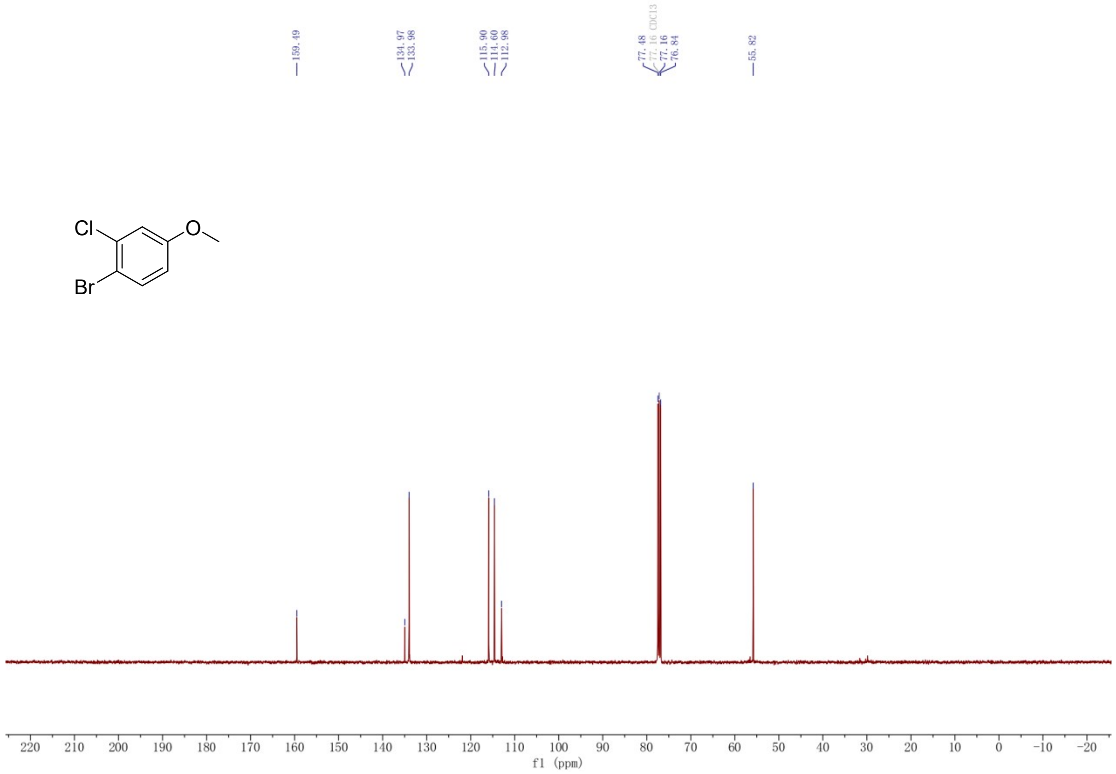

Figure S18. ^{1}H (top) and ^{13}C (bottom) NMR spectra of 1-bromo-2-chloro-4-methoxybenzene(table 2-18).

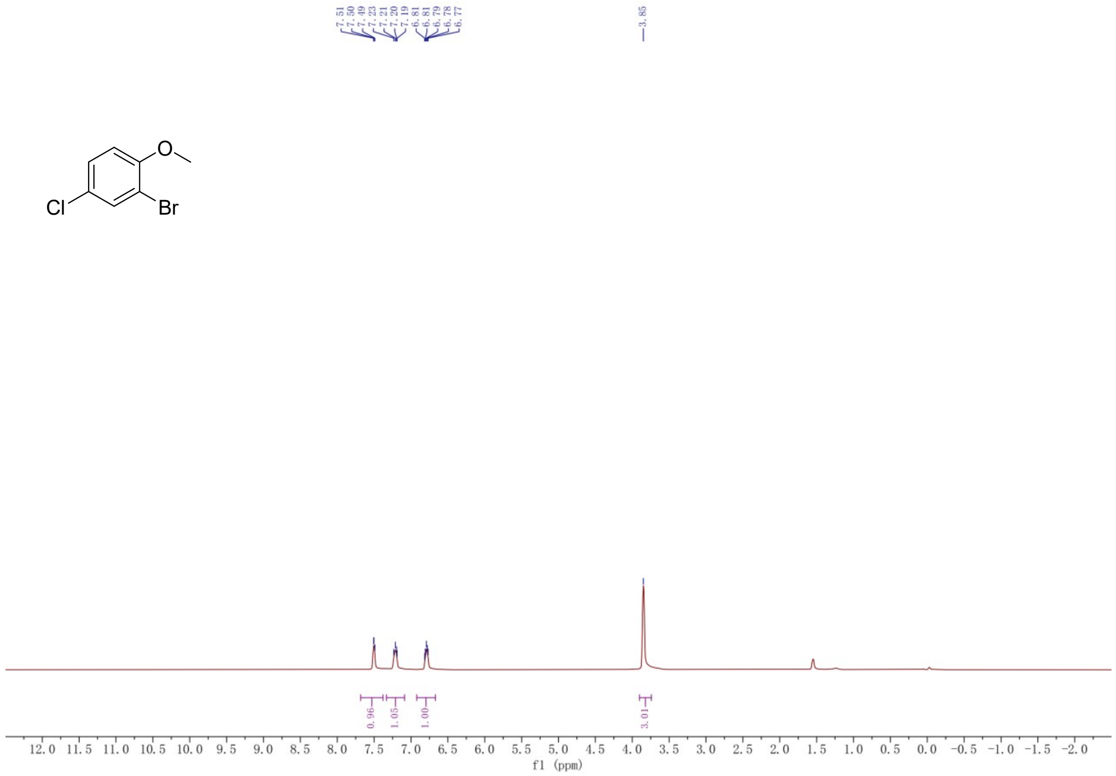

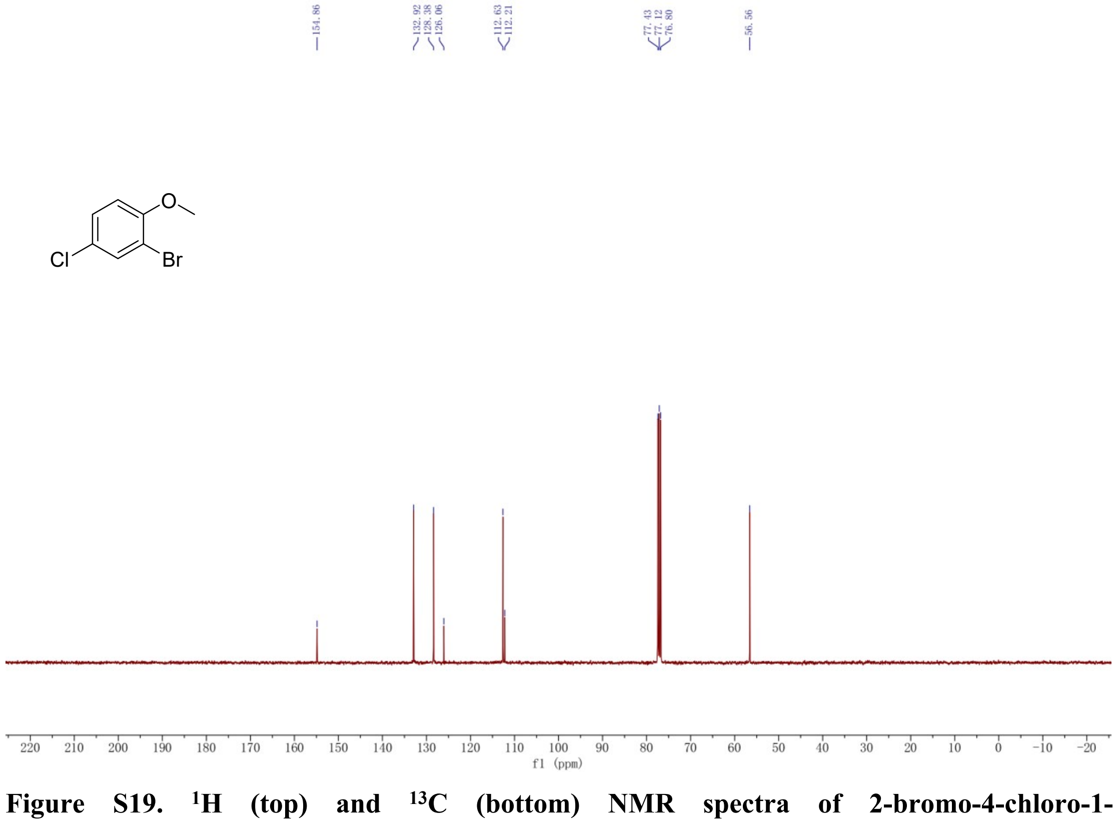

Figure S19. ^{1}H **(top) and** ^{13}C **(bottom) NMR spectra of 2-bromo-4-chloro-1-methoxybenzene(table 2-19).**

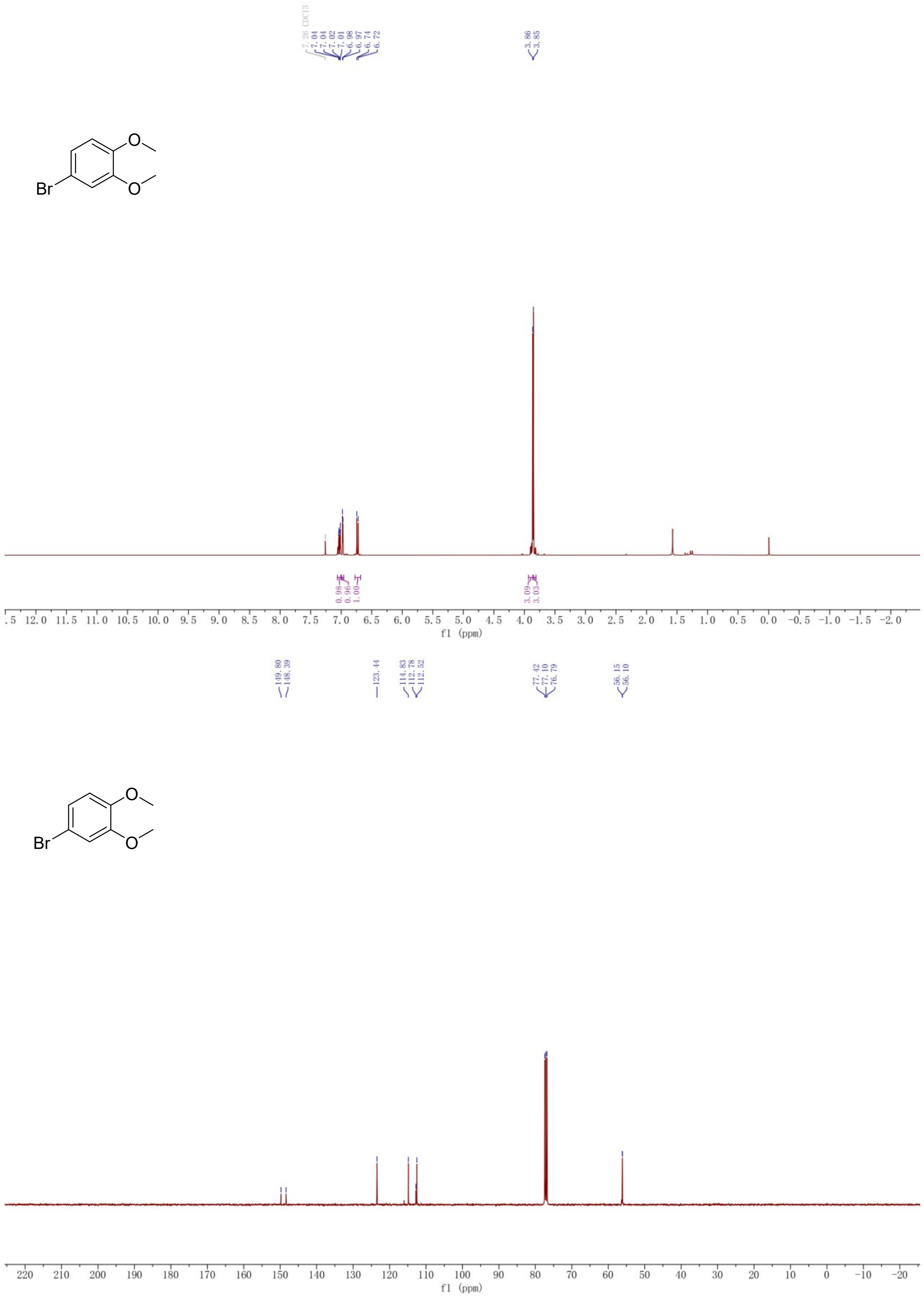

Figure S20. ^1H (top) and ^{13}C (bottom) NMR spectra of 4-bromo-1,2-dimethoxybenzene(table 2-22-1).

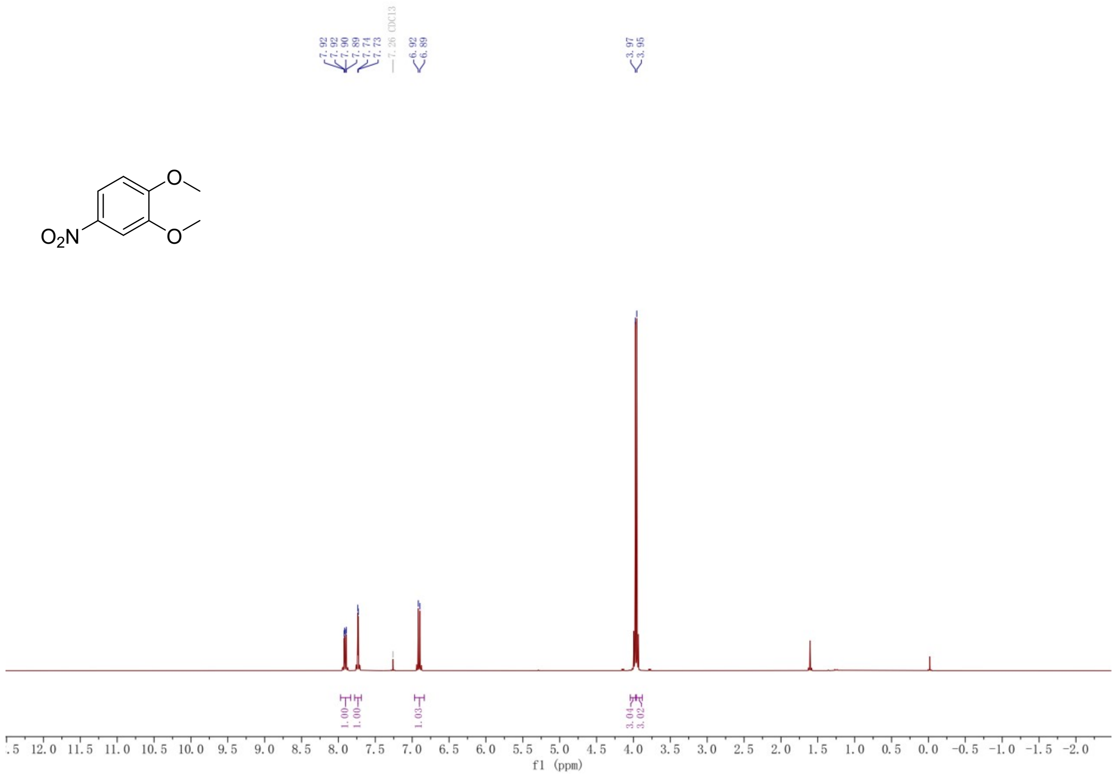

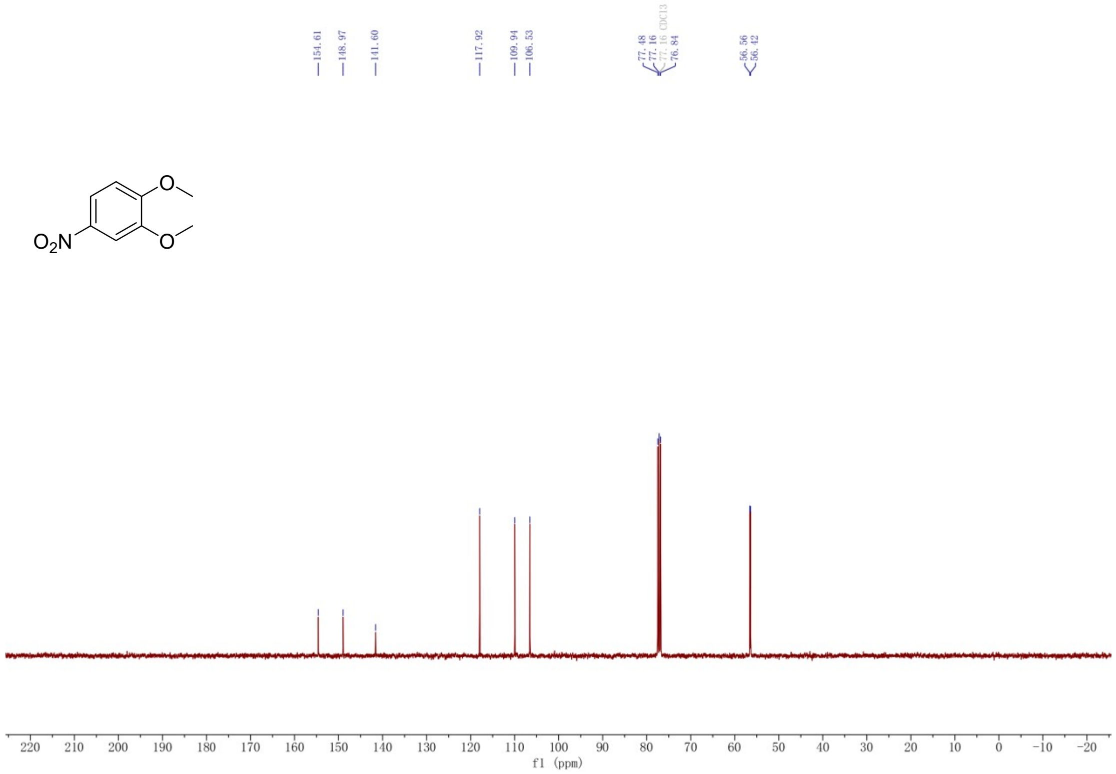

Figure S21. **^{1}H (top) and ^{13}C (bottom) NMR spectra of 1,2-dimethoxy-4-nitrobenzene(table 2-22-2 and table 3-18-2).**

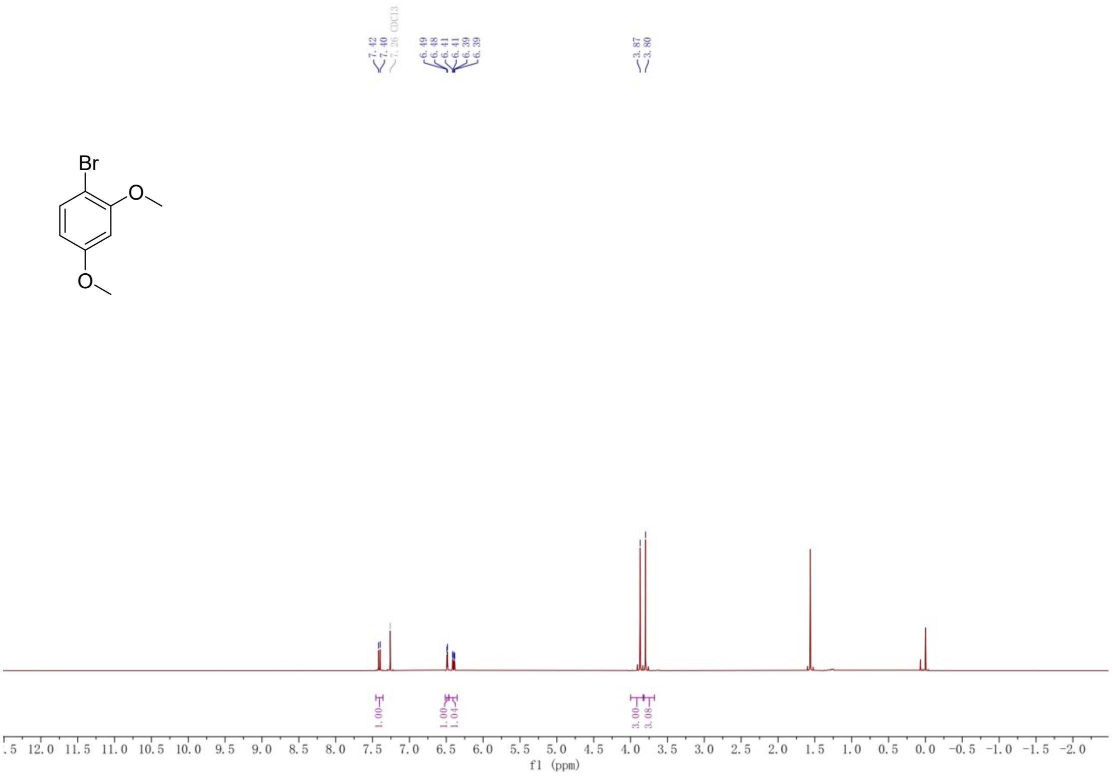

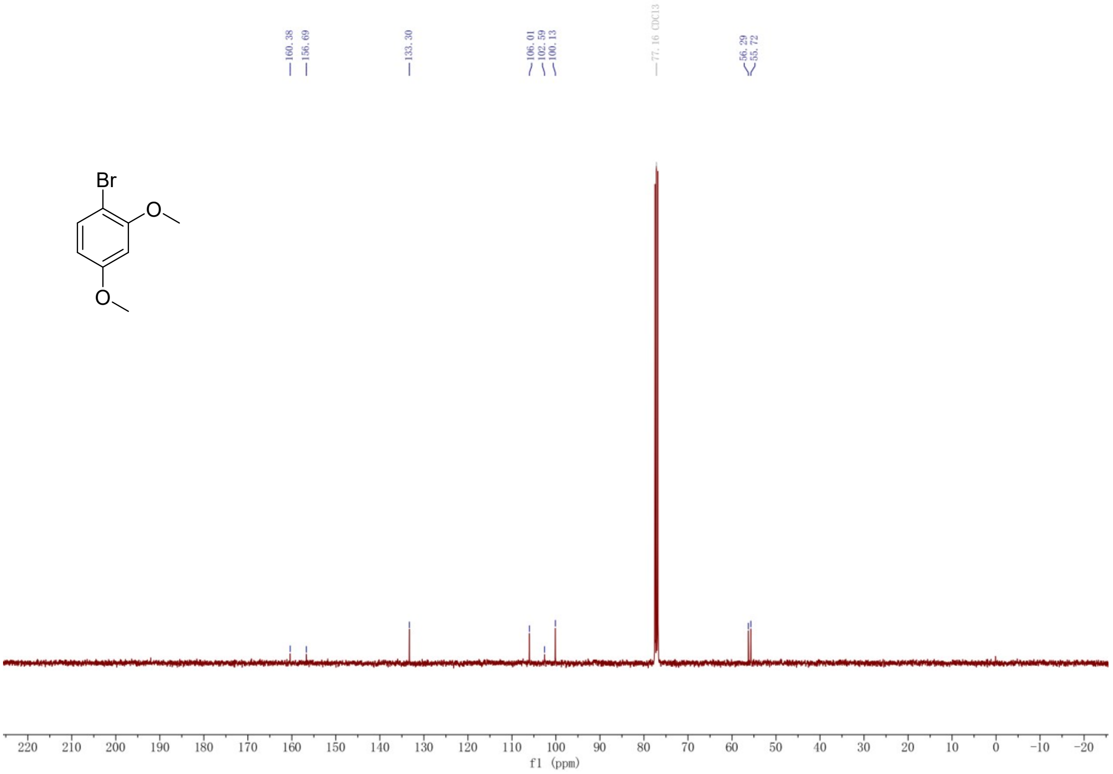

Figure S22. ^{1}H (top) and ^{13}C (bottom) NMR spectra of 1-bromo-2,4-dimethoxybenzene(table 2-23).

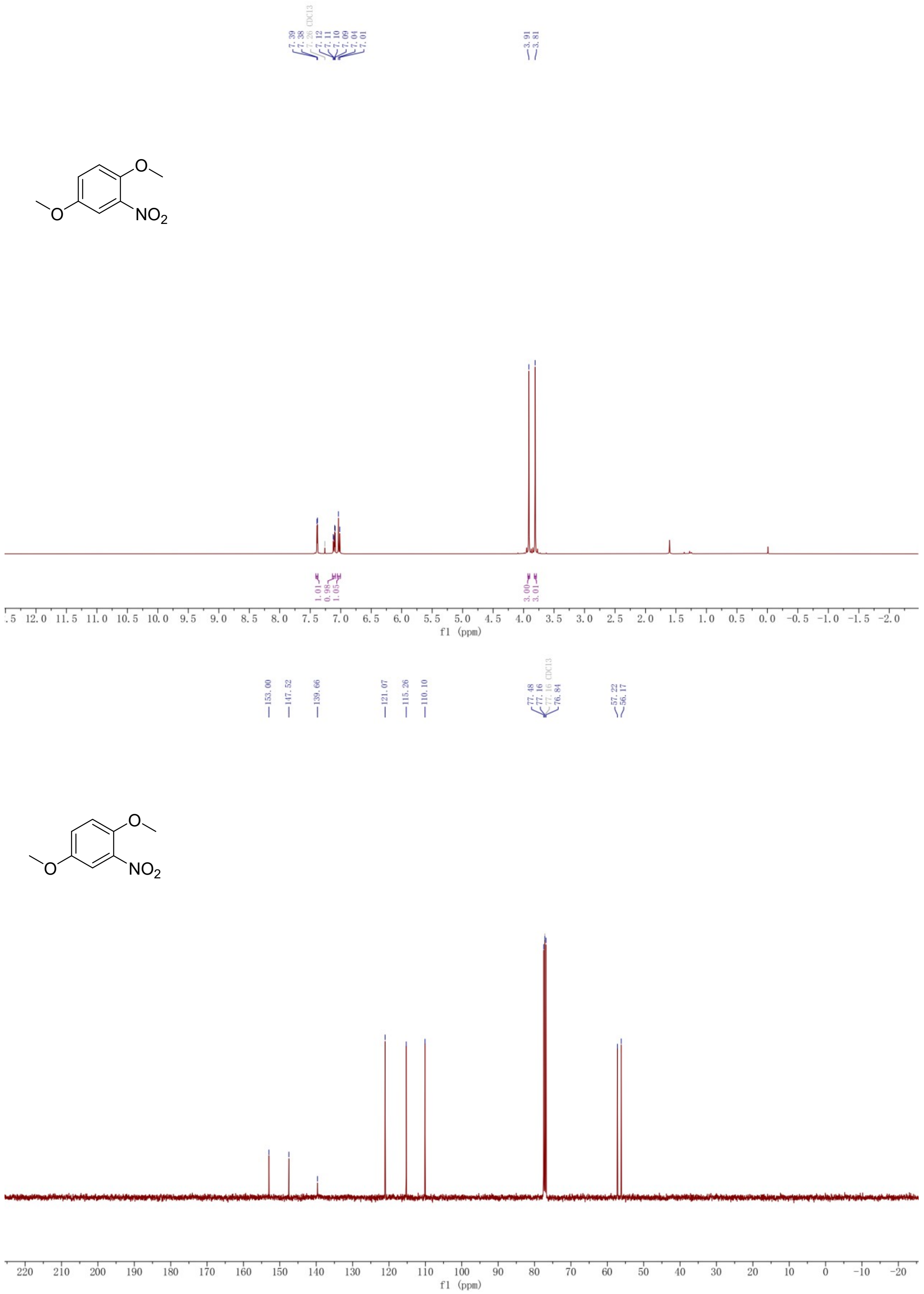

Figure S23. ^{1}H (top) and ^{13}C (bottom) NMR spectra of 1,4-dimethoxy-2-nitrobenzene(table 2-24 and table 3-20).

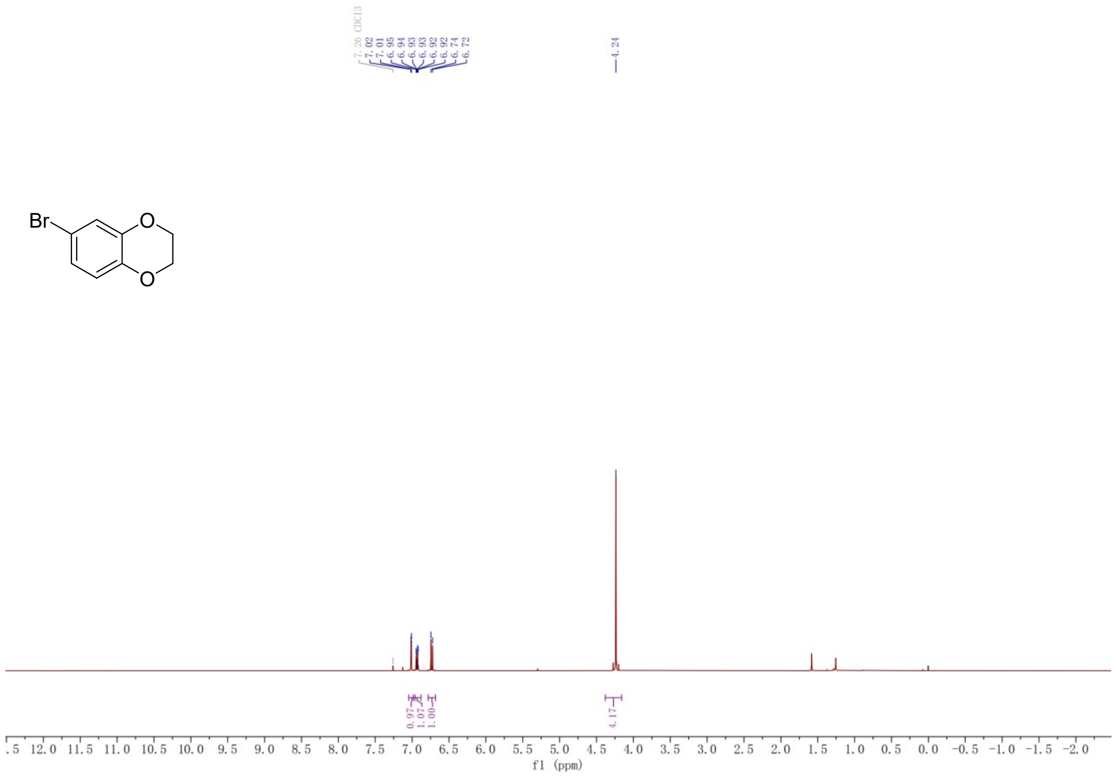

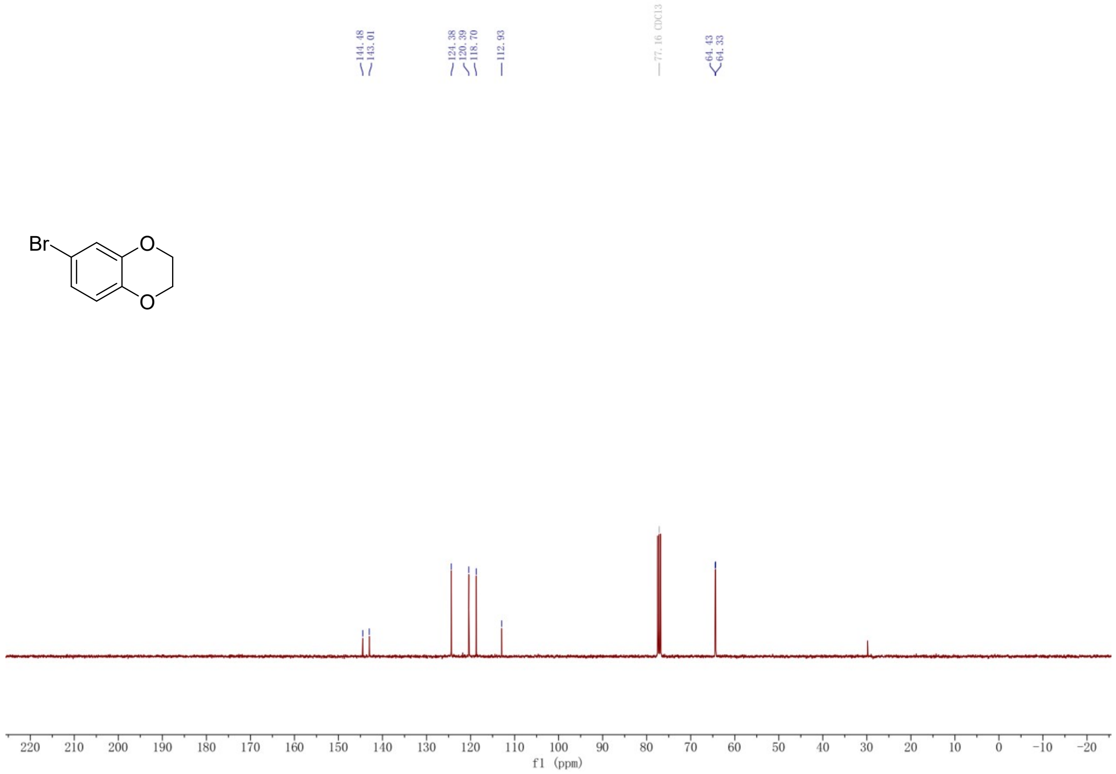

Figure S24. ^{1}H (top) and ^{13}C (bottom) NMR spectra of 6-bromo-2,3-dihydrobenzo[b][1,4]dioxine(table 2-25).

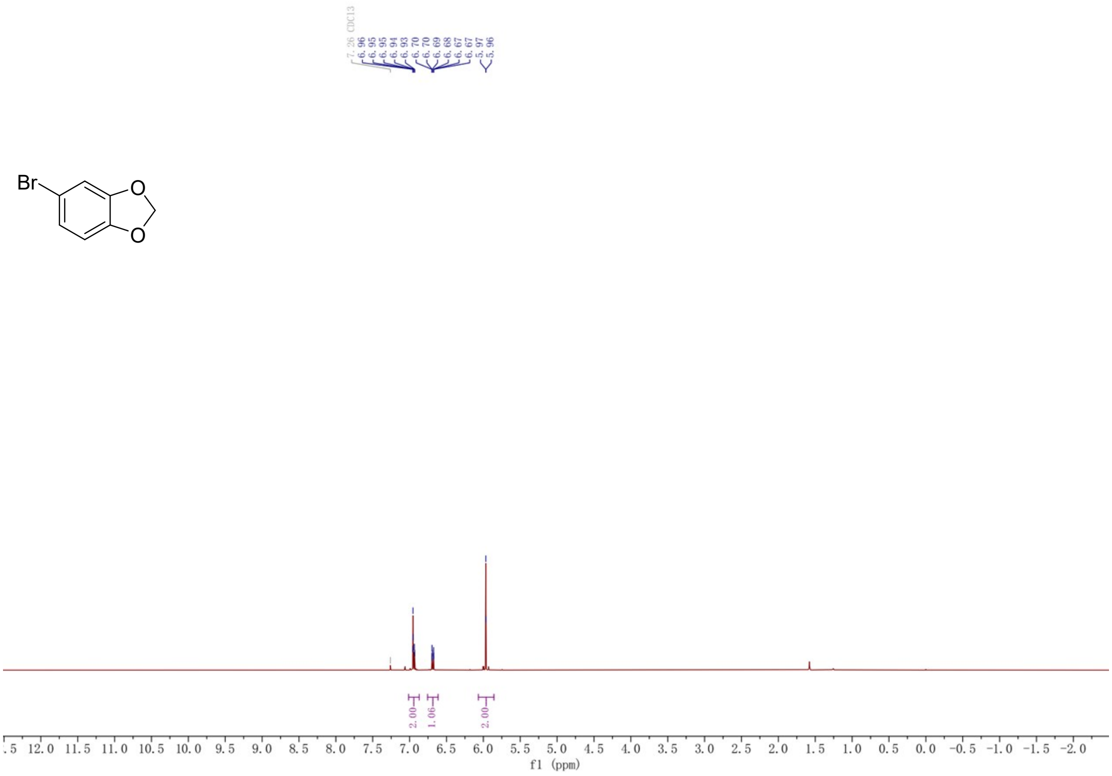

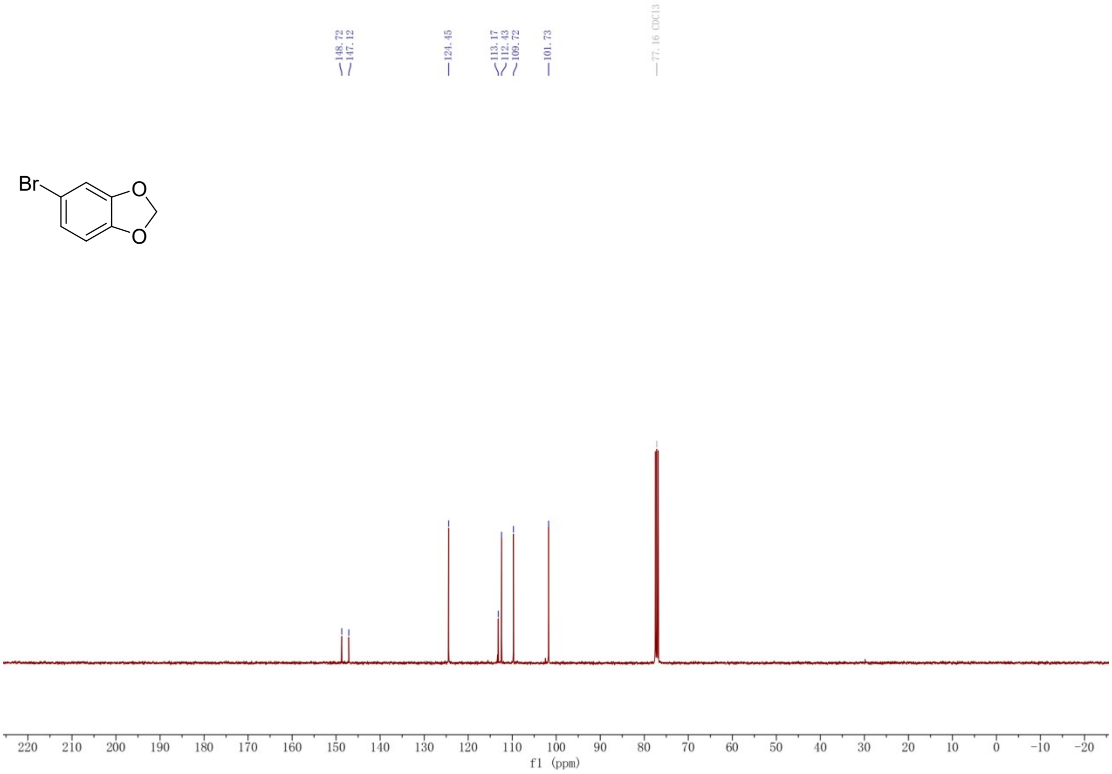

Figure S25. ^{1}H (top) and ^{13}C (bottom) NMR spectra of 5-bromobenzo[d][1,3]dioxole(table 2-26).

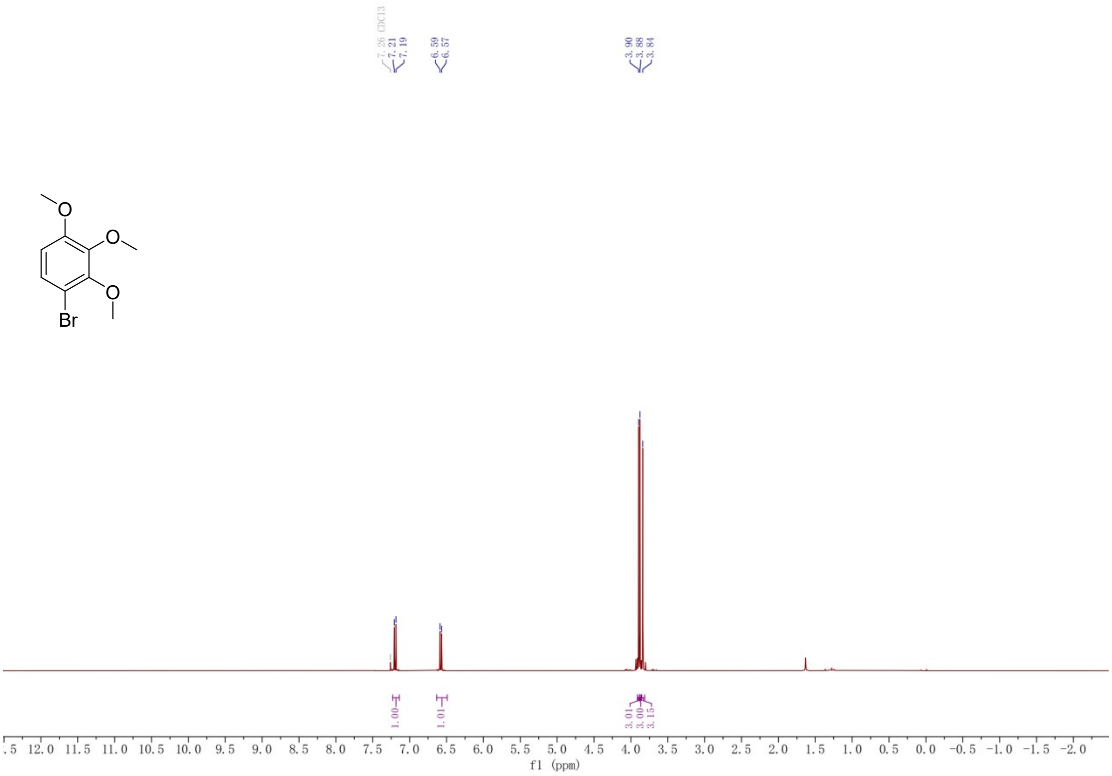

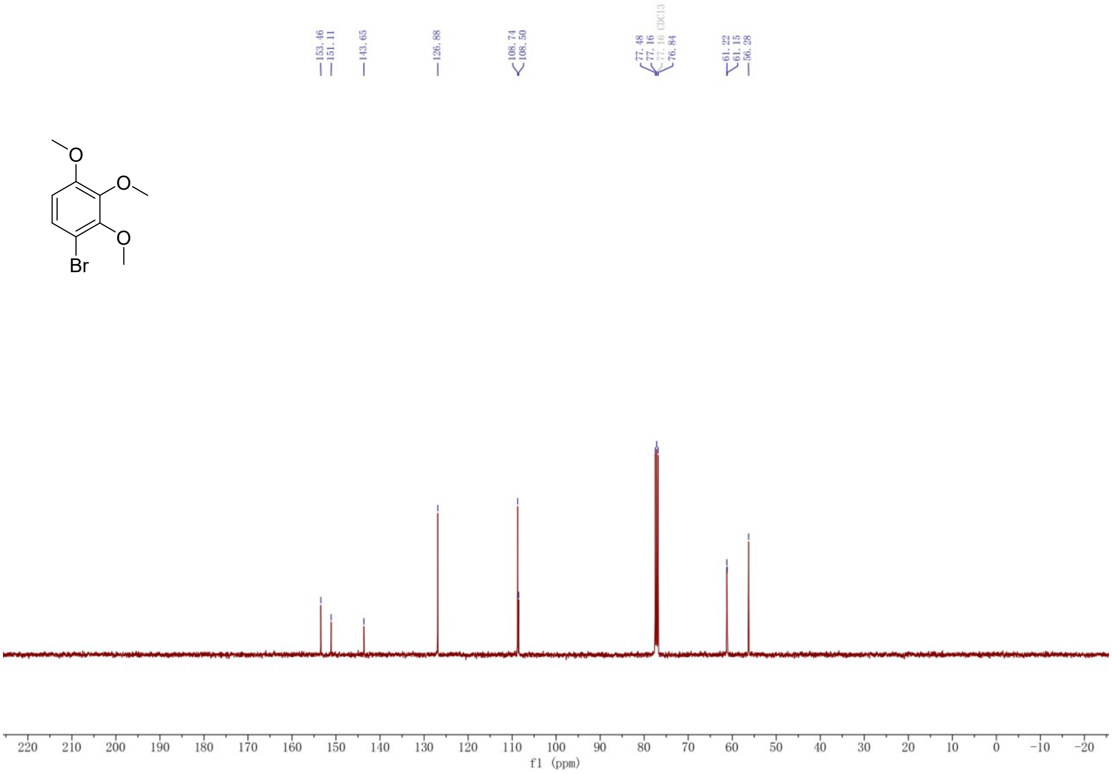

Figure S26. ^{1}H (top) and ^{13}C (bottom) NMR spectra of 1-bromo-2,3,4-trimethoxybenzene(table 2-27).

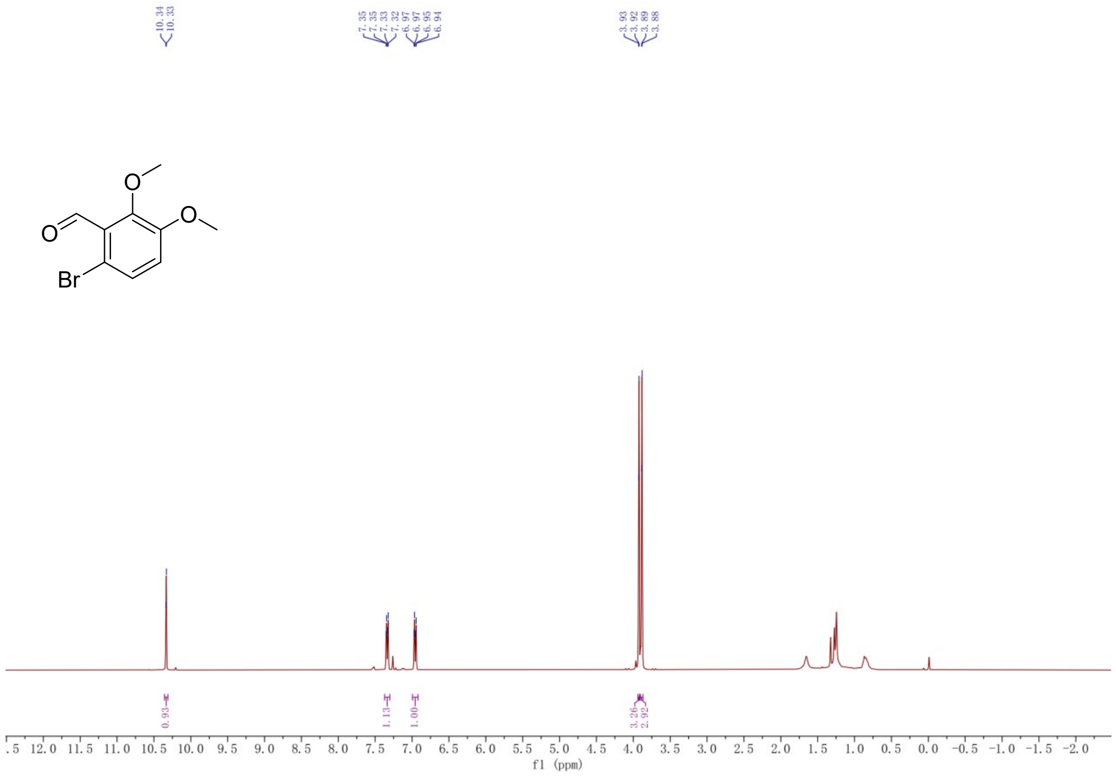

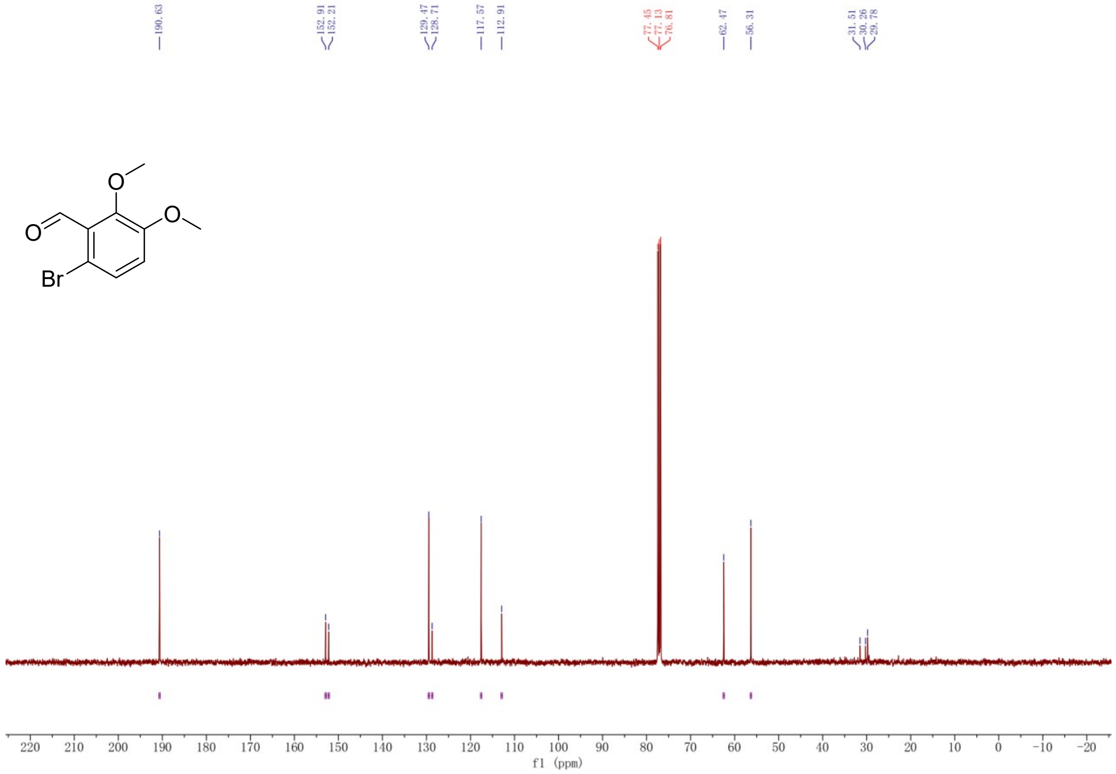

Figure S27. ^{1}H **(top) and** ^{13}C **(bottom) NMR spectra of 6-bromo-2,3-dimethoxybenzaldehyde(table 2-28).**

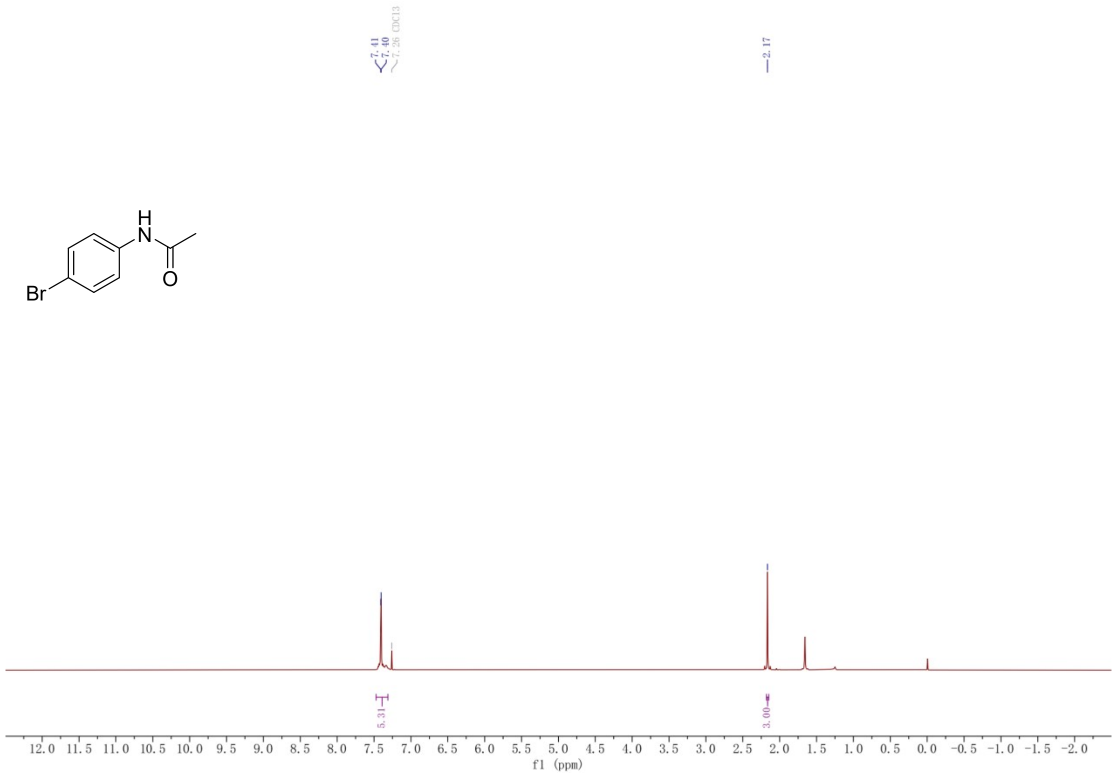

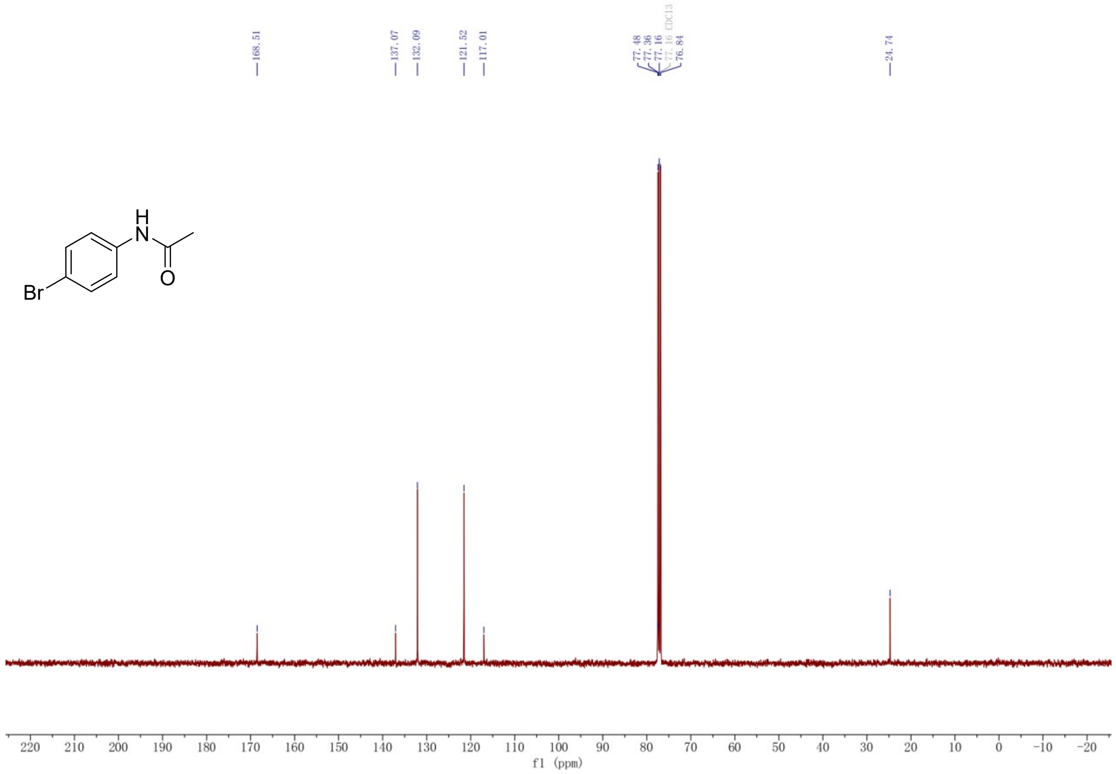

Figure S28. ^{1}H (top) and ^{13}C (bottom) NMR spectra of N-(4-bromophenyl)acetamide(table 2-30).

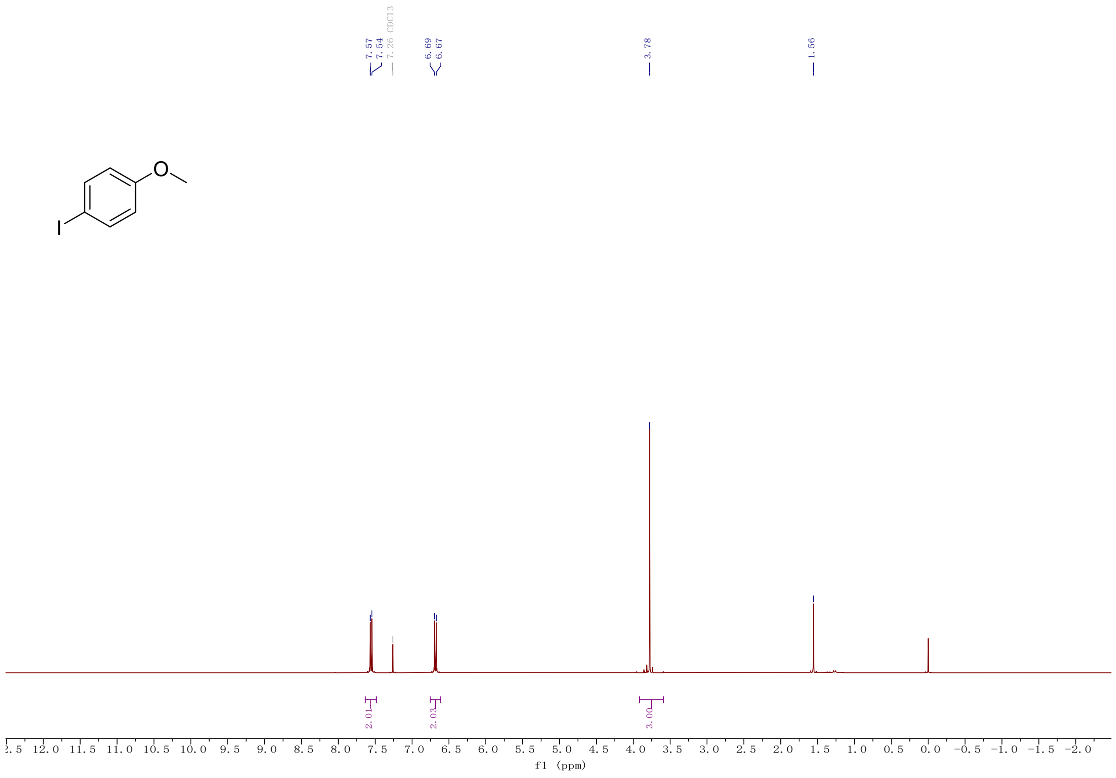

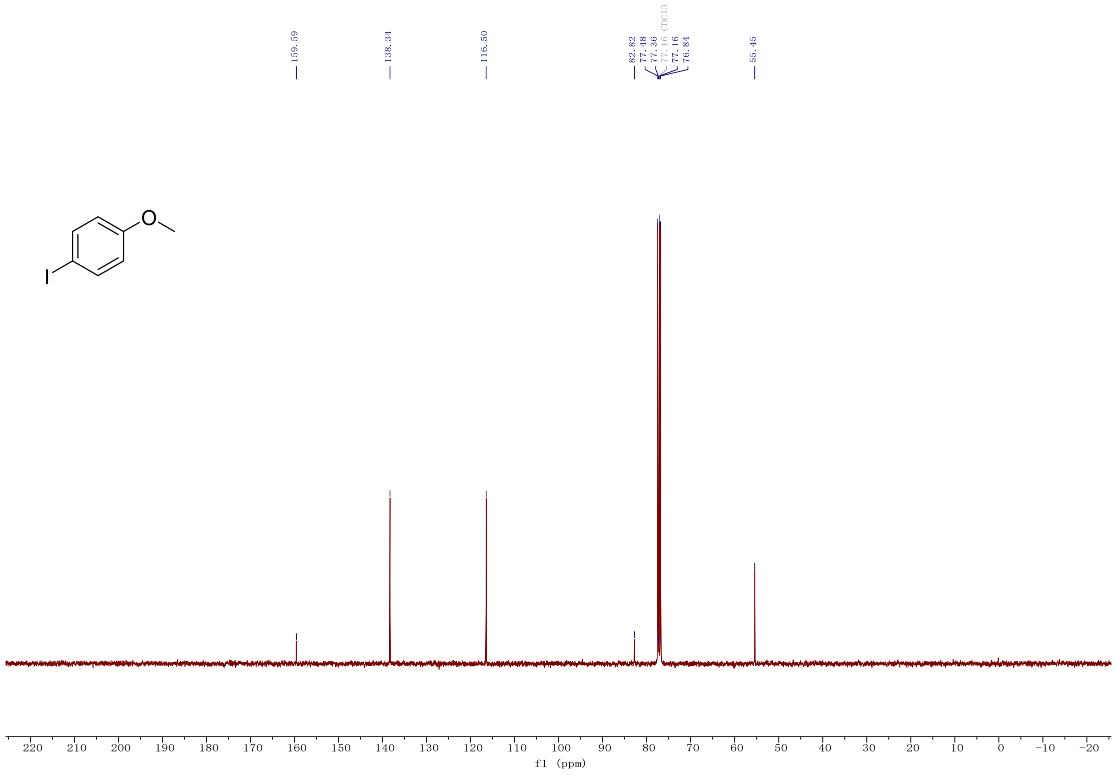

Figure S29. **^{1}H (top) and ^{13}C (bottom) NMR spectra of 1-iodo-4-methoxybenzene (table 3-1).**

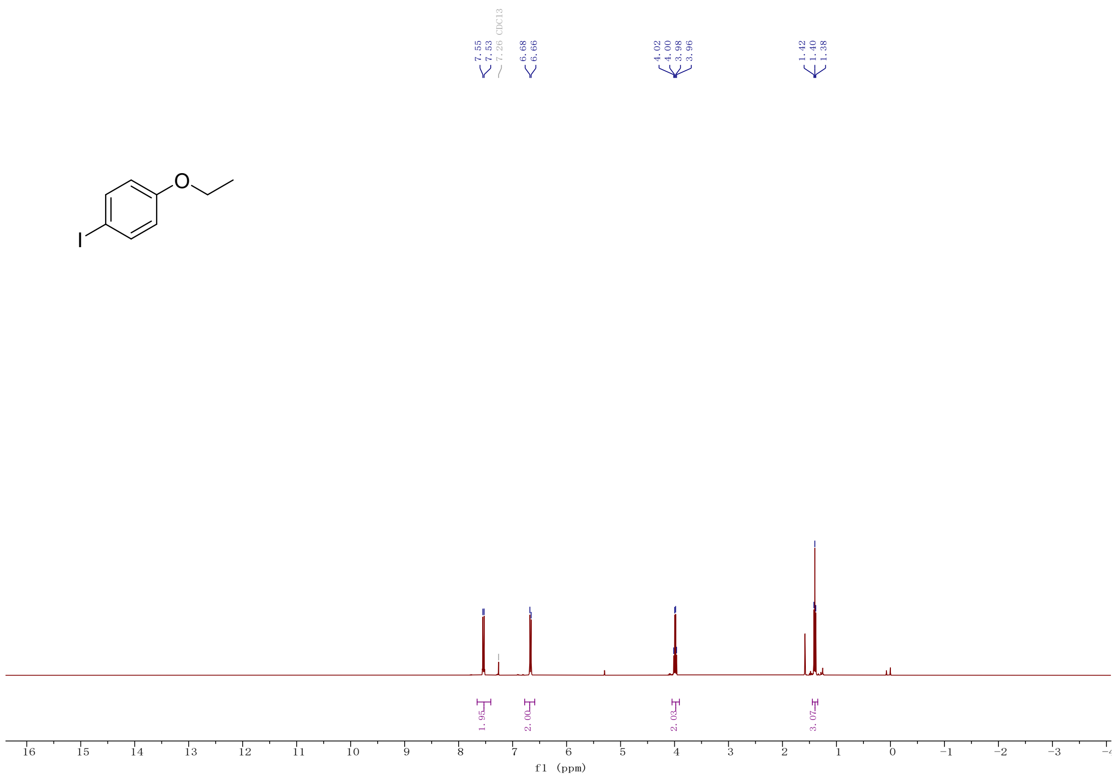

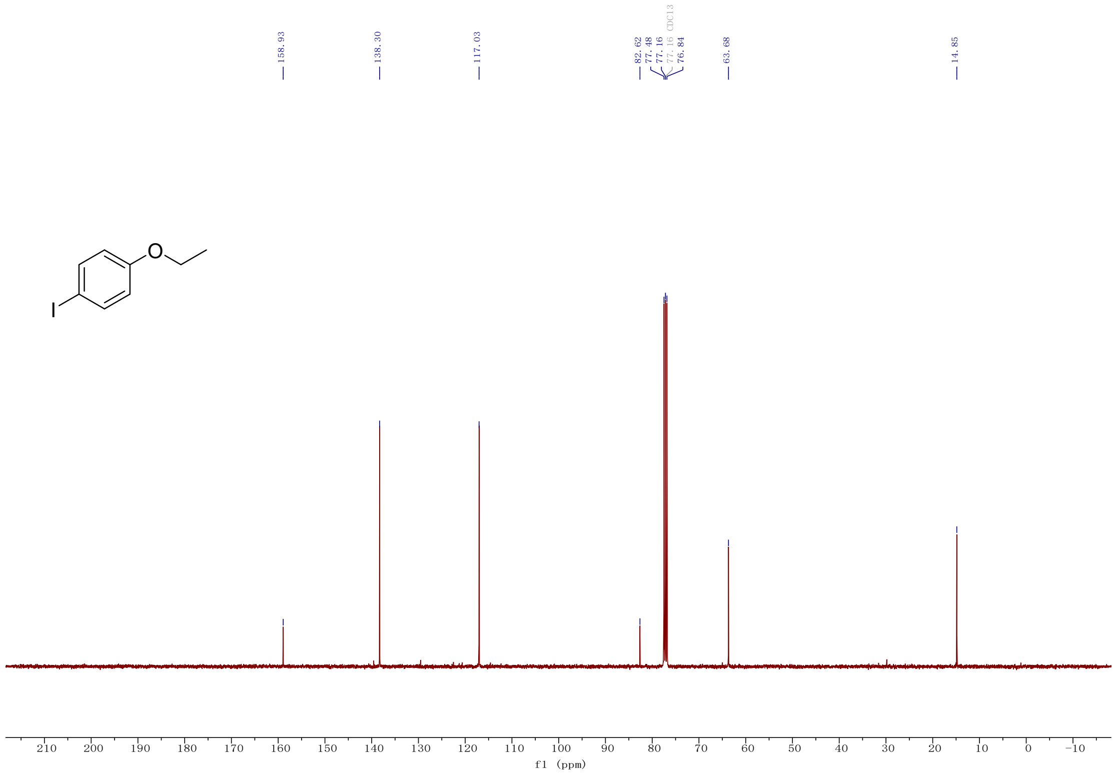

Figure S30. ^{1}H (top) and ^{13}C (bottom) NMR spectra of 1-ethoxy-4-iodobenzene (table 3-2).

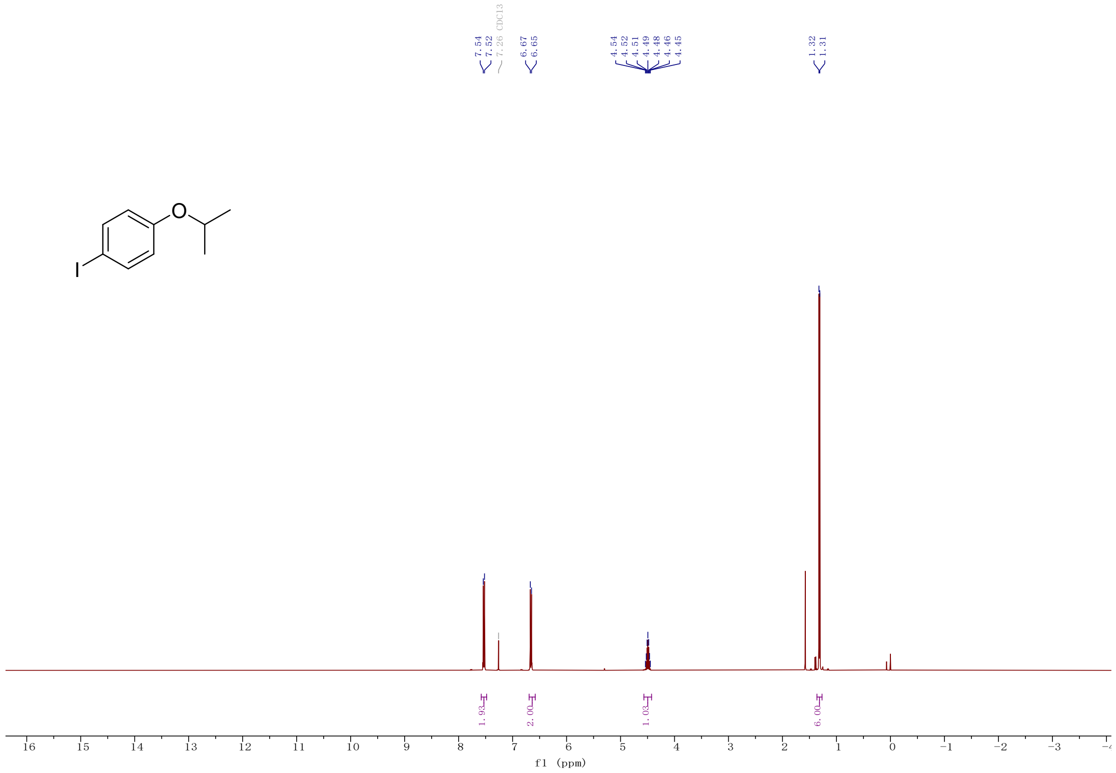

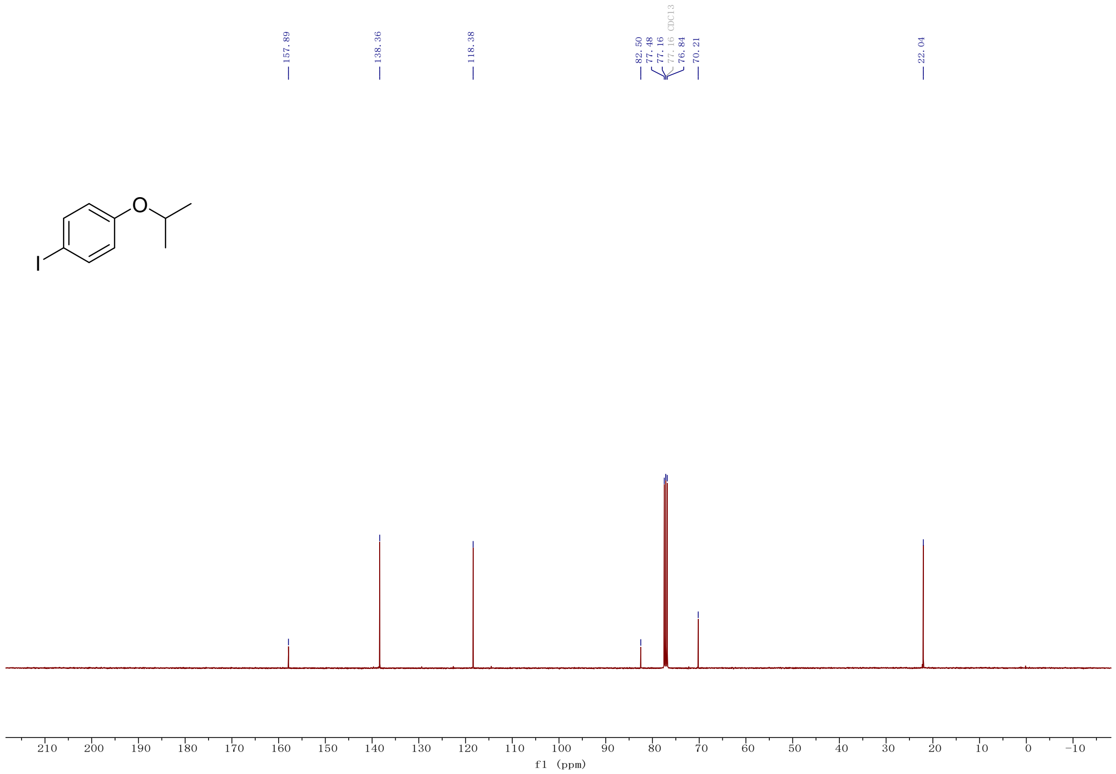

Figure S31. ^{1}H (top) and ^{13}C (bottom) NMR spectra of 1-iodo-4-isopropoxybenzene (table 3-3).

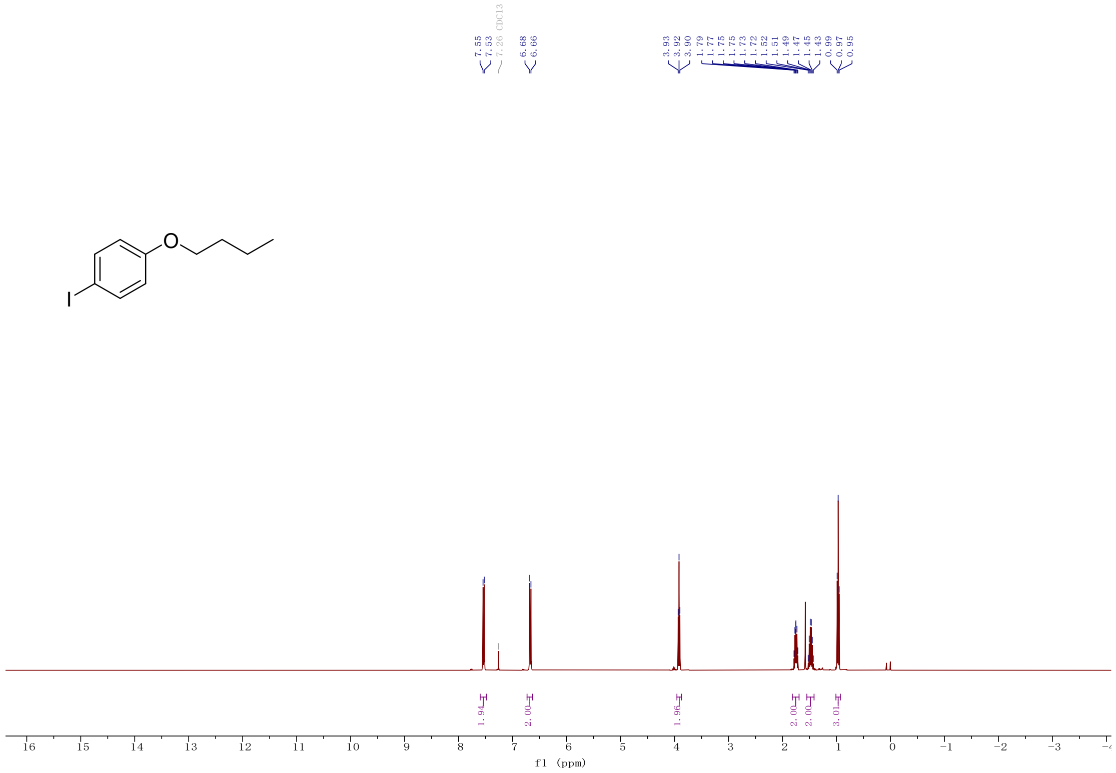

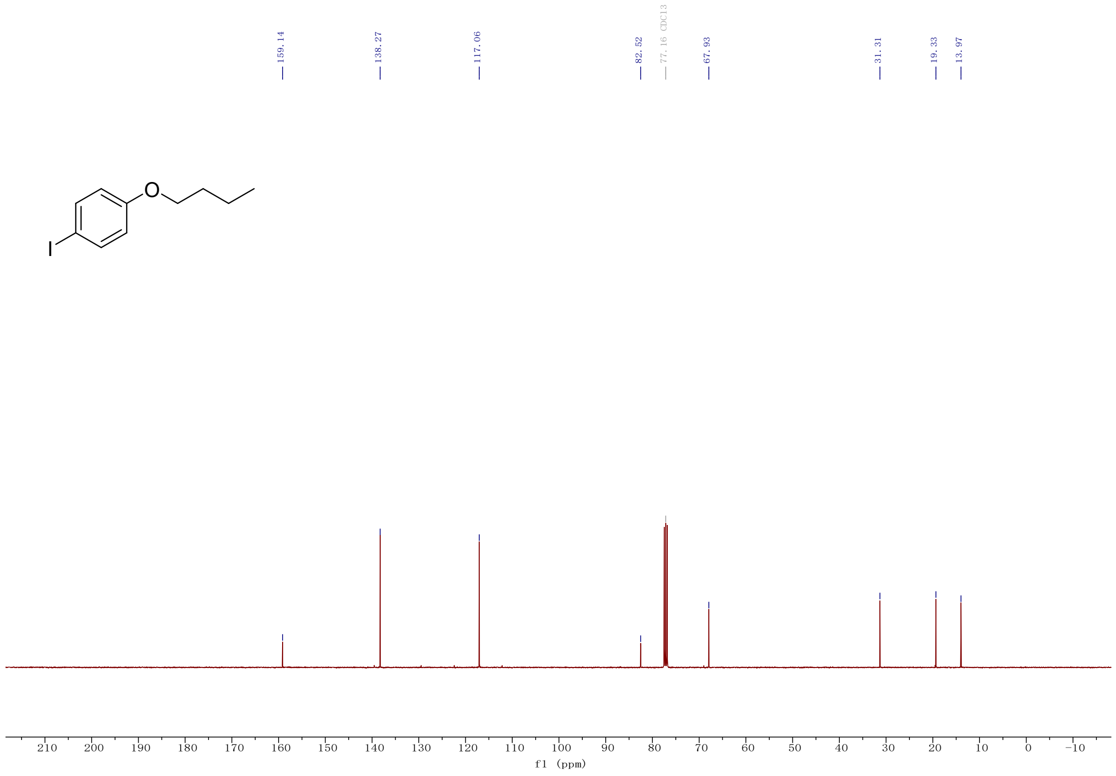

Figure S32. ^{1}H (top) and ^{13}C (bottom) NMR spectra of 1-butoxy-4-iodobenzene (table 3-5).

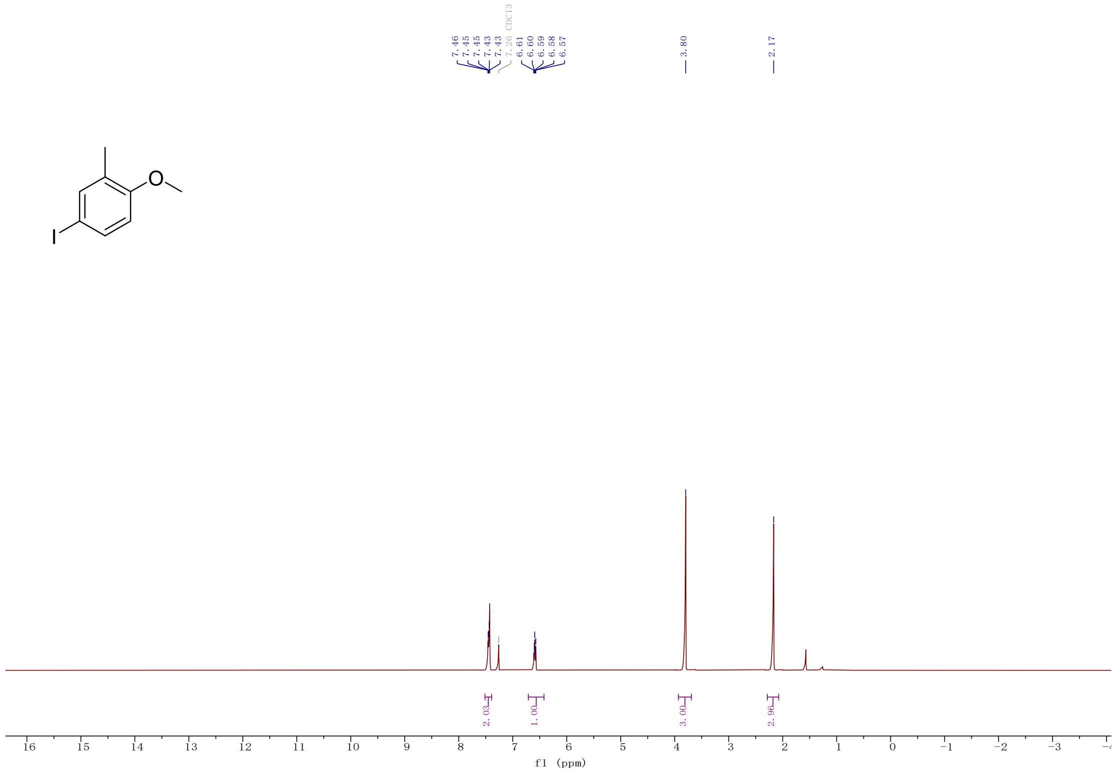

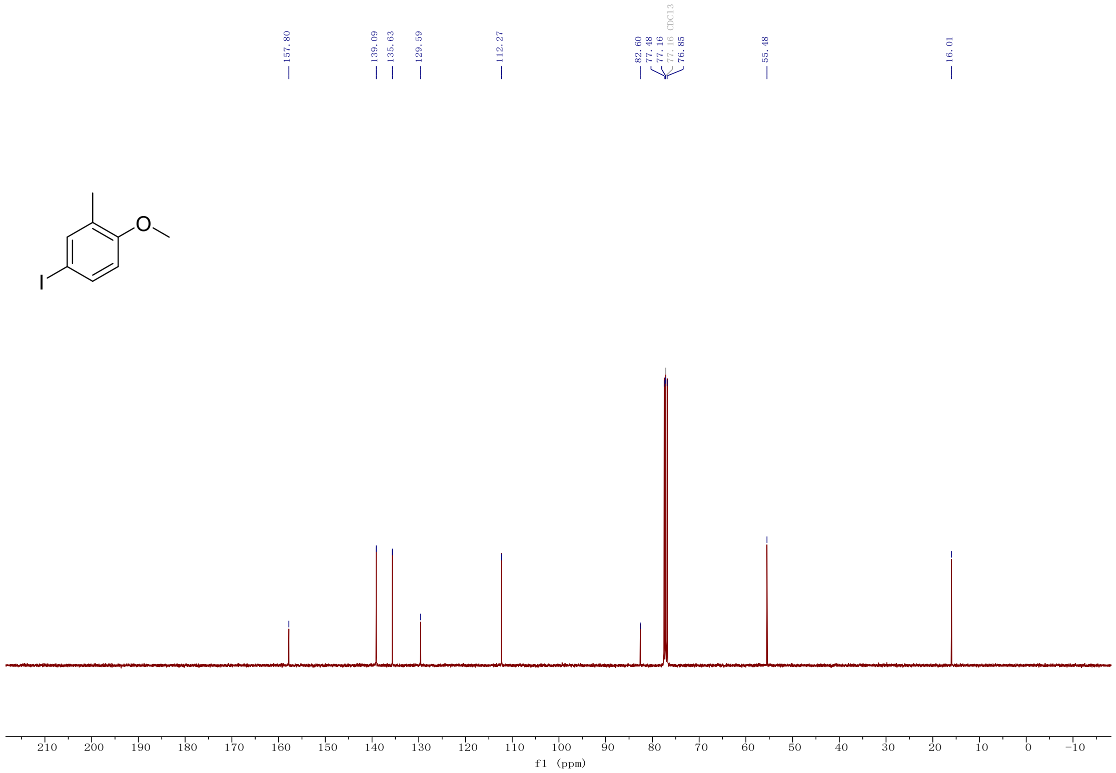

Figure S33. ^{1}H **(top) and** ^{13}C **(bottom) NMR spectra of 4-iodo-1-methoxy-2-methylbenzene (table 3-6).**

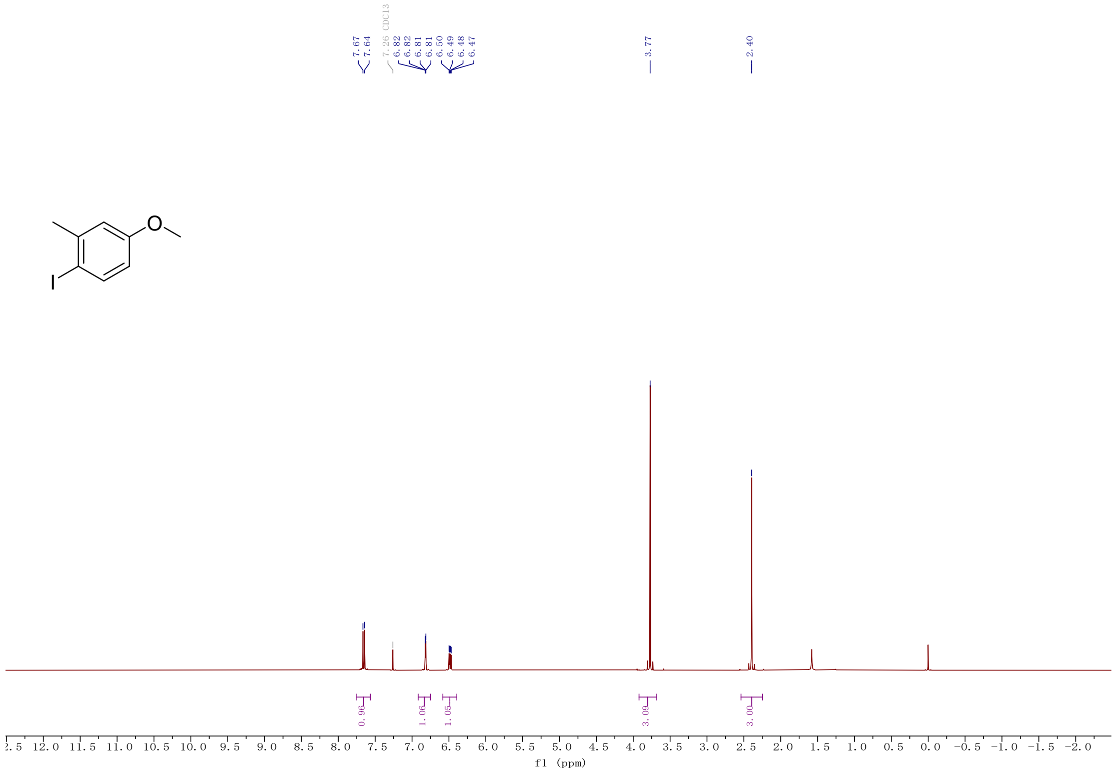

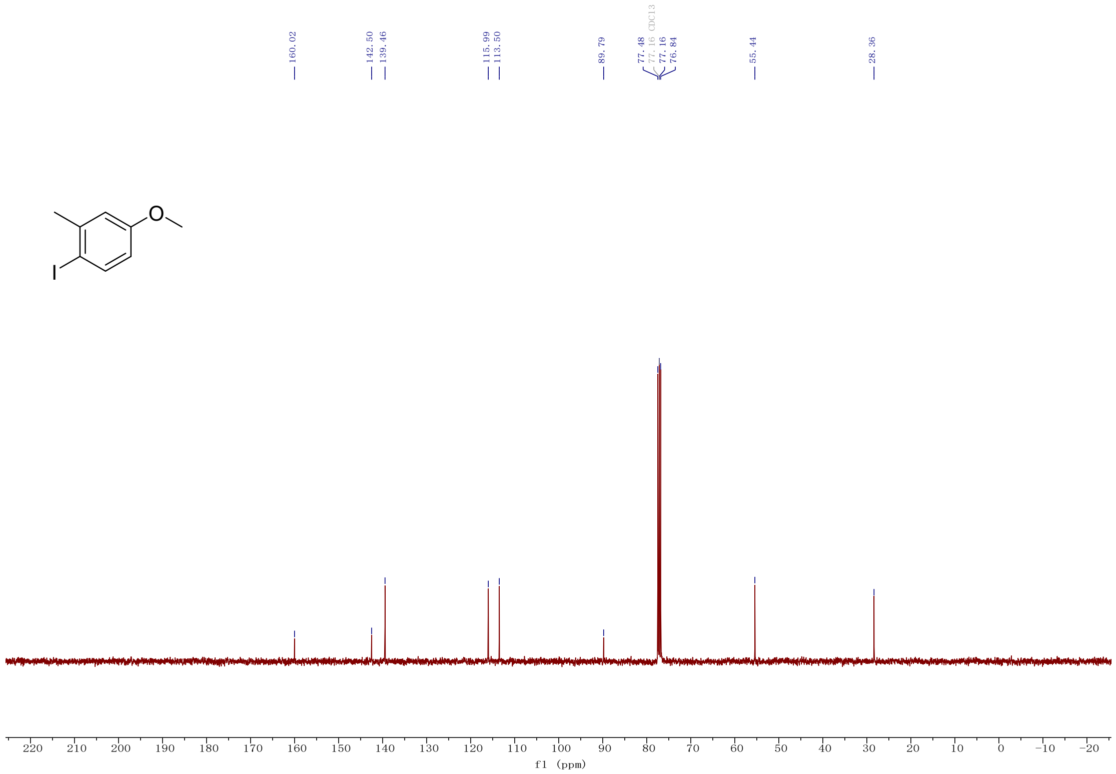

Figure S34. **^{1}H (top) and ^{13}C (bottom) NMR spectra of 1-iodo-4-methoxy-2-methylbenzene (table 3-7).**

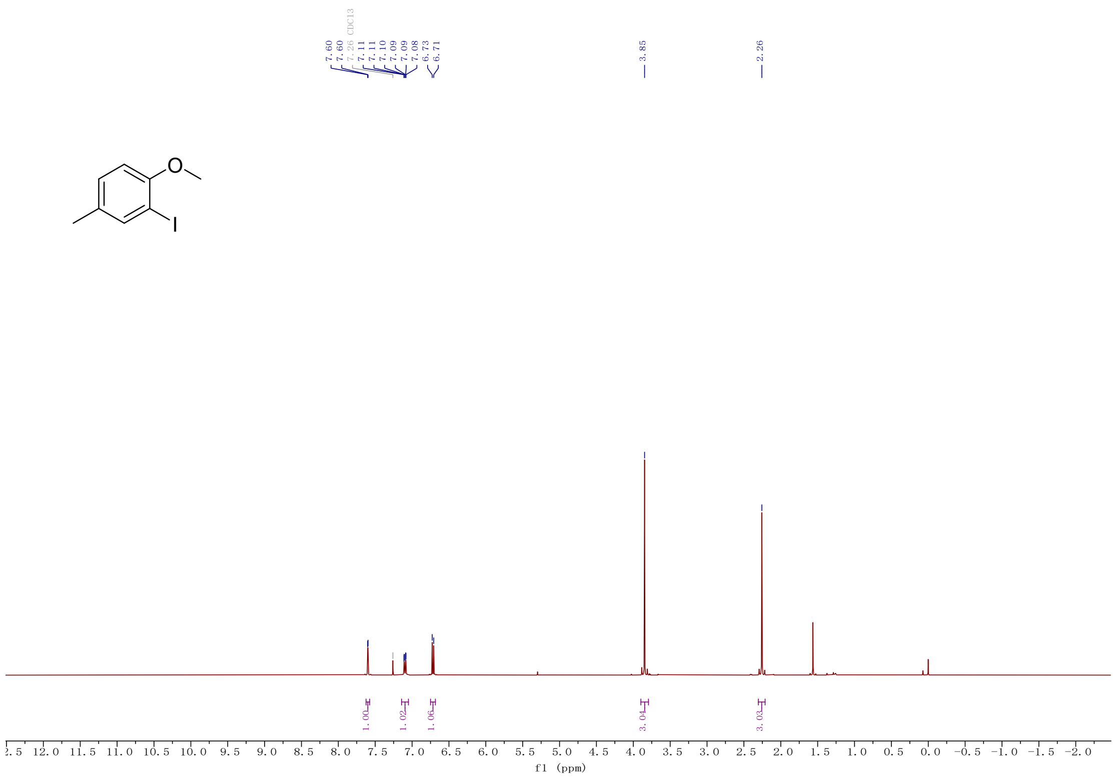

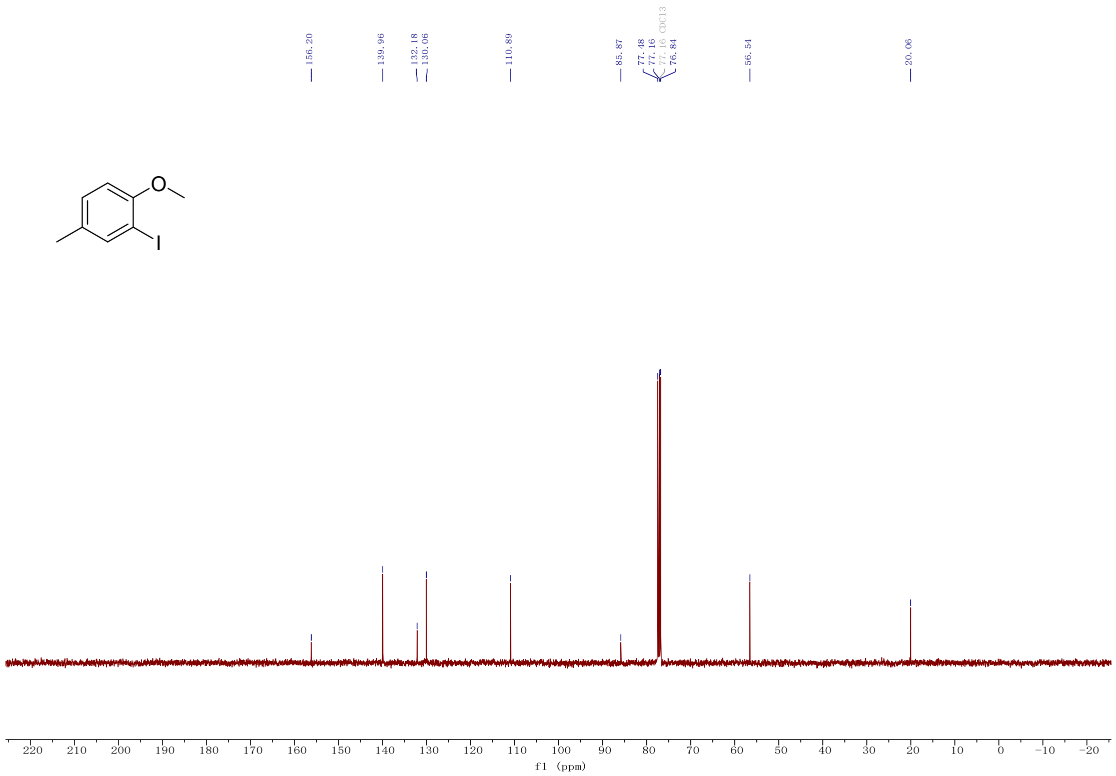

Figure S35. **^{1}H (top) and ^{13}C (bottom) NMR spectra of 2-iodo-1-methoxy-4-methylbenzene (table 3-8).**

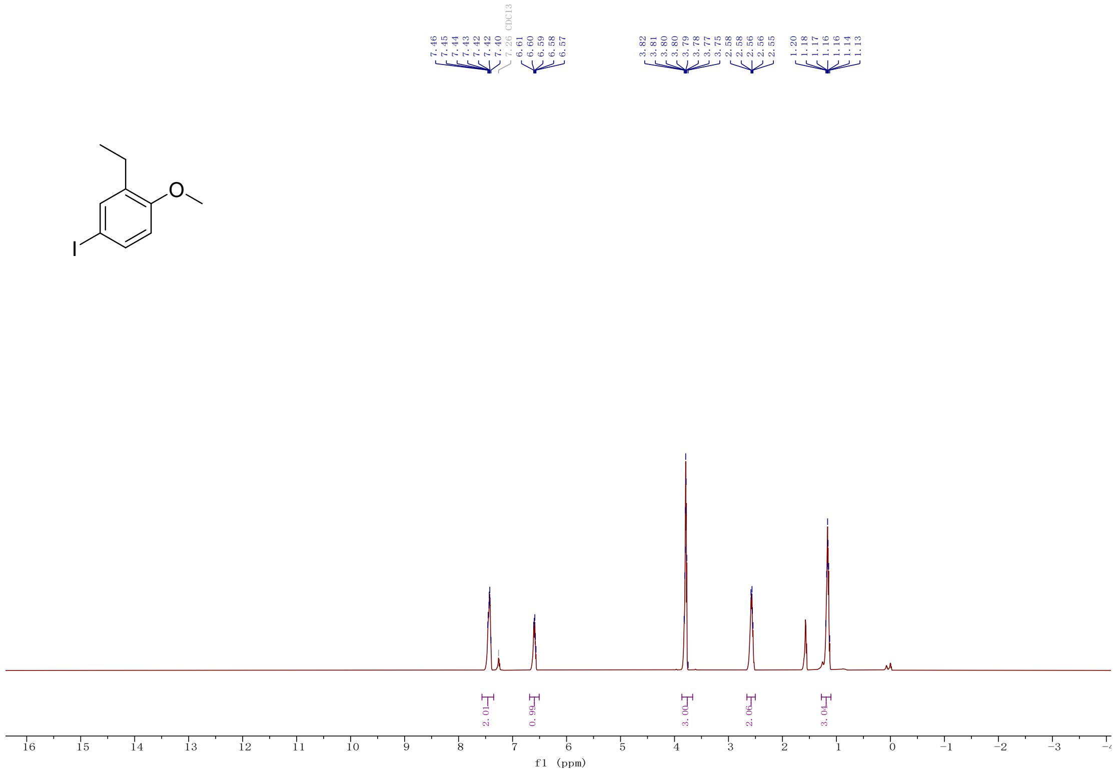

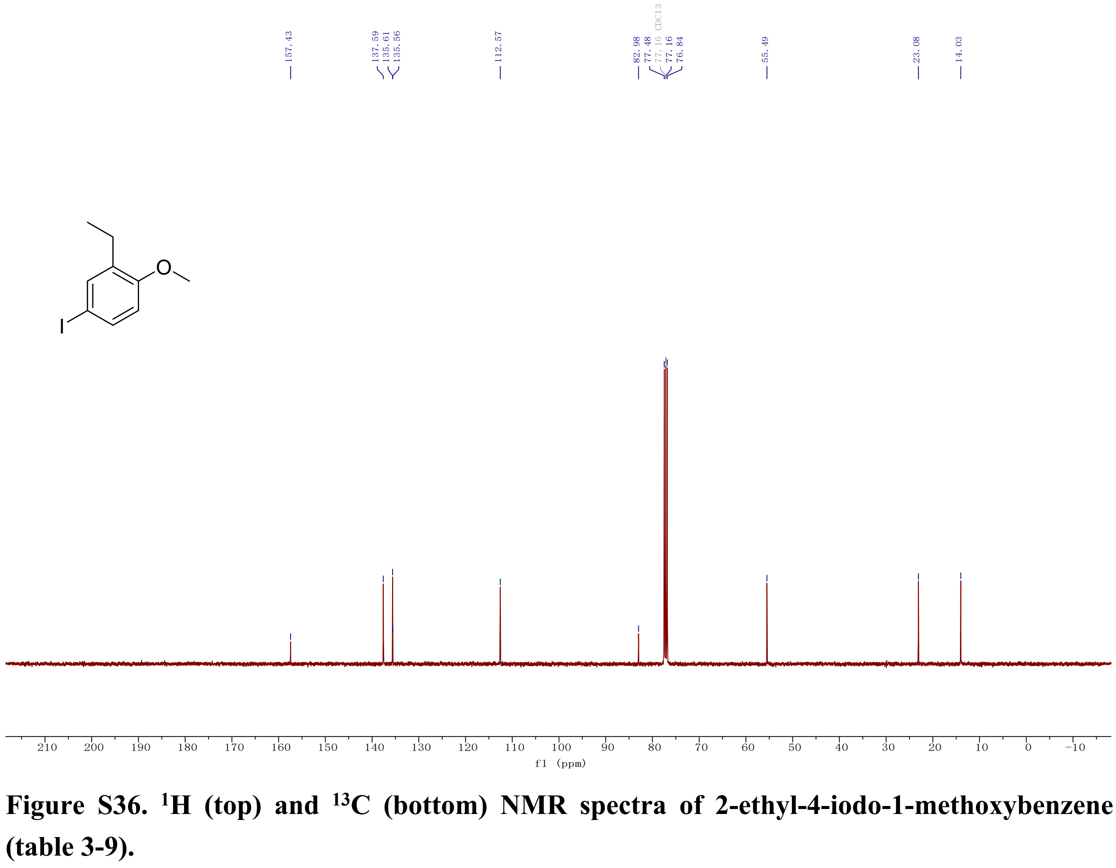

Figure S36. ^{1}H (top) and ^{13}C (bottom) NMR spectra of 2-ethyl-4-iodo-1-methoxybenzene (table 3-9).

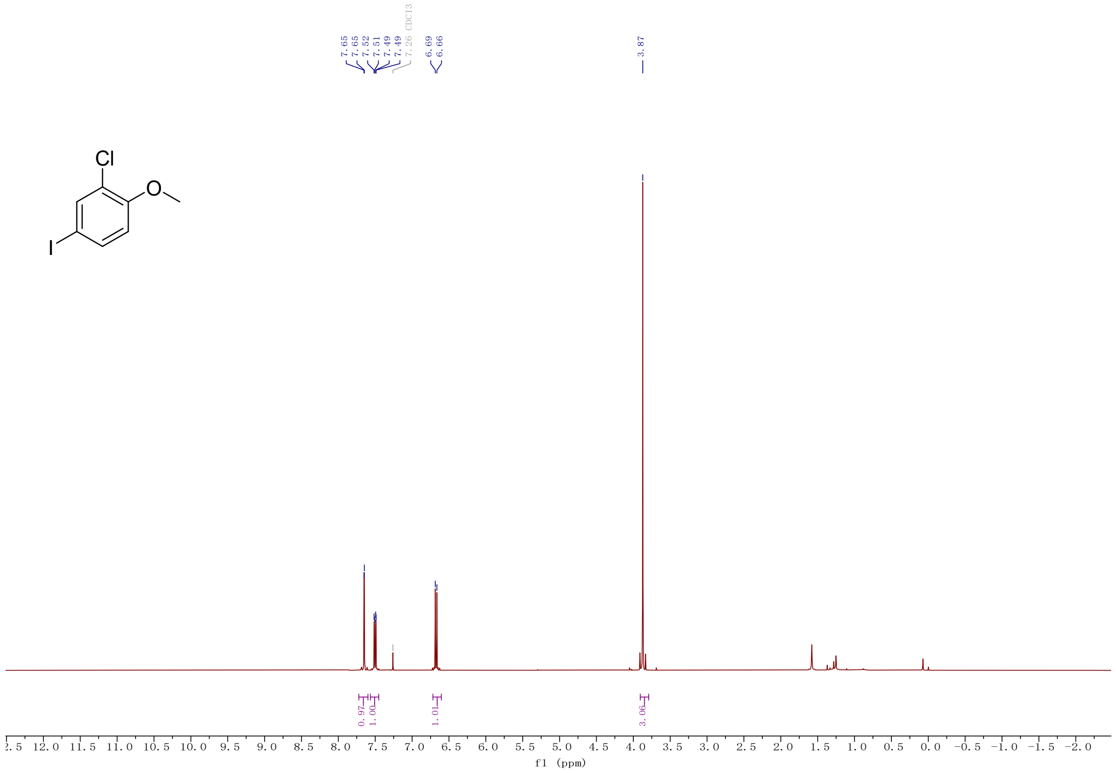

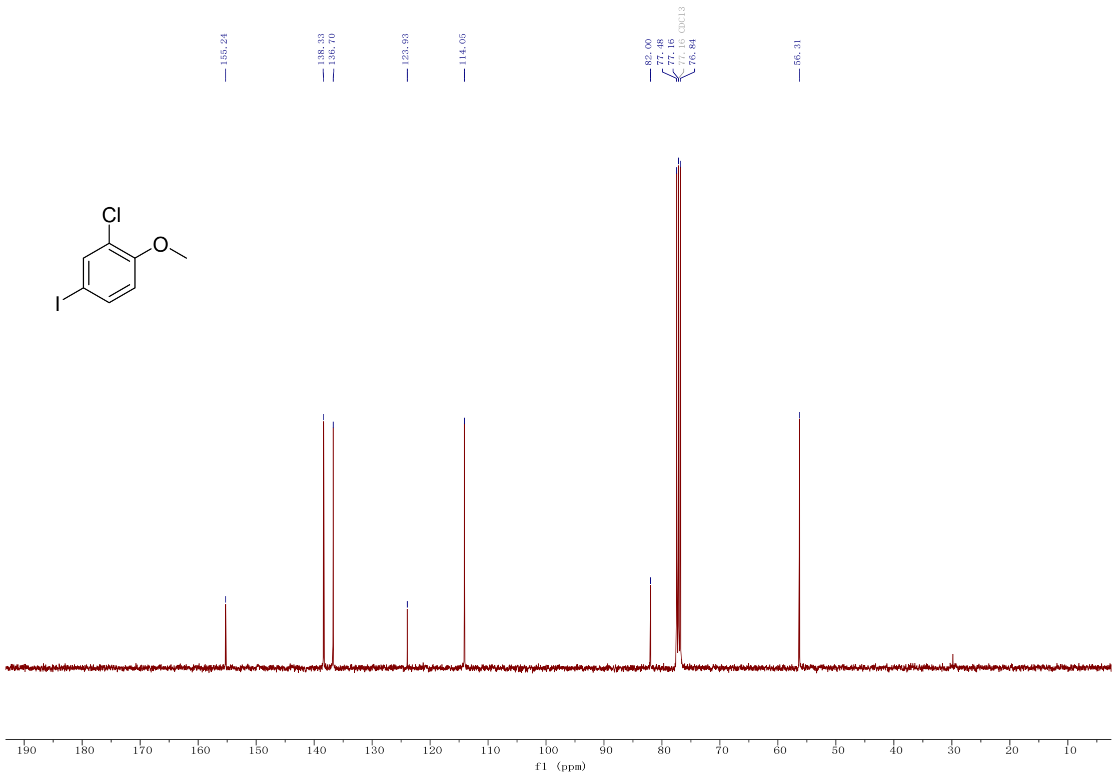

Figure S37. ^{1}H (top) and ^{13}C (bottom) NMR spectra of 2-chloro-4-iodo-1-methoxybenzene (table 3-10).

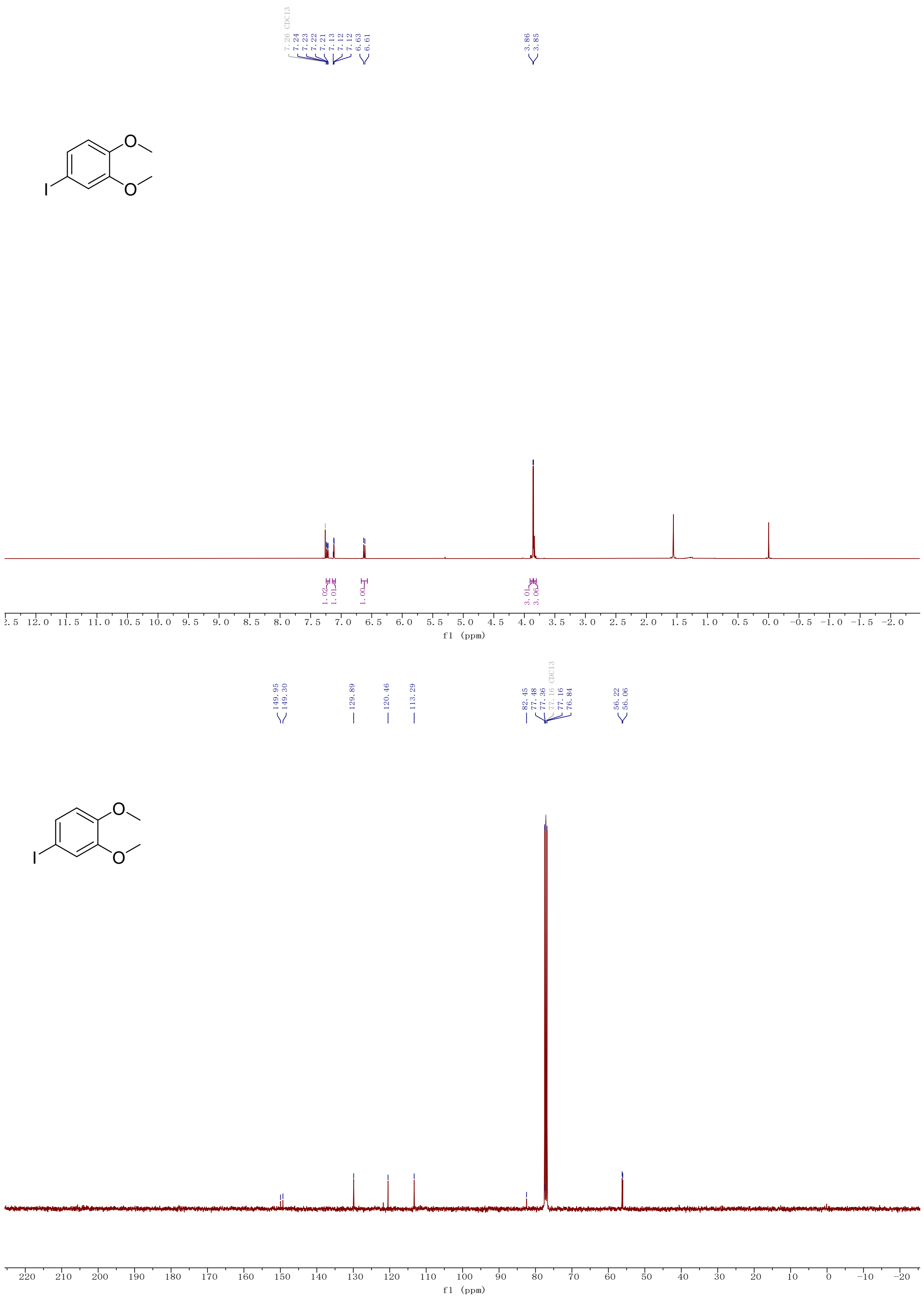

Figure S38. ^{1}H (top) and ^{13}C (bottom) NMR spectra of 4-iodo-1,2-dimethoxybenzene (table 3-18-1).

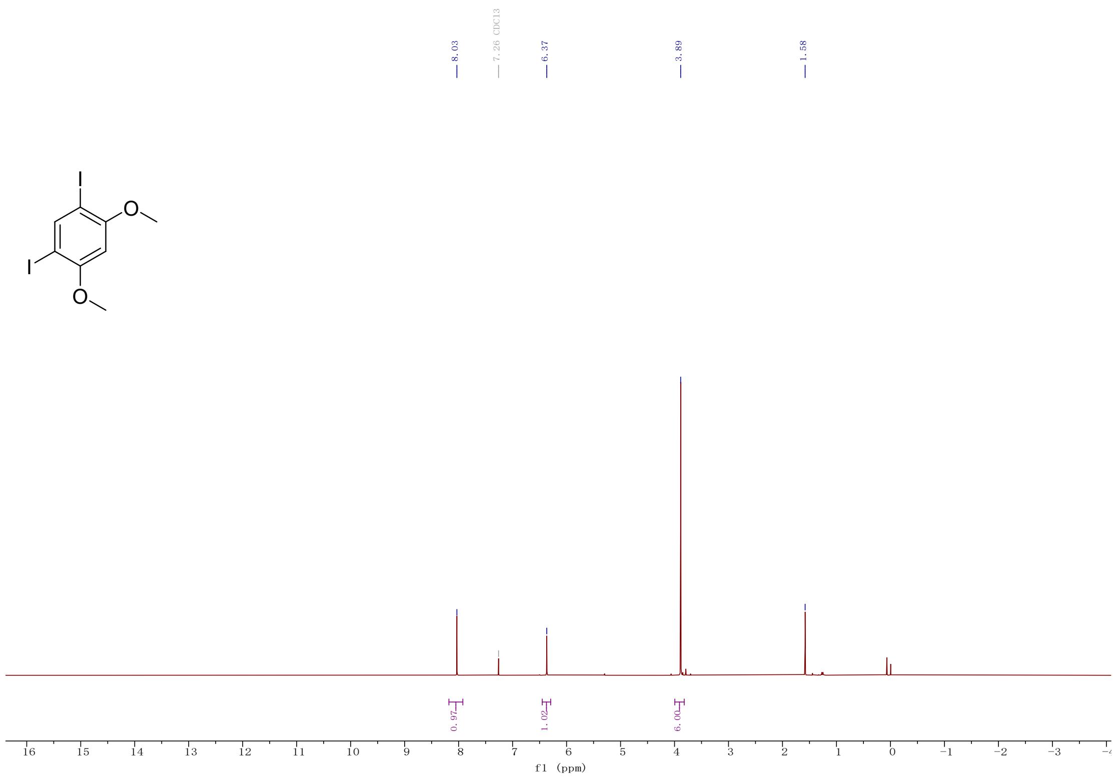

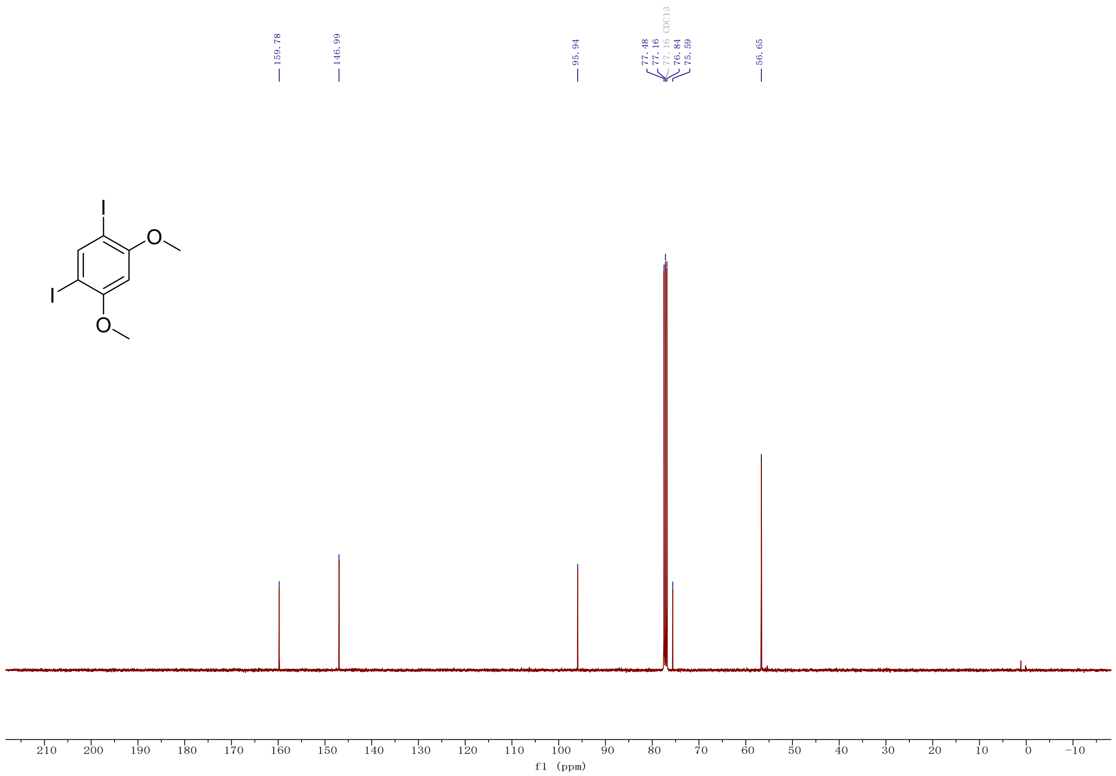

Figure S39. ^{1}H (top) and ^{13}C (bottom) NMR spectra of 1,5-diiodo-2,4-dimethoxybenzene (table 3-19).

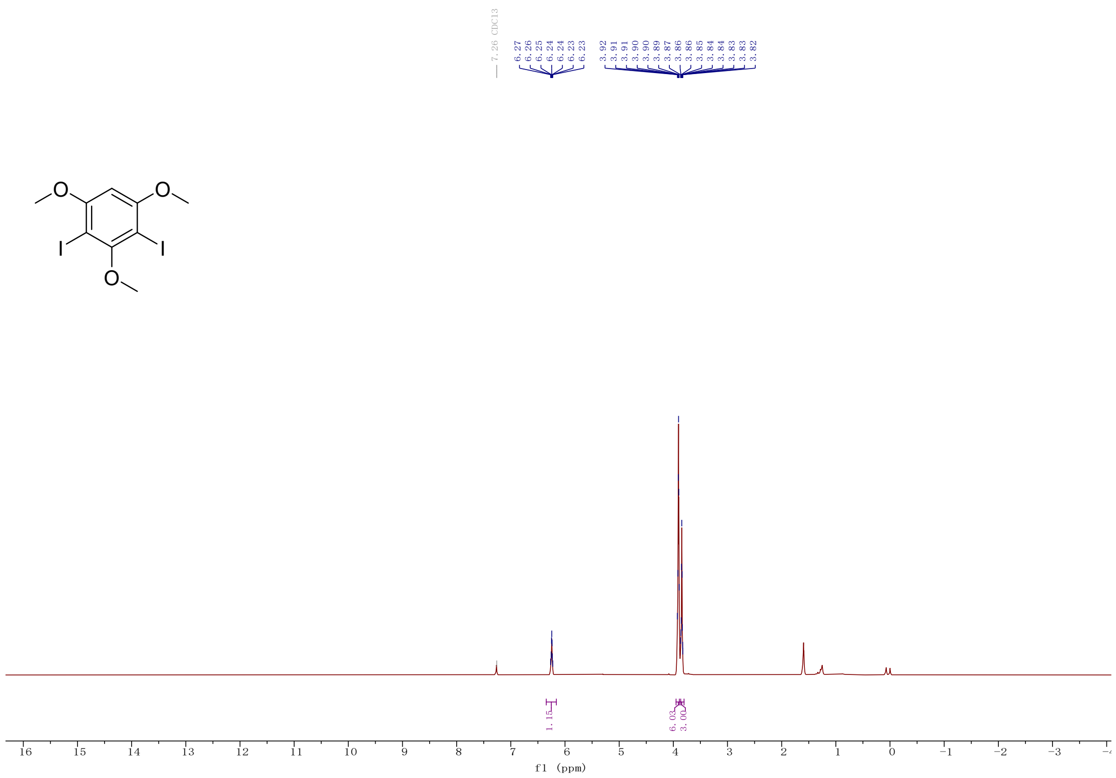

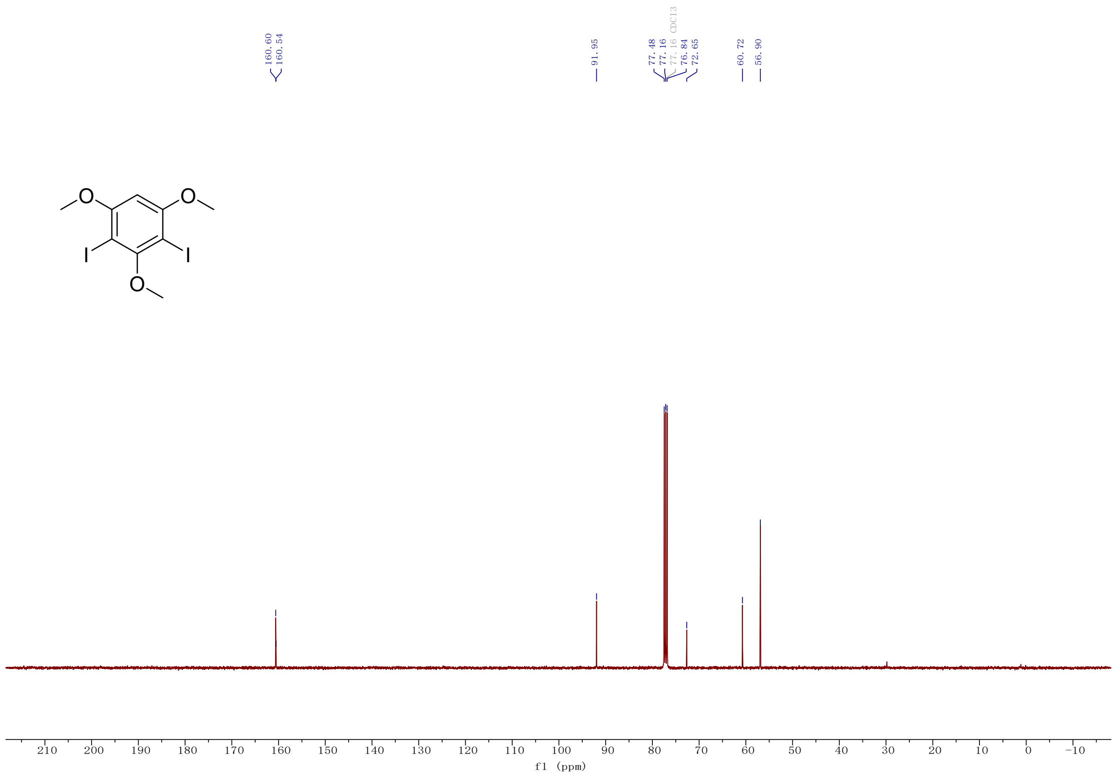

Figure S39. ^{1}H (top) and ^{13}C (bottom) NMR spectra of 2,4-diiodo-1,3,5-trimethoxybenzene (table 3-21).

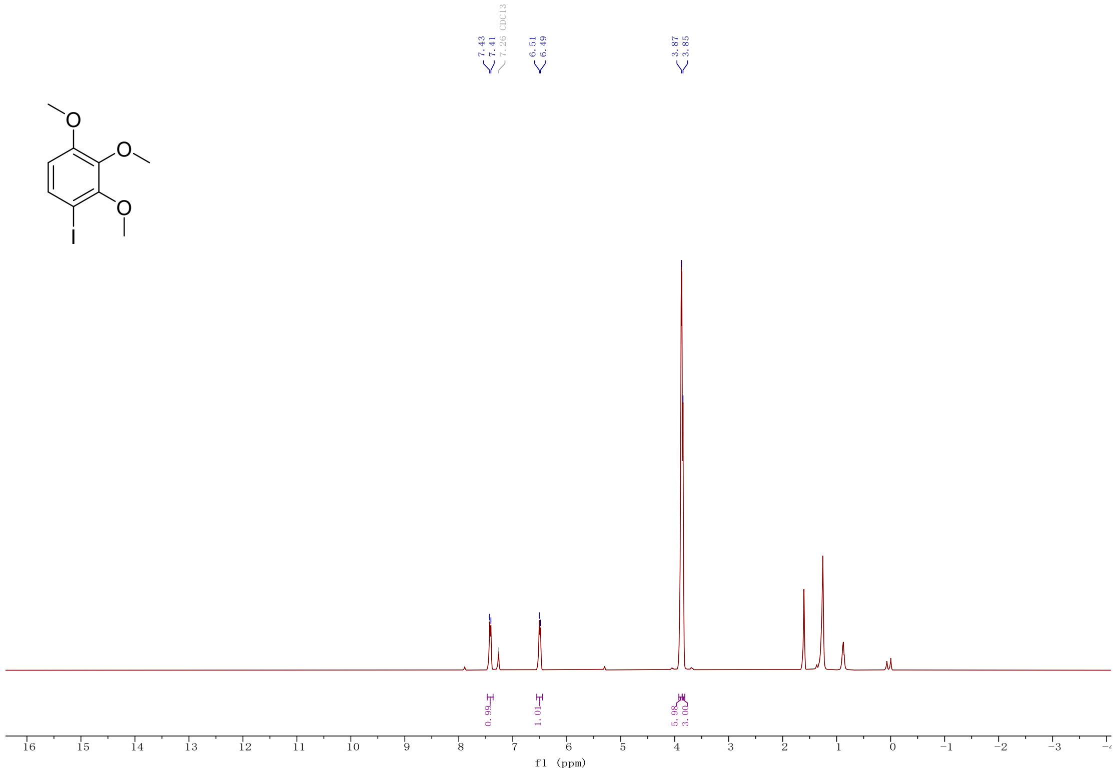

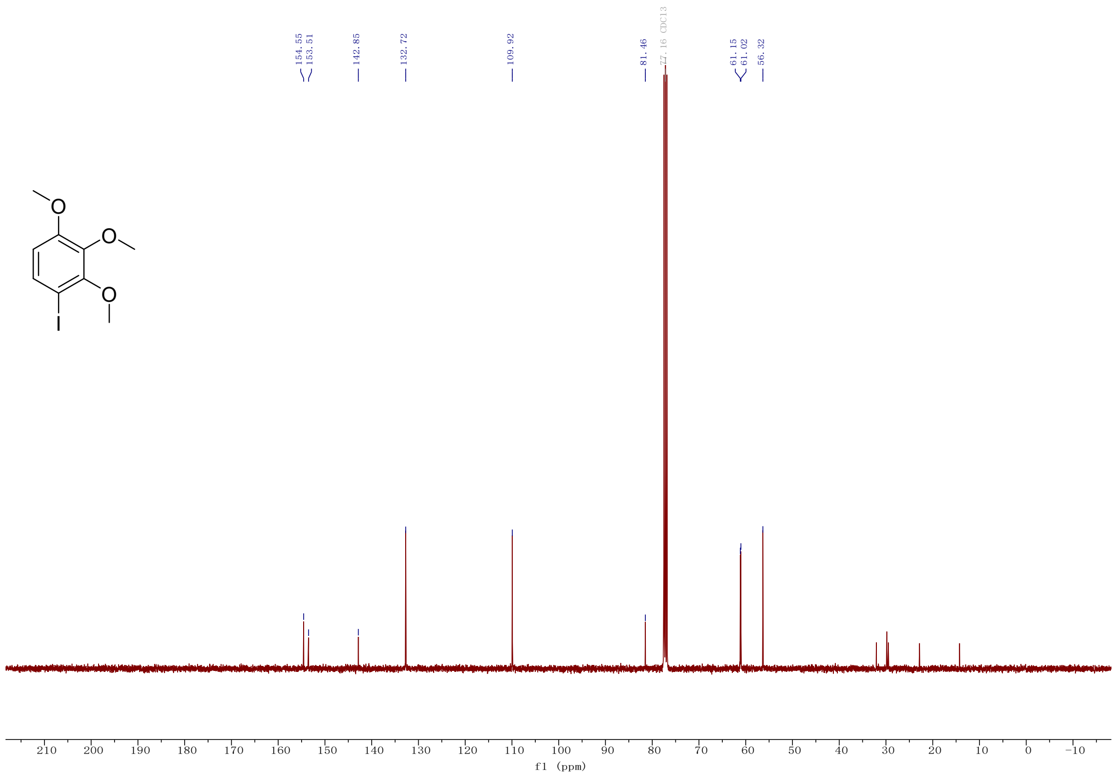

Figure S40. **^{1}H (top) and ^{13}C (bottom) NMR spectra of 1-iodo-2,3,4-trimethoxybenzene (table 3-22).**

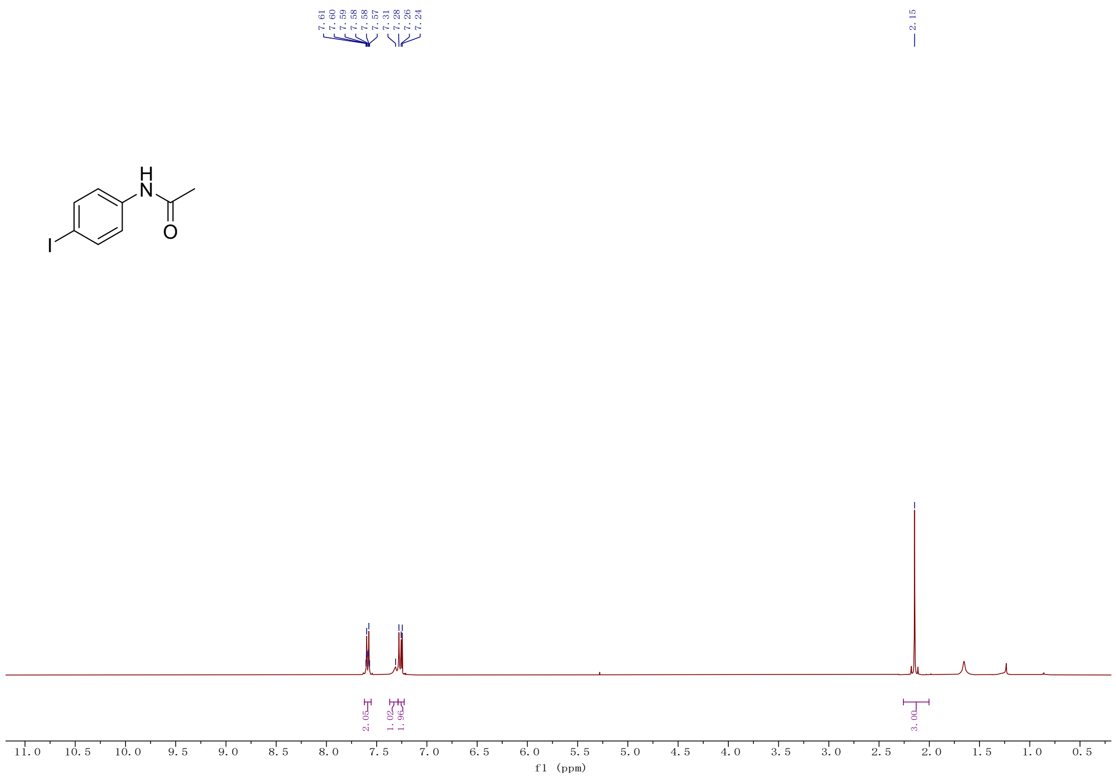

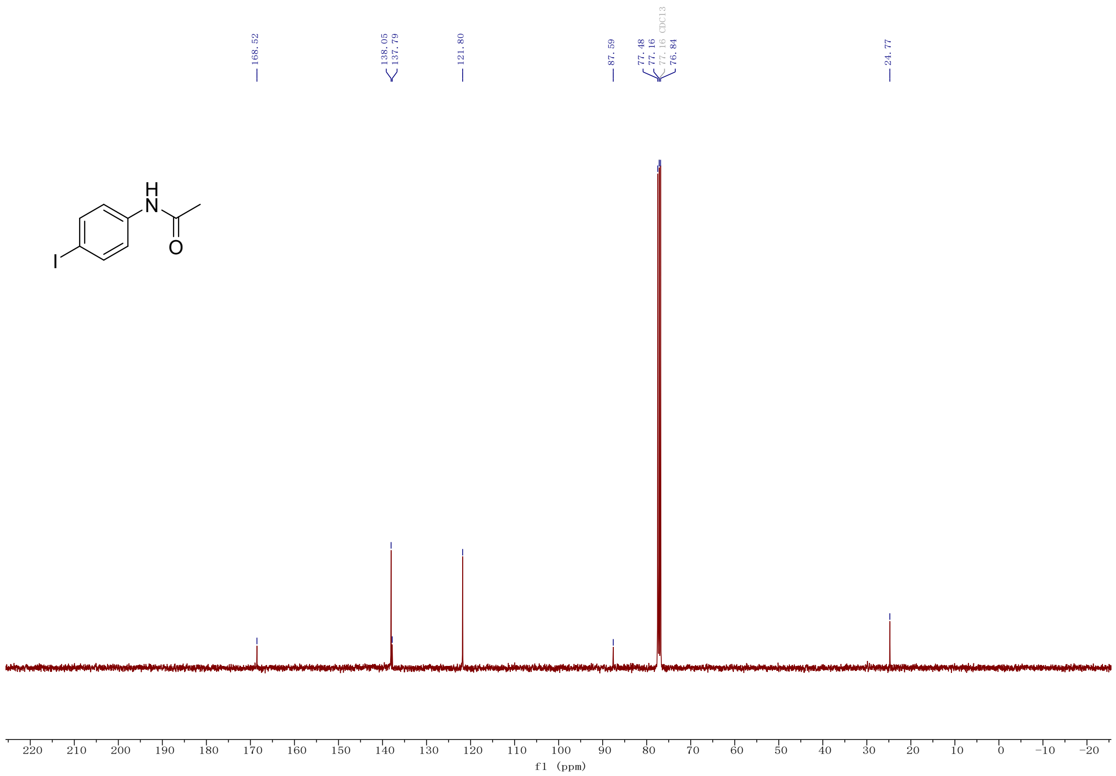

Figure S41. ^{1}**H (top) and** ^{13}**C (bottom) NMR spectra of *N*-(4-iodophenyl)acetamide (table 3-24).**
